# The Neural Correlates of Spatial Disorientation in Head Direction Cells

**DOI:** 10.1523/ENEURO.0174-22.2022

**Published:** 2022-12-16

**Authors:** Roddy M. Grieves, Michael E. Shinder, Laura K. Rosow, Megan S. Kenna, Jeffrey S. Taube

**Affiliations:** Department of Psychological and Brain Sciences, Dartmouth College, Hanover, NH 03755

**Keywords:** disorientation, head direction, navigation, single-unit recording, thalamus, vestibulo-ocular

## Abstract

While the brain has evolved robust mechanisms to counter spatial disorientation, their neural underpinnings remain unknown. To explore these underpinnings, we monitored the activity of anterodorsal thalamic head direction (HD) cells in rats while they underwent unidirectional or bidirectional rotation at different speeds and under different conditions (light vs dark, freely-moving vs head-fixed). Under conditions that promoted disorientation, HD cells did not become quiescent but continued to fire, although their firing was no longer direction specific. Peak firing rates, burst frequency, and directionality all decreased linearly with rotation speed, consistent with previous experiments where rats were inverted or climbed walls/ceilings in zero gravity. However, access to visual landmarks spared the stability of preferred firing directions (PFDs), indicating that visual landmarks provide a stabilizing signal to the HD system while vestibular input likely maintains direction-specific firing. In addition, we found evidence that the HD system underestimated angular velocity at the beginning of head-fixed rotations, consistent with the finding that humans often underestimate rotations. When head-fixed rotations in the dark were terminated HD cells fired in bursts that matched the frequency of rotation. This postrotational bursting shared several striking similarities with postrotational “nystagmus” in the vestibulo-ocular system, consistent with the interpretation that the HD system receives input from a vestibular velocity storage mechanism that works to reduce spatial disorientation following rotation. Thus, the brain overcomes spatial disorientation through multisensory integration of different motor-sensory inputs.

## Visual Abstract

**Figure:**
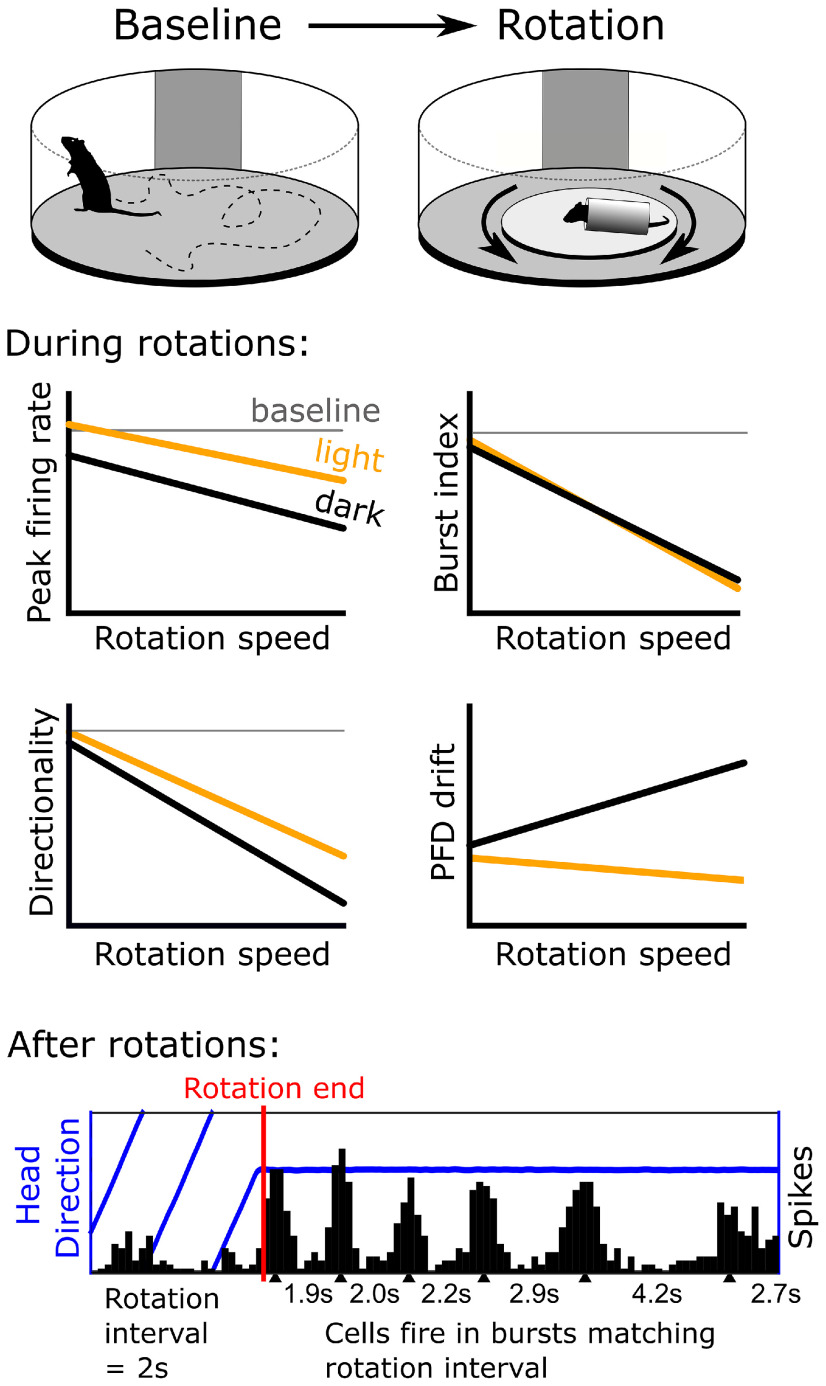

## Significance Statement

Head direction (HD) cells are neurons in the brain that underlie spatial orientation, but little is known about how these cells function during disorientation. To investigate this, we monitored HD cell responses in rats as they were rotated under a variety of conditions. We found that their activity fitted predictions from an attractor network model. We also found that visual and vestibular inputs differentially support stability and directionality respectively. Finally, we found evidence that HD cells may share a network associated with stabilizing gaze and velocity storage. Together, these findings add a missing piece to the HD system puzzle, help us understand the neural mechanisms of reorientation and will improve computational models of HD cells in the future.

## Introduction

Spatial disorientation is a state characterized by an incorrect sense of orientation and motion relative to the earth’s surface (Gillingham, 1992). Human subjects, for example, are frequently disoriented following a period of spinning that disrupts the normal functioning of the vestibular system (for review, see Previc and Ercoline, 2004). Astronauts also frequently become disoriented in microgravity or 0-g conditions, where vestibular otolith cues sensitive to gravity become less familiar compared with periods of normal upright orientation. Similar spatial disorientation phenomena, including visual reorientation and inversion illusions, contribute to at least 25–33% of all military aircraft mishaps (Gibb et al., 2011; Cheung, 2013). Despite these accidents, while the neural processes underlying normal spatial awareness are relatively well understood, less is known about how these processes function when subjects are disoriented.

Neural recordings in behaving animals have revealed several types of spatial cells including place cells, head direction (HD) cells, grid cells, and border cells (Taube, 2007; Moser et al., 2008; Grieves and Jeffery, 2017). Of these types, HD cells fire action potentials only when an animal’s head is facing in a particular direction with respect to the surrounding environment (Taube et al., 1990a, b). HD cells have been observed in a number of brain regions, including subcortical and cortical brain areas mostly related to the limbic system (for review, see Taube, 2007; Clark and Taube, 2012; Cullen and Taube, 2017; Grieves and Jeffery, 2017). Because of their directional specificity, HD cells are thought to play a key role in spatial orientation. Consistent with this view, disruption of the HD system has been linked to increased navigational errors (Gibson et al., 2013), decreased place cell functionality (Harland et al., 2017), and major disruption of grid cell firing (Winter et al., 2015).

Three forms of spatial disorientation have been recognized in the literature (Gillingham and Previc, 1996). Type I spatial disorientation involves a misperception of the subject’s orientation and is typically unrecognized by an observer. Type II spatial disorientation entails a conscious recognition by the subjects that they are disoriented, and the subjects try to become re-oriented by using any available information. Type III spatial disorientation occurs when subjects become so disoriented that they are incapacitated. This type of spatial disorientation typically occurs when subjects experience rapid and continual rotations that lead to confusion, or when subjects experience severe motion sickness or oscillopsia that makes it difficult to override these compelling conditions. Direction-specific firing of HD cells would presumably be maintained during Type I disorientation, albeit at an incorrect orientation with respect to the environment. In contrast, Types II and III spatial disorientation would be expected to lead to a disruption of normal HD cell discharge. While much is known about how HD cells respond under Type I disorientation, very little is known about how HD cells respond under conditions involving Types II and III disorientation.

Theoretical and experimental work suggests that HD cells form part of a continuous attractor network (Skaggs et al., 1995; Zhang, 1996; Goodridge and Touretzky, 2000) that is updated in part using vestibular, proprioceptive, efferent copy, and landmark cues (Stackman et al., 2003). Without visual landmark cues, HD cell stability is reduced and animals make correspondingly more errors in a spatial memory task (Mizumori and Williams, 1993). Interfering with vestibular inputs is known to disrupt HD cell firing (Stackman and Taube, 1997; Muir et al., 2009). Further, when rats climb upside down, the output of the otolith organs is distorted compared with upright locomotion (Walsh, 1960; Plotnik et al., 1999), and under these inversion conditions, HD cells lose their directional-specific firing (Taube et al., 2004; Calton and Taube, 2005; Gibson et al., 2013), and rats are unable to learn a flexible spatial task (Valerio et al., 2010). One question that arises from these findings is whether HD cell responses during inversion conditions are similar to responses when the animals are known to be disoriented.

To better understand the neural correlates and mechanisms underlying spatial disorientation and how they relate to the HD system, we monitored HD cell responses while rats underwent unidirectional or bidirectional rotations at different speeds, in the light or dark, and while passively restrained or freely moving, conditions that would promote disorientation. Under these different conditions we addressed whether anterodorsal thalamus (ADN) HD cell activity: (1) continues to fire in bursts or becomes quiescent and if this response depends on active locomotion; (2) remains directional and directionally stable, and if this stability depends on access to visual cues; (3) remains internally coherent, as expected from an attractor network; and (4) shows evidence of postrotational effects and if these effects are mitigated by the presence of visual or self-motion cues. Additionally, because both inversion and constant rotation lead to disorientation in humans (Wang and Spelke, 2000; Sargent et al., 2008) and rats (Semenov and Bures, 1989; Dudchenko et al., 1997; Martin et al., 1997; Calton and Taube, 2005), we sought to determine whether these conditions are also associated with comparable disruption to the HD system, which may point to a common underlying mechanism.

## Materials and Methods

We used three different experiment protocols.

### Experiment 1

#### Subjects

Experiment 1 used six female Long–Evans rats (Envigo). The rats were between three and six months old at the start of the study. Rats were individually housed, maintained on a 12/12 h light/dark cycle, and provided with water *ad libitum*. Throughout the experimental period animals were food restricted to lower their current weight by 10% of their presurgical weight to motivate them to forage for food pellets during recording sessions.

#### Apparatus

We used two types of apparatus, the first was a standard recording cylinder (76-cm diameter, 51 cm tall) painted gray with a polarizing white cue card affixed to the inside wall that spanned 100°. The cylinder floor was composed of a single sheet of gray paper and changed in between recording sessions. The second was a “disorientation apparatus” which consisted of a 68.5-cm diameter circular wooden platform, painted gray and raised 38 cm from the floor on a tripod base. The platform was mounted on a lazy Susan and could be freely rotated. A low (12.7 cm) wall around the edge of the platform prevented animals sliding over the edge, particularly during the rotation sessions. During all recording sessions the disorientation apparatus was placed in the center of the room and surrounded by a black curtain 2.5 m in diameter that stretched from the floor to the ceiling.

#### Data acquisition

For single-unit screening, the animal was attached to a multi-wire recording cable that was connected on one end to an overhead commutator (Biela Idea Development) and to the animal’s headstage on the other end. Signals were passed through a field-effect transistor (FET) in a source-follower configuration, amplified (Grass Instruments P511), band-passed filtered (300–10 000 Hz, 3 dB/octave; Peavey Electronics PME8), and sent through a series of window discriminators (Bak Electronics DDIS1) before being displayed on an oscilloscope (Tektronix 5113). Spike discharge was sampled at a rate of 60 Hz and stored for offline analysis (National Instruments DIO-32, Macintosh IIfx).

For recording, in addition to the above, an automated video-computer tracking system (Eberle Electronics) monitored neuronal discharge while simultaneously tracking the positions of two light-emitting diodes (LEDs; one red, one green) secured to the animal’s head. The red and green LEDs were spaced 10 cm apart along the midline of the animal’s body axis and positioned over the rat’s snout and back, respectively. The LED positions and spike discharge were sampled at a rate of 60 Hz and stored for offline analysis (National Instruments DIO-32, Macintosh IIfx).

#### Recording procedure

If a HD cell was identified on one or more of the ten recording channels, we isolated the cell’s waveform using a series of window discriminators (Bak Electronics) and conducted a recording session. The rat’s HD was determined by a two-spot video tracking system (Eberle Electronics) that monitored the location of two differently colored, light-emitting-diodes (LEDs; one red, one green) attached to the animal’s head stage.

We recorded a total of 25 HD cells. The following eight manipulations were conducted in the order described. No more than 8 min, but usually less than 2 min, passed between sessions. (1) Visual baseline 1: this 8-min session was conducted in a standard recording cylinder with a polarizing cue card and with the lights on. This session served as a baseline control for the HD cell during normal visual conditions. (2) Dark baseline: this session was 4 min long and was conducted in the same cylinder apparatus. Before the session began, the rat was blindfolded, and we changed the floor paper. The cue card was removed, and the lights were turned off. (3) Dark slow rotation: this session was 2 min long and was conducted in the disorientation apparatus. The rat remained blindfolded and the platform was manually spun at a slow rate above the vestibular threshold (mean ± SD; 111 ± 38°/s) in alternating directions (back and forth every 5–10 s). In this session and all other rotation sessions, the same experimenter rotated the platform to ensure a consistent spin rate and they changed their position around the apparatus at least three times during each session. (4) Dark fast rotation: this session was 2 min long and was conducted in the disorientation apparatus. The rat remained blindfolded and the platform was rotated at a medium rate (mean ± SD; 195 ± 40°/s) in alternating directions. (5) Dark baseline 2: this session was 4 min long and was conducted in the cylinder described before. The rat remained blindfolded and the floor paper was changed to remove olfactory cues. The lights remained off and the cue card remained absent from the cylinder. (6) Visual baseline 2: this session was 4 min long and was conducted in the cylinder described before. These sessions followed the same protocol as visual baseline 1; the rat was not blindfolded, the lights were on, and the cue card was returned to its original orientation. (7) Visual slow rotation: this session was 2 min long and was conducted in the disorientation apparatus. A white cue-curtain was hung on a portion of the floor-to-ceiling black curtain surrounding the disorientation apparatus and the lights were switched on. Otherwise, conditions were the same as those described for the Dark slow rotation sessions. (8) Visual fast rotation: this session was 2 min long and took place in the disorientation apparatus with the cue-curtain in place and the lights switched on. Otherwise, conditions were the same as those described for the Dark slow rotation sessions.

Not all animals completed all eight session types. Further, a subset of recording sessions suffered from missing position tracking data and were excluded from the analyses (37/172 or 21.5% of sessions). A summary of the sessions recorded for each cell is shown in Extended Data Figure 1-1.

**Figure 1.**
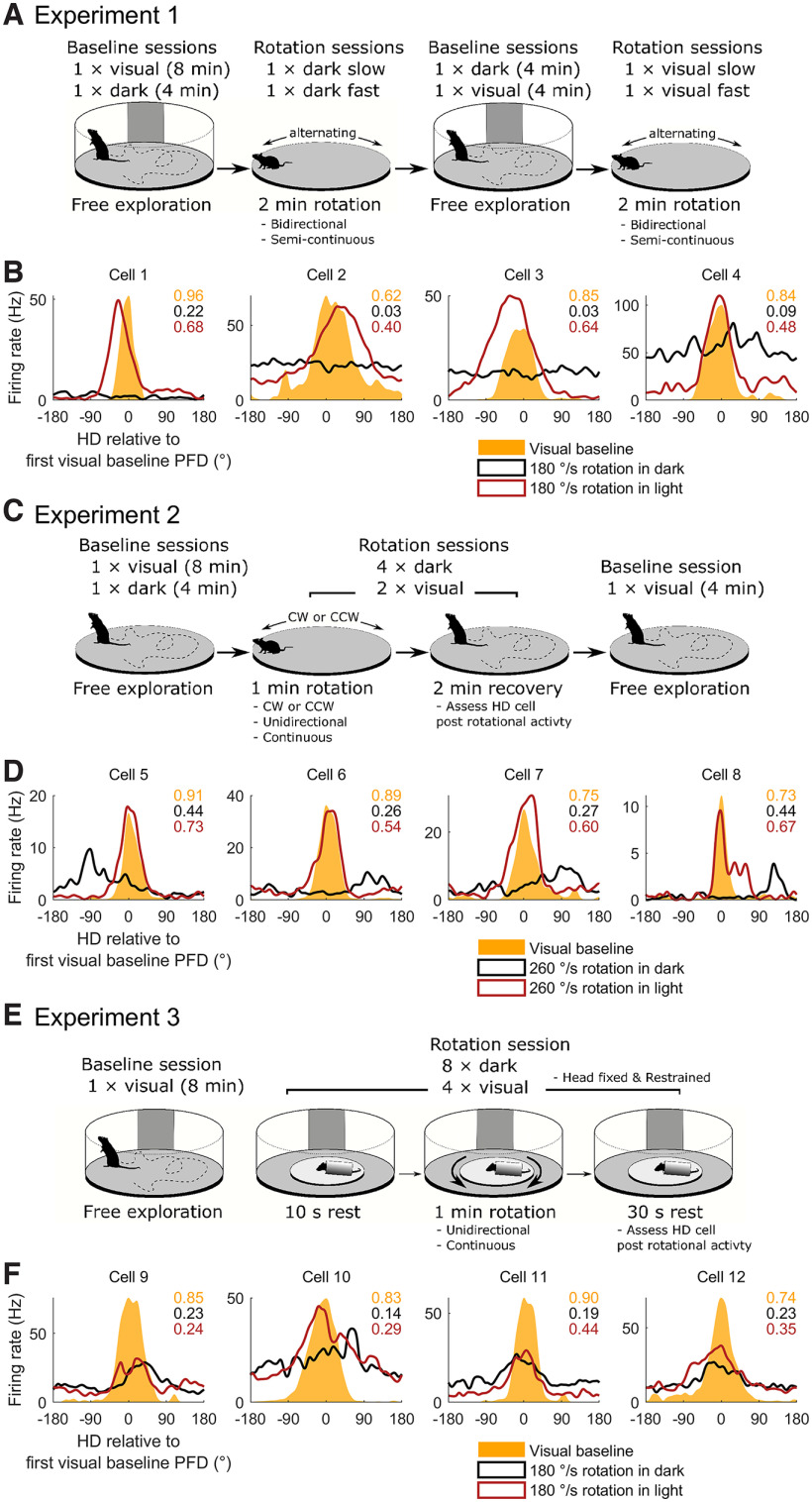
Design of Experiments 1–3 and example cells. A summary of the sessions recorded for each rat and cell is shown in Extended Data Figure 1-1. Visual = sessions conducted under illumination, dark = sessions conducted in darkness and while rats were blindfolded. CW = clockwise, CCW = counterclockwise, PFD = preferred firing direction. ***A***, Recording protocol for Experiment 1. Slow rotations were performed at 111°/s, fast rotations at 195°/s. Average rotation speeds for all three experiments can be seen in Extended Data Figure 1-2. ***B***, Tuning curves for four example cells recorded in a baseline session with the lights on (yellow shaded curve) a rotation session in the dark (black curve) and a rotation session in the light (red curve). The *x*-axis is normalized so that an angle of zero denotes the cell’s PFD in the visual baseline session. Top right text gives the Rayleigh vector length for the tuning curve of the same color. HD cell baseline tuning curves for all three experiments can be seen in Extended Data Figures 1-3, 1-4, and 1-5, respectively. ***C***, ***D***, Same as ***A***, ***B*** but for Experiment 2. All rotations in this experiment were performed at 260°/s. ***E***, ***F***, Same as ***A***, ***B*** but for Experiment 3. All rotations in this experiment were performed at 180°/s. Representative histology for all three experiments can be seen in Extended Data Figure 1-6.

10.1523/ENEURO.0174-22.2022.f1-1 Extended Data Figure 1-1 Session and animal summary tables. ***A–C***, Session and cell breakdown for Experiments 1–3. “Firing & direction” denotes sessions where cell spiking statistics (i.e., burst index and firing rate) and directional statistics (i.e., Rayleigh vector length and PFD drift) were analyzed, “firing only” denotes sessions where only cell spiking statistics were analyzed. In panel ***C***, condition descriptors for session type denote (from left to right): the starting angle relative to the cell’s PFD (0, 90, 180, or 270), rotation direction (CW or CCW), and illumination condition (light or dark). Download Figure 1-1, TIF file.

### Experiment 2

#### Subjects

Experiment 2 used three female Long–Evans rats (Envigo). The rats were three to six months old at the start of the study. Housing, light-dark cycle, food restriction, and water availability were the same as in Experiment 1.

#### Apparatus

The same platform apparatus and room set-up that was used in Experiment 1 was used for all the baseline and rotation sessions. Only the platform apparatus was used in this experiment, including for the baseline session; the cylinder apparatus was not used for any sessions.

#### Training and habituation

Following one week of food restriction rats were habituated to the apparatus. For three sessions rats were placed in pairs for 20 min on the platform, and foraged for 20-mg sucrose pellets (Noyes) scattered on the floor. After these sessions the rats were then trained separately for 20 min/d and the sugar pellets were dropped in a random fashion from a food dispenser attached to the ceiling (Goodridge et al., 1998). The rats were fully trained when they spent 80% of their time foraging. Following training, the rats were habituated to wearing a blindfold (Whishaw and Maaswinkel, 1998) for 10 min/d in their cage and for 2 min/d in the disorientation apparatus. Blindfold habituation continued until the rats spent <20% of their time manipulating the blindfold.

#### Data acquisition

Data acquisition methods and equipment were identical to those described for Experiment 1.

#### Recording procedure

Sessions were composed of nine sessions conducted in the following order. (1) Visual baseline 1: animals were recorded while they explored the platform apparatus for 8 min while foraging for sugar pellets. There was no rotation, the rats did not wear a blindfold, and the room lights were switched on. (2) Dark baseline 1: these differed from visual baseline 1 only in that the rat was blindfolded, the room lights were switched off, and the session’s duration was 4 min. (3–6) Dark rotation sessions: rats were blindfolded and the room lights were switched off. The experimenter rotated the platform manually at a fast speed (mean ± SD; 269 ± 41°/s) and constant direction for 1 min and then stopped abruptly. Each session was followed by 2 min of free movement with no rotation. Half of the sessions were clockwise (CW) rotations and half were counterclockwise (CCW) rotations. (7, 8) Visual rotation sessions: these sessions were identical to the dark rotation sessions, except rats were not blindfolded and the room lights were switched on (mean rotation speed: 260 ± 43°/s). CW and CCW were balanced across these two sessions. (9) Visual baseline 2: these sessions were identical to visual baseline 1, but their duration was 4 min. In the rotation sessions the combination of blindfolding and rotating the rat was considered sufficient to disorient it and is consistent with and comparable to previous methods of producing disorientation (Goodridge et al., 1998; Knierim et al., 1998). A summary breakdown of the sessions recorded for each cell is shown in Extended Data Figure 1-1.

### Experiment 3

#### Subjects

Experiment 3 used six female Long–Evans rats (Envigo). The rats were three to six months old at the start of the study and housing, and other conditions were similar to those of Experiments 1 and 2.

#### Apparatus

The apparatus consisted of two items. The first apparatus was the standard recording cylinder described above and was surrounded by a floor-to-ceiling black curtain. Sugar pellets (20 mg; Bioserve, no. F0071) were automatically dropped to a semi-random location in the enclosure every 30 s. The visual landmark cue attached to the inside cylinder wall (a sheet of white cardboard) was not moved from its relative position throughout the experiments. The second apparatus consisted of the small platform mounted on a circular bearing, forming a lazy Susan turntable as described above, except the rotation of the platform was now controlled by a motor. Rats were restrained by wrapping them in a cloth with bungy cords and then placed into a Plexiglass tube (6 cm in diameter) and their heads were fixated via an implanted head bolt to a mounted bar that was attached to the set-up (see Stackman et al., 2003 for further details). During recording the restraint device was placed within the cylindrical enclosure on top of the platform so that the visual surround remained visible. The axis of rotation was aligned with the estimated center of the intra-aural axis of the animal’s head. In this setup, we can mostly exclude all movement from locomotion, neck-on-body proprioception, or voluntary head movements.

#### Training and habituation

All animals were acclimated to restraint before any electrophysiological recordings. Starting one week following postsurgical recovery, the animals were restrained for 8 min by holding them loosely in the experimenter’s hands. This procedure was first done once a day for 3 d. Then, as electrophysiological screening began, the animal was restrained following the screening session for 8 min using a towel-wrapped restraint technique (Taube, 1995; Golob et al., 1998). The towel-wrapped restraint acclimation was repeated once a day for 3 d. Following the subsequent screening session, the animal was placed in a restraint device with its head and body restrained for 8 min (Shinder and Taube, 2011). The head-fixed restraint acclimation continued for at least 3 d before neurons were tested with the animal in the head-fixed restraint device. Following this procedure, animals tolerated restraint without significant periods of attempted movement. While restrained, bands were loosely placed around the body to hold the animal’s forelimbs and hindlimbs. The rat could remove these bands by struggling against the restraint, but this behavior was rarely noted and resulted in the immediate cessation of experimentation for that session.

#### Data acquisition

Data acquisition was the same as described for Experiments 1 and 2, except that spike events were sampled at 10-μs resolution.

#### Recording procedure

Sessions were composed of three session types. (1) Visual baseline 1: animals were recorded while they explored the platform apparatus for 8 min. There was no rotation, the rats did not wear a blindfold, and the room lights were switched on. (2) Dark rotations: the rats were restrained and head-fixed in the rotation apparatus, they were blindfolded, and the room lights were switched off. Recordings consisted of 10 s of immobility without rotation, 60 s of continuous rotation, either CW or CCW, followed by a further 30 s of immobility without rotation. Room lights were switched on and the animal’s blindfold was removed between recordings of different cells. (3) Visual rotations: these sessions were identical to dark rotations except that the room lights were switched off and the rats were not blindfolded. Sessions always included at least eight dark rotation sessions (four CW and four CCW) and these sessions started with the rat positioned facing 0°, 90°, 180°, or 270° from the target HD cell’s preferred firing direction (PFD). A subset of sessions also included four visual rotation sessions with the same starting positions. A summary of the sessions recorded for each cell is shown in Extended Data Figure 1-1.

### General methods

#### Animal care

All procedures were conducted according to institutionally approved animal care protocols, were in accordance with the American Physiologic Society’s *Guiding Principles in the Care and Use of Animals*, were approved by the institutional care and use committee at the host institution, and adhered to the standards outlined by the National Institutes of Health *Guide for the Care and Use of Laboratory Animals* and the Society for Neuroscience. Surgery was conducted under aseptic conditions and animals were allowed to recover for 7 d before screening started.

#### Electrodes and surgery

Electrodes consisted of a bundle of 10 25-μm diameter nichrome wires threaded through a stainless-steel cannula and attached to a modified 11 pin Augat (Experiments 1 and 2) or Mill-Max (Experiment 3) connector. This assembly was embedded in dental acrylic and could be lowered using three screws forming a tripod support for the connector and acrylic (Kubie, 1984).

Following training, rats returned to ad-lib feeding for 4–6 d before surgery. Rats were anesthetized with ketamine (0.3 ml/100 g followed by an additional 0.1 ml if necessary). Surgery was performed using standard stereotaxic procedures: a 10-wire drivable microelectrode (Kubie, 1984) was implanted into the anterodorsal nucleus (ADN) of the thalamus (1.5 mm posterior to bregma, 1.3 mm lateral to the midline, and 3.7 mm ventral to brain surface; Paxinos and Watson, 2006). Each rat was given the analgesic, Buprenex (0.2–0.3 ml), immediately after their surgery and a supplement (0.2 ml) the following day.

#### Screening

Following 7 d of recovery, each rat’s electrodes were monitored for cellular activity while the rat foraged for sugar pellets in the cylindrical screening environment. Putative HD cells were identified by monitoring activity on the microelectrodes in conjunction with the rat’s behavior and directional heading. If HD cell activity was not found, the entire electrode assembly was advanced 30–120 μm ventrally and the process repeated after at least 4 h (usually 24 h).

#### Cell recording

All cell firing was isolated using a series of window discriminators. For Experiments 1 and 2, all cell waveforms that were “accepted” and passed through the window discriminators were counted within each video frame (16.667 ms), and these data were captured by data acquisition software (LabView) and stored on a Macintosh computer. For Experiment 3, cell spikes were also captured by the window discriminators, timestamped from the beginning of the session (Eberle Electronics), and acquired by data acquisition software (LabView). The timestamps were then stored on a Macintosh computer and analyzed off-line. For all experiments, the locations of a red and a green LED that were attached either to the rat’s headstage (spaced 10 cm apart along the midline of the animal’s body axis and positioned over the rat’s snout and back, respectively) or to the restraint device were monitored at 60 Hz and stored on the computer and analyzed off-line at a later time. Monitoring the positions of the two LED enabled us to track the rat’s HD.

#### Data preparation

Spikes times or spike counts, along with position (LED) tracking data, were exported to MATLAB (2021a, The MathWorks) using LabView (v3.1, National Instruments). Tracking errors such as LED swapping were removed and position data were simultaneously interpolated and smoothed using an unsupervised, robust, discretized, n-dimensional spline smoothing algorithm (MATLAB function *smoothn*; Garcia, 2010, 2011). This algorithm was applied to the two tracking LEDs separately and instantaneous HD was then estimated as the angle between these points. In Experiment 2, some sessions suffered from consistent periods of missed tracking when the animal rotated out of view of the camera. These sessions were excluded from the directional analyses (i.e., Rayleigh vector, PFD), but not from spiking activity analyses (i.e., firing rate, burst index) based on manual inspection of each session.

#### Session phase detection

In Experiment 2, the speed of the rotating platform was monitored by an additional camera device that monitored the movement of black and white dashes on the inside portion of the bicycle wheel as it rotated. These data were smoothed with a 10 point (1/6 s) median box filter. The first and last time points when the platform rotated at a speed >∼130°/s were taken as the beginning and end of the rotation period, respectively.

For Experiment 3, or sessions from Experiment 2 missing the separately recorded rotation data, we calculated the change in HD per second and smoothed this value using a moving average box filter with a 60-point (1-s) window width. The first and last time points when the platform rotated at a speed >∼60°/s were used as the beginning and end of the rotation period, respectively.

#### Postrotation behavior bias

After rotations in Experiment 2, rats were unrestrained during the 2-min recovery period. To determine whether animals showed a directional bias in their movements during the recovery period, we compared the sign (±) of the cumulative angular deviation (MATLAB function *angdiff*) in the first 30 s of the recovery phase to the sign (±) of the cumulative angular deviation in the whole 30-s rotation phase. The same sign (±) in both would indicate that an animal continued to move its head, on average, in the same direction as in the previous rotation.

To calculate the chance of these values being equal we compared the sign (±) of the cumulative angular deviation (MATLAB function *angdiff*) in the first 30 s of the recovery phase to the sign (±) of the cumulative angular deviation in a random 30-s period taken from the first visual baseline session, before initiation of rotation. We repeated this process 1000 times per session and estimated the probability of the outcome by z-scoring the original mean difference to the shuffled values and calculating a probability as the position of the z-value (two-sided test) in the cumulative distribution function of a normal distribution with mean 0 and SD 1. This method accounts for possible biases in the animal’s behavior that were not because of rotation.

#### Parameter normalization

To compare statistical parameters (i.e., peak firing rate, directionality) across the three experiments, we normalized the values by z-scoring them relative to their values from the first visual baseline session:

$$norm.=\frac{\alpha -\mu_{baseline}}{\sigma_{baseline}},$$

where norm is the normalized parameter, α is the raw parameter, $\mu_{baseline}$, and $\sigma_{baseline}$ are the mean and SD of the parameter values from the first visual baseline session obtained for that experiment. Raw values and comparisons within experiments are shown in Extended Data Figure 2-1. PFD drifts were normalized by calculating the angular deviation between PFDs in each session and the first visual baseline session obtained for that experiment (MATLAB function *angdiff*).

**Figure 2.**
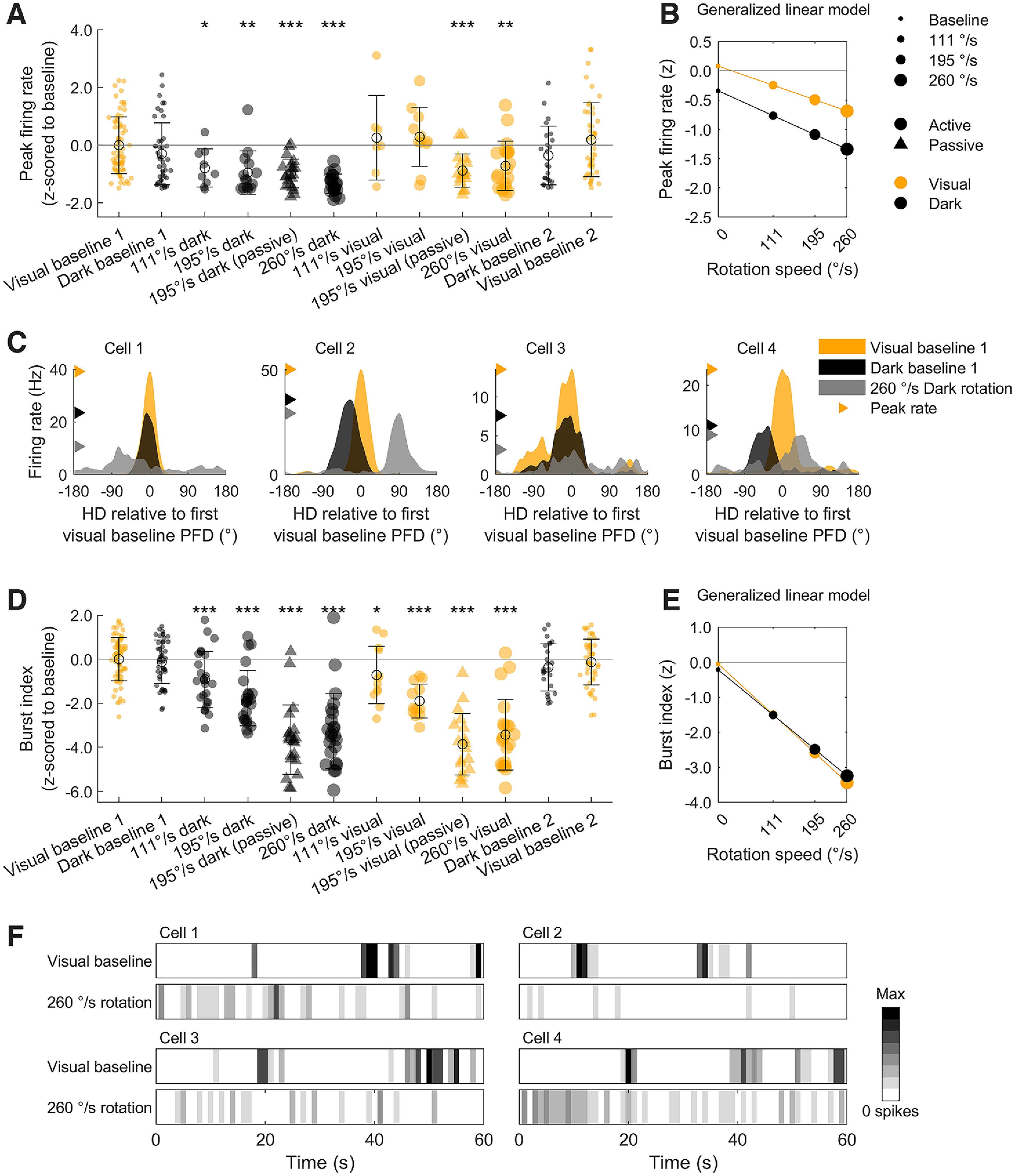
Peak firing rate and burst index decreased during rotations. For panels ***A*** and ***D***, group *n* = 71, 48, 25, 25, 23, 23, 25, 25, 23, 23, 25, 48. ***A***, Peak firing rates for all HD cells in every experimental condition (see legend on far right). Values are z-scored relative to the first visual baseline session in each experiment. Zero denotes the mean baseline value and values less than zero indicate a drop from baseline. Top text shows the result of Holm–Bonferroni corrected *t* tests comparing each group to the first visual baseline session (*p < 0.05, **p < 0.01, ***p < 0.001). Raw directional and spiking values, compared within experiments, can be seen in Extended Data Figure 2-1. ***B***, The linear relationship between rotation speed, darkness, and peak firing rate (for a hypothetical animal that is actively locomoting and rotating unidirectionally), extracted from a GLM fit to the data in ***A***. AHV modulation does not explain the effects shown here (Extended Data Fig. 2-2). ***C***, Tuning curves for four HD cells that showed a decreased peak firing rate in the fastest rotation condition (260°/s) compared with the baseline sessions. PFD = preferred firing direction. ***D***, ***E***, Same as ***A***, ***B*** but for burst index. ***F***, Spike histograms for four HD cells in a visual baseline session (top) and during the fastest rotation condition (bottom; 260°/s). Cell histograms are scaled to the same color axis. Spikes in the visual baseline sessions group together in bursts (dark bands separated by empty bins) more than those during rotation.

10.1523/ENEURO.0174-22.2022.f2-1 Extended Data Figure 2-1 Raw parameter values for all three experiments and statistical comparisons within experiments. Horizontal lines denote a significant *post hoc* comparison (*p *< 0.05; Dunn–Sidak corrected). For panels ***D***, ***H***, and ***L***, drift values are relative to the first visual baseline session (i.e., 90° denotes a 90° CCW drift away from baseline) and the text gives the result of Holm–Bonferroni corrected v-tests for nonuniformity around a mean direction of 0°, a significant value here denotes clustering around 0° and thus a stable PFD (n.s. = *p *> 0.05, *p < 0.05, **p < 0.01, ***p < 0.001). ***A***, Peak firing rates in Experiment 1 (*F*_(7,127)_ = 2.3, *p *= 0.0317, ${\text{η}}^{2}$ = 0.11). ***B***, Burst index in Experiment 1 (*F*_(5,116)_ = 10.3, *p *< 0.0001, ${\text{η}}^{2}$ = 0.31). ***C***, Directionality in Experiment 1 (*F*_(5,82)_ = 17.6, *p *< 0.0001, ${\text{η}}^{2}$ = 0.52). ***D***, PFD stability in Experiment 1. ***E***, Peak firing rates in Experiment 2 (*F*_(6,138)_ = 7.2, *p *< 0.0001, ${\text{η}}^{2}$ = 0.24). ***F***, Burst index in Experiment 2 (*F*_(4,104)_ = 5.3, *p *= 0.0006, ${\text{η}}^{2}$ = 0.17). ***G***, Directionality in Experiment 2 (*F*_(6,138)_ = 112.2, *p *< 0.0001, ${\text{η}}^{2}$ = 0.83). ***H***, PFD stability in Experiment 2. ***I***, Peak firing rates in Experiment 3 (*F*_(2,58)_ = 11.4, *p *= 0.0001, ${\text{η}}^{2}$ = 0.28). ***J***, Burst index in Experiment 3 (*F*_(2,58)_ = 55.3, *p *< 0.0001, ${\text{η}}^{2}$ = 0.66). ***K***, Directionality in Experiment 3 (*F*_(2,58)_ = 68.8, *p *< 0.0001, ${\text{η}}^{2}$ = 0.70). ***L***, PFD stability in Experiment 3. Download Figure 2-1, TIF file.

#### Generalized linear model (GLM)

After normalizing parameters for testing (see above, Parameter normalization) we fitted a generalized linear model (GLM) to the data to determine the effects of each experimental manipulation across experiments. These fits were conducted in MATLAB (function *fitglm*) with the following model:

y=a + b + c + d + (a×b),

where a is the rotation speed in °/s (baseline sessions were assigned a rotation of 0°/s), b denotes the presence or absence of visual cues, c denotes whether animals were free to move or head-fixed (baseline sessions were categorized as freely-moving), d denotes whether rotation sessions were unidirectional or bidirectional (all sessions were categorized as bidirectional except unidirectional rotation sessions), and (a×b) represents the interaction between rotation speed and darkness. In addition, we included an intercept for the model, assigned a normal distribution to the response variable, and used an identity link function. To calculate the standardized coefficients reported in text, these predictor variables were z-scored (MATLAB function *zscore*) before calculating the fit. The statistical deviation of the model from a constant fit was calculated using the MATLAB function *coefTest*. The R^2^ values reported were the “ordinary” values calculated by *fitglm*. Finally, the model representations reported in the text were calculated using the above model equation with a set approximately to the three rotation speeds used in our experiments (111, 195, and 260°/s), c was set to freely-moving and d was set to a unidirectional rotation.

#### Tuning curves

HD tuning curves (HD × firing rate plots) were calculated as the ratio of spikes emitted and time spent facing each direction. First, a HD dwell time map was calculated as the circularly kernel smoothed density estimate of all sampled HDs (MATLAB function *circ_ksdensity*, kernel width = 0.1 radians; Berens, 2009) across 360 bins spanning 0–360°. A spike density map was then generated in the same way for all spike HDs and a tuning curve was calculated as the ratio of these two maps. For rotation sessions, we generated tuning curves for the whole session as well as for the rotation and recovery phases separately.

#### Windowed tuning curves and parameters

To estimate the time course of changes in each of the parameters, such as directionality, throughout rotation, we split sessions into nonoverlapping 10-s windows. Each window was extended by 2 s until at least 90% of the directional bins were sampled. We then generated a tuning curve for each window (see above, Tuning curves) and calculated parameters such as peak firing rate (see below, Directional statistics) for each one.

#### Directional statistics

From the HD tuning curves we calculated, as a measure of directionality, the Rayleigh mean vector length (MATLAB function *circ_r*; Berens, 2009). Peak firing rate was defined as the maximum firing rate value found in the HD tuning curve and preferred firing direction (PFD) as the direction associated with this peak.

Next, for each cell we shuffled its spike train 100 times by random increments of 0.02 s (minimum 20 s) and for each shuffle recomputed a directional firing rate map and statistics as above. A cell was categorized as directionally modulated if it exhibited a Rayleigh vector greater than the 95th percentile of the shuffled values in the first visual baseline session and fired at a peak rate >5 Hz (across all cells: min = 8 Hz, median = 35 Hz).

#### Angular head velocity (AHV)

It is well known that angular head velocity (AHV) correlates positively with cell firing rates for ADN HD cells (Taube and Muller, 1998; Taube, 2007), such that cells fire at higher firing rates within the PFD for faster head turns (higher angular head velocities). Further, Shinder and Taube (Shinder and Taube, 2011) explored the possibility that AHV influenced HD cell peak firing rates during passive rotations in head-fixed, restrained rats, but did not find it had a significant effect. To test whether differences in AHV might explain the decreases we observed in peak firing rates during the rotation and disorientation sessions, we determined the average and peak firing rates predicted for every rotation session based on the HD and AHV modulation observed in the first visual baseline.

AHV was estimated as the change in HD per second smoothed using a moving average box filter with a three-point (0.05 s) window width. To determine whether decreases in peak firing rate were the result of AHV sampling we generated HD × AHV tuning curves for each cell in the first visual baseline. We first generated a HD×AHV spike map as the bivariate histogram of spike values with 6° HD bins spanning the 0–360° range and 6°/s AHV bins spanning the −300 to +300°/s range. This map was then smoothed with a 1.5 σ Gaussian filter (MATLAB function *imgaussfilt* with 3 × 3-pixel size filter). We generated a dwell time map by repeating this process for position data. A firing rate map was calculated by dividing the spike map by the dwell map multiplied by the position sampling interval. A map of firing probability was calculated as the ratio of the spike and dwell map. In both cases, bins containing <0.1 s of position sampling were treated as unvisited HDs.

Next, we took HD×AHV dwell time maps for each session and multiplied them with the firing probability map for the first visual baseline session. The resulting “spike” maps provide a prediction of the spiking that should occur in that session given the HD×AHV in that session and the HD×AHV modulation observed in the first baseline session. We took the sum of these maps as the total number of spikes predicted for the session. From these maps we calculated a spike rate index as:

spike rate index=(α−β)/(α + β),

where α is the sum of the observed spike map (the actual number of spikes recorded in a session) and β is the sum of the predicted spike map (the total spikes predicted using the above process). Bins treated as unvisited in either map were treated as unvisited in both maps. Low spike rate index values indicate that fewer spikes were recorded than predicted, while high spike rate index values indicate the opposite. Values of zero indicate that a cell fired at exactly the rate expected. A schematic of this process is shown in Extended Data Figure 2-2*A–C*.

We similarly calculated actual and predicted HD tuning curves by summing columns of observed and predicted spike maps and dividing them by their total occupancy; examples of these tuning curves are shown in Extended Data Figure 2-2*C*. From these tuning curves we calculated a peak rate index as:

peak rate index=(α−β)/(α + β),

where α is the peak firing rate in the actual HD tuning curve and β is the peak firing rate in the predicted HD tuning curve. As before, bins treated as unvisited in either map were treated as unvisited in both maps. Low peak rate index values indicate that peak firing rates were lower than predicted, while high peak rate index values indicate the opposite.

#### Rotation and inversion comparisons

We sought to compare firing statistics, such as interspike intervals (ISIs) and burst index, between our rotation sessions and previously published data where rats were inverted (upside down). We only used spike data from Experiment 3 as these were recorded at the highest resolution (10 μs) and animals were head-fixed which was also the case during the inversion experiments. Inversion data and their upright control comparisons were taken from Shinder and Taube (Shinder and Taube, 2019) manipulations 1, 5, 7, 11. Briefly, in these experiments, rats were head-fixed, restrained and rotated to be upside down while also facing a HD cell’s PFD. The same cells were also recorded while rats faced the recorded cell’s PFD in the same setup but in an upright position.

Because the data we used from the inversion experiments only included periods when the rats were facing a cell’s PFD, for comparison with the rotation disorientation data, we only included the spikes that were emitted when rats were facing within ±30° of the cell’s PFD. Only ISIs calculated during, not between, these periods were included in the ISI and burst index analyses. We also excluded spikes emitted during the first six rotations to focus on activity during full disorientation. In both cases we also excluded cells exhibiting <10 ISIs. For both experiments cells were often recorded for different numbers of sessions, and values were therefore averaged (i.e., burst index and average ISI duration) across sessions of the same type (i.e., inversion or dark rotation).

ISIs were calculated as the amount of time between consecutive spike pairs (MATLAB function *diff*); for this comparison, burst index was defined as the proportion of ISIs with a duration <25 ms, PFD firing rate was defined as the total number of spikes emitted while facing the PFD (or ±30° of the PFD in the case of rotation sessions) divided by the total time spent facing this direction.

To compare values between the two experiments, we normalized data by z-scoring them relative to baseline values. Baseline sessions were active foraging sessions for the rotation sessions or upright sessions for the inversion sessions. After this normalization, the baseline values were combined and both experiments were plotted and compared with one another.

To account for differences in PFD firing rates, we performed the above analyses a second time after matching each experimental and baseline group in terms of firing rate. To perform this analysis, for each cell’s PFD firing rate we found the nearest neighbor in the baseline PFD firing rate group within a maximum distance of ±1 Hz (MATLAB function *knnsearch*). The data corresponding to this nearest neighbor were then substituted for the cell’s original data for all parameters (ISI duration, burst index). Cells with no nearest neighbor within the maximum distance were excluded, which reduced the group sizes. This approach allows repetitions of baseline values if they match multiple cells. Once this analysis was completed, groups were z-scored according to their baseline values and analyses continued as described above.

#### Burst index

As in Yoder and Taube (2009), we calculated a burst index score to represent the proportion of time during which a cell fired in high-frequency bursts or was inactive relative to the time during which action potentials occurred at a relatively constant rate. For this measure, spikes were sorted into 1-s bins from the beginning to the end of a recording session. The burst index score was then defined as follows:

$$Burst\;index=\frac{\left(total\;bins>1.75R\right)+\left(total\;bins<0.25R\right)}{total\;bins},$$

where R is the overall mean firing rate. Burst index values can range between 0 and 1, with a value of 0 indicating a firing rate that remains near the mean rate for the entire session and a value of 1 indicating the cell either remains silent or fires near its maximal rate for the entire session (i.e., often fires in bursts).

#### Circular-linear regression

In Experiment 3, HD cell PFDs tended to shift later in rotation phase with each revolution. To investigate this further, we split sessions into nonoverlapping 360° (one revolution) windows. We then generated a tuning curve for each window (see above, Tuning curves) and calculated the PFD of each window (see above, Directional statistics). Because directionality and firing rates decreased significantly after 6–12 rotations, we concentrated on only the first six rotations for this analysis.

Next, we used circular-linear regression to calculate the slope of these PFDs (MATLAB function *CircularRegression*; Zugaro, 2018) using a method described previously (Kempter et al., 2012). If PFDs shifted consistently in the direction of rotation, we would expect positive slopes for CCW shifts during CCW rotations and negative slopes for CW shifts during CW rotations. To test this prediction, we grouped regression slopes according to rotation direction and tested their deviation from zero using one-sided *t* tests (MATLAB function *t* test).

#### Spike bursts

To detect bursts of spikes we calculated the instantaneous firing rate of a cell as a kernel smoothed density estimate of spike counts (MATLAB *fitdist*, 20-ms bin size, Gaussian kernel with a 10-bin bandwidth). We then found peaks in this density estimate (MATLAB *findpeaks*, minimum peak prominence of 0.25, minimum peak height of 0.25 spikes and a minimum interpeak distance of 0.05 s).

#### Post-rotational bursting: time constant

To estimate a temporal decay constant for the vestibular system based on the activity of the HD cells postrotation, we measured the time between consecutive bursts occurring after rotations had ended. We converted these interburst intervals to an estimate of rotation speed using the formula:

rotation speed=360(1/burst interval).

In this way, an interval of 2 s would provide an estimate of rotation speed at 180°/s, which was the actual speed of rotation in Experiment 2 (because 2 s was the time taken to complete one rotation). We then ranked converted interburst intervals and for each cell we fitted an exponential function to the values for all sessions combined:

$$rotation\;speed=ae^{b}x,$$

where x is the interburst interval rank position (MATLAB function *fit*, option “exp1” with starting points 100 and 0.25 for a and b, respectively). We included only the first four interburst intervals because cells rarely continued bursting more than four times (Extended Data Fig. 6-1); thus, the number of valid data points is reduced from that point forward. We next calculated the time taken for this exponential function to decay to 1/e or 36.79% of the original 180°/s rotation speed (66.21°/s; MATLAB function *solve*). We repeated the above process for light and dark sessions when animals were head-fixed (Experiment 3) or actively locomoting (Experiment 2). This procedure provided one value for each cell in each session type (i.e., head-fixed visual rotation, head-fixed dark rotation).

10.1523/ENEURO.0174-22.2022.f6-1 Extended Data Figure 6-1 Additional example cells showing postrotational bursting in Experiment 3, where rats were head-fixed and restrained during rotations. Example cells, one per row, left column shows activity in a dark rotation session, right column shows activity for the same cell in a rotation session in the light. Sessions are clipped to the end of the rotation phase (from 5 s before to 15 s after rotations ended). Blue lines denote the animal’s HD, red markers represent action potentials, black areas show a spike histogram (200-ms bins). Blue triangles denote detected spike bursts (Materials and Methods, Spike bursts), blue text between two triangles gives the duration between these bursts. In the dark, cells fired bursts of spikes after the rotations ended. Initial bursts occurred at a frequency close to the rotation frequency, but the time between consecutive bursts increased steadily. In the light, postrotational bursting was absent. Download Figure 6-1, TIF file.

Finally, to calculate an overall vestibular time constant for each session type we averaged the time constants across all cells. For session types other than head-fixed rotations in the dark, interburst intervals either decreased immediately resulting in an extremely small time constant (head-fixed rotations in the light: mean ± SEM = 0.80 ± 0.55 s) or immediately plateaued resulting in a time constant < 0 (actively locomoting rotations in the light: mean ± SEM = −4.43 ± 5.94 s, and dark: mean ± SEM = −1.97 ± 2.09 s); thus, these values were not included in the calculations.

#### Fast Fourier transform

In Experiment 2, HD cells continued to fire in bursts after rotations had ended and these bursts seemed to be at the same frequency as the preceding rotations. To test this observation, for each HD cell, we calculated an instantaneous firing rate vector as the kernel smoothed density estimate of spike times (MATLAB function *fitdist*, 0.02-s bins with a 0.04-s bandwidth Gaussian kernel). We then calculated the frequency-time power spectral density (PSD) spectrogram of this firing rate vector using 512 sample long Hanning windows with 90% overlap and a frequency resolution of 1024 (MATLAB function *spectrogram*). The resulting spectrograms were normalized such that 0 Hz was equal to the frequency of rotation in that session (estimated from the position tracking data) and spectrograms were z-scored column-wise to reduce PSD fluctuations within and across animals.

To compare PSD spectrogram values between visual and dark sessions, we determined the frequency associated with the highest z-scored PSD in each time bin and compared the resulting curves between the two session types. For this analysis we only included frequencies within ±0.2 Hz of the rotation frequency.

#### Histology

At the end of the experiment, rats were anesthetized and a small anodal current (10–20 μA for 10 s) was passed through one of the recording wires to conduct a Prussian blue reaction. Each rat was then perfused transcardially with saline followed by 10% formalin (in saline) and its brain was removed for analysis. Brains were further soaked in 10% formalin and then soaked in 2% potassium ferrocyanide (in 10% formalin). They were then soaked in 10% formalin again and then soaked in 20% sucrose for at least 24 h. The brains were frozen and then sliced on a cryostat (40-μm sections; Hacker-Bright) and mounted onto slides, which after drying were stained with cresyl violet (Nissl stain; Taube et al., 1990b). Analysis indicated that all electrodes passed through the ADN (Extended Data Fig. 1-6).

### Data availability

A summary dataset is available for download (Grieves et al., 2022). The full raw dataset is available from the authors on request.

### Code availability

MATLAB code is available for download which, together with the summary dataset, can be used to regenerate all of the figures and analyses reported in the main text (Grieves et al., 2022). Code was written and run in MATLAB R2021a on a Windows 10, Dell Precision 5820 desktop PC.

## Results

Across three experiments, we recorded ADN HD cell activity in female Long–Evans rats under conditions associated with disorientation (Semenov and Bures, 1989; Dudchenko et al., 1997; Martin et al., 1997). We used a range of rotation speeds equal to or lower than those experienced during natural exploration, but high enough to be detected by the vestibular system. We performed these rotations in the light with visual cues available or blindfolded the rats and performed them in the dark. We also recorded activity while rats were passively restrained or freely moving and during unidirectional and bidirectional rotations. Here, we first outline the general protocols we used and then describe how HD cells responded in each individual experiment. For the main analyses we describe the impact of disorientation on HD cells after normalizing and combining the data from all three experiments. In the final sections, we highlight specific phenomena that were associated with disorientation.

### Head direction cells were recorded across three experiments involving disorienting rotations

In Experiment 1, to record baseline HD cell activity, rats (*n* = 6) first foraged for small sugar pellets in a cylinder in the light for 8 min and then again while blindfolded in the dark for 4 min. For subsequent rotation sessions, rats were rotated unrestrained on a circular platform at either slow or fast speeds. Rotations were made in alternating back-and-forth directions almost continuously for 2 min with each block of rotations lasting 10–30 s before stopping and switching directions. Two test sessions were conducted in darkness while the rat was blindfolded. For a subset of recordings two additional test sessions were conducted in the light with no blindfold (Fig. 1*A*; Materials and Methods, Experiment 1). A summary of the sessions recorded for each rat and cell are shown in Extended Data Figure 1-1*A*. For each dark/light condition the first session consisted of slow back-and-forth rotations (mean rotation speed ± SEM = 111.2 ± 9.4°/s), while the second session consisted of a higher speed rotation (mean rotation speed ± SEM = 186.7 ± 5.8°/s; Extended Data Fig. 1-2*A*). Because the rotation speed and direction were frequently changing, rats experienced angular acceleration throughout the sessions (Extended Data Fig. 1-2*A*). Across six rats, we recorded a total of 25 ADN HD cells that demonstrated significant directional modulation (Fig. 1*B*; all baseline tuning curves are shown in Extended Data Fig. 1-3; representative histology is shown in Extended Data Fig. 1-6*A*). Some sessions suffered from missing tracking data and were excluded from directional analyses (see Materials and Methods, Data preparation).

10.1523/ENEURO.0174-22.2022.f1-2 Extended Data Figure 1-2 In all plots, the data for dark rotation sessions and visual rotation sessions were averaged to give a single value for each session type. ***A***, Left, The AHV of the animal in different sessions and experiment phases. Filled markers represent sessions, lines and circular markers denote mean and SD. AHV was consistent in the dark/light slow/medium rotation sessions. Right, Head direction and AHV throughout an example rotation session. ***B***, Left, Same as ***A***. AHV was consistent in the dark/light rotation sessions. Right, Mean AHV for each session around the end of rotations. Text gives the 2-s impulse: the change in AHV between *t* = −1 and *t* = +1 divided by the change in AHV between *t* = −20 and *t* = +20. The higher this value, the faster AHV decreased after rotations ended. ***C***, Same as ***B*** except for Experiment 3. AHV was very consistent in the dark/light rotation sessions. The 2-s impulse indicates that AHV decreased immediately at the end of rotations. Download Figure 1-2, TIF file.

**Figure 3.**
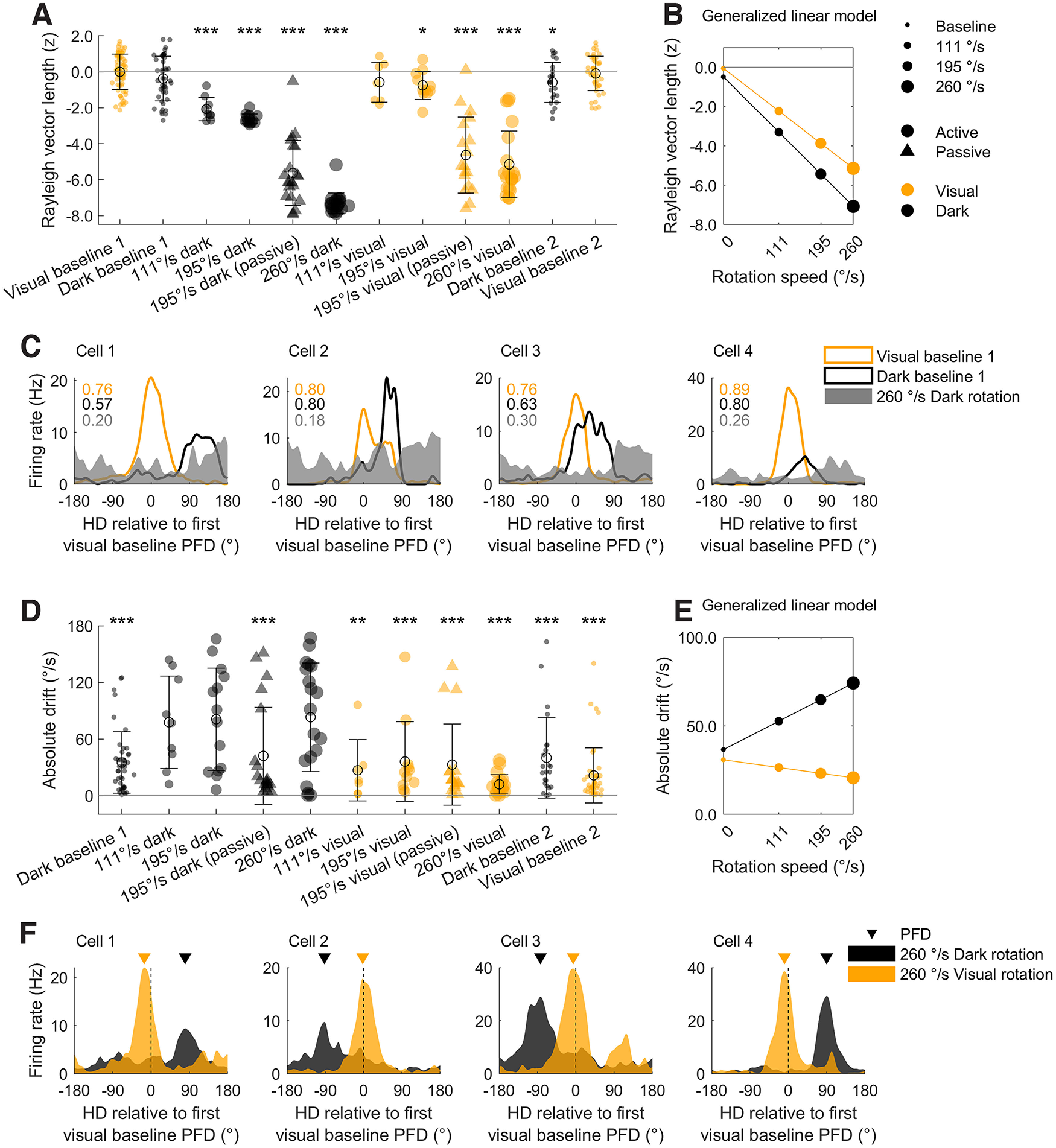
Directionality and stability decreased during rotations. For panels ***A*** and ***D***, group *n* = 71, 48, 25, 25, 23, 23, 25, 25, 23, 23, 25, 48. ***A***, Directionality (Rayleigh vector length) for all HD cells in every experimental condition (see legend on far right). Values are z-scored relative to the first visual baseline session in each experiment. Zero denotes the mean baseline value and values less than zero indicate a decrease from baseline. Top text shows the result of Holm–Bonferroni corrected *t* tests comparing each group to the first visual baseline session (*p < 0.05, **p < 0.01, ***p < 0.001). ***B***, The linear relationship between rotation speed, darkness, and directionality (for a hypothetical animal that is actively locomoting and rotating unidirectionally), extracted from the GLM fitted to the data in ***A***. ***C***, Tuning curves for four HD cells that showed decreased directionality in the fastest rotation condition (260°/s) compared with the baseline sessions. PFD = preferred firing direction. ***D***, Same as ***A*** but for absolute drift: the absolute difference in angle between a cell’s PFD and its PFD in the first visual baseline. Text gives the result of Holm–Bonferroni corrected V-tests for nonuniformity around a mean direction of 0°, a significant value here denotes clustering around 0° and thus a stable PFD. For all three experiments, raw drift values, and their development within sessions, can be seen in Extended Data Figures 3-1 and 3-2. ***E***, Same as ***B*** but for absolute drift. ***F***, Tuning curves for four HD cells during fast rotations (260°/s) in the light and dark. The *x*-axis is normalized so that an angle of zero denotes the cell’s PFD in the first visual baseline. In the light, cells maintain a PFD close to their baseline angle, whereas in the dark, their PFDs drift more readily. In Experiment 2, the angle between co-recorded tuning curves was generally consistent, suggesting that HD cells maintained coherence (Extended Data Fig. 3-3).

10.1523/ENEURO.0174-22.2022.f1-3 Extended Data Figure 1-3 Tuning curves for all HD cells recorded in Experiment 1. Yellow areas show the tuning curve for the first visual baseline session, black areas show the tuning curve for the first dark baseline session, if one was recorded. For all cells, cluster stability was confirmed in a subsequent baseline session. Top right-hand text with corresponding color shows the Rayleigh vector length. Download Figure 1-3, TIF file.

In Experiment 2, rats (*n* = 3) first foraged for sugar pellets in baseline sessions, as in Experiment 1 above, except they were conducted on a circular platform. Sessions took place in the light (8 min) and then again while the rats were blindfolded and in the dark (4 min). In test sessions, rats were rotated unrestrained for 1 min followed immediately by a 2-min session of free exploration on the same platform. These sessions were first conducted in darkness while the rat was blindfolded, after which the sessions were repeated in the light and with no blindfold (Fig. 1*C*; Materials and Methods, Experiment 2). A summary breakdown of the sessions recorded for each rat and cell are shown in Extended Data Figure 1-1*B*. Rotation speed was kept constant and as close to 260°/s as possible (mean visual rotation speed ± SEM = 268.5 ± 9.3°/s, mean blindfolded rotation speed ± SEM = 260.2 ± 9.7°/s), so that the rats did not experience angular acceleration except for the rotation start and end (Extended Data Fig. 1-2*B*). During rotation, rats usually remained motionless along the side of the platform until termination of the rotation, which occurred abruptly. After rotations ended, rats eventually began to move around and move their heads. In the 30 s after rotations ended, rats often showed a bias for moving in the direction of rotation (71.2% of blindfolded sessions, chance = 48.0%, *p *=* *2.1 × 10^−5^ and 63.9% of visual sessions, chance = 48.3%, *p *=* *0.0029; Materials and Methods, Postrotation behavior bias), presumably because cessation of prolonged rotation of the body elicited an illusion of self-motion in the opposite direction, which the rats may have attempted to compensate for (38). Across three rats we recorded 23 HD cells from the ADN (Fig. 1*D*; all baseline tuning curves are shown in Extended Data Fig. 1-4; representative histology is shown in Extended Data Fig. 1-6*B*).

**Figure 4.**
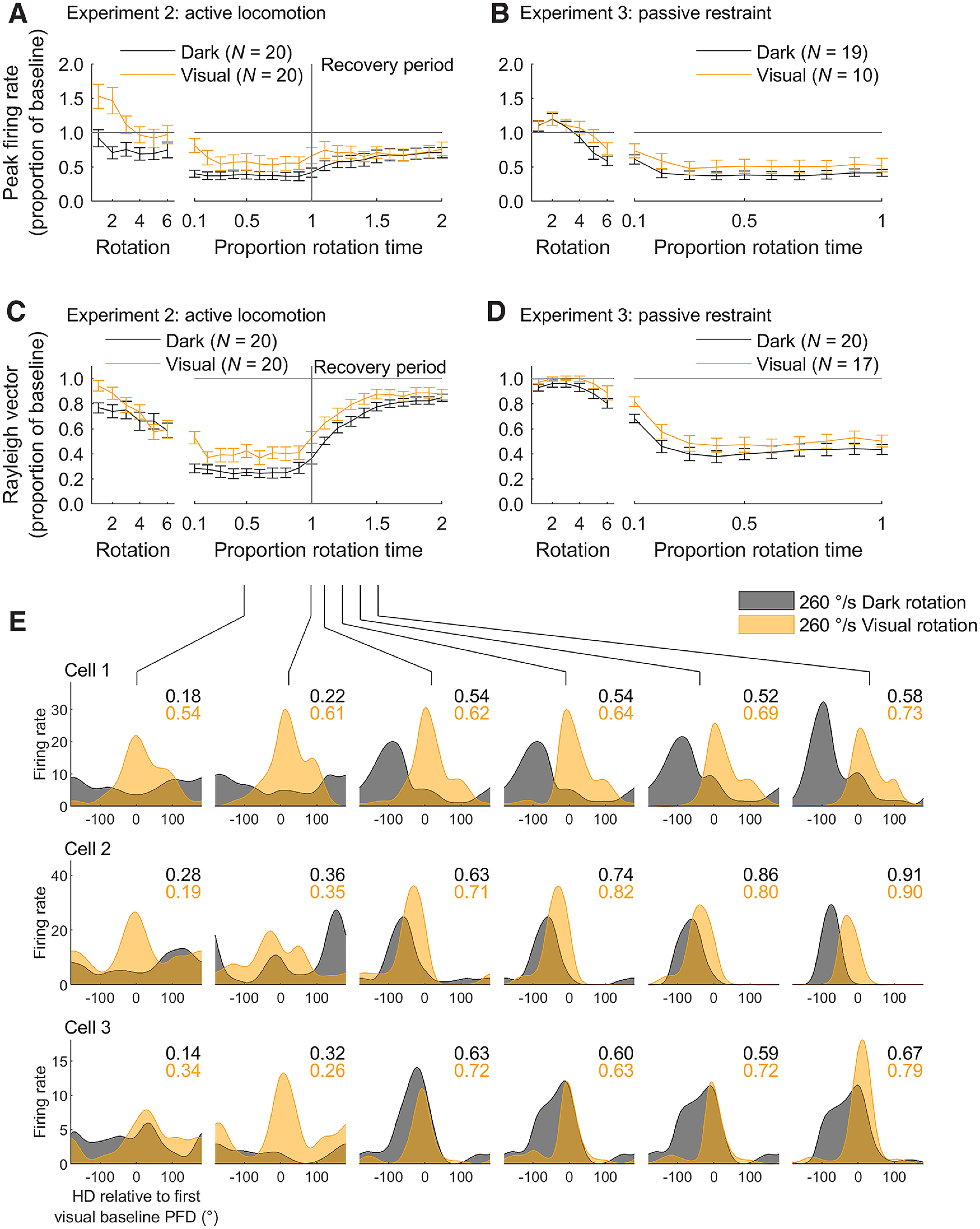
Peak firing rates and directionality recovered after rotations. *A*, Mean ± SEM peak firing rates in Experiment 2 (active locomotion) throughout rotations, expressed as a proportion of the peak firing rate observed in the first visual baseline session. Left axis shows values for the first six rotations. Right axis shows values for the remaining session (10% of rotation period onwards) calculated using a sliding window (see Materials and Methods, Windowed tuning curves and parameters). The total length of the rotation period was 1 min. When a cell was recorded in multiple sessions, the responses were averaged to provide one value per cell. ***B***, Same as ***A*** but for Experiment 3 (passive restraint). The total length of the rotation period was also 1 min. ***C***, ***D***, Same as ***A***, ***B*** but for directionality. ***E***, Tuning curves for three example HD cells, one per row, in the fastest rotation condition (260°/s), in the light and dark. PFD = preferred firing direction. Tuning curves were calculated for nonoverlapping 10-s windows at selected time points throughout the rotation period. Values shown in the upper right corner of each plot are the Rayleigh vector length of the tuning curve with the corresponding color. Directionality is generally higher in the visual rotation sessions but following rotation directionality recovers at a similar speed in light and dark sessions.

10.1523/ENEURO.0174-22.2022.f1-4 Extended Data Figure 1-4 Tuning curves for all HD cells recorded in Experiment 2. Yellow areas show the tuning curve for the first visual baseline session, black areas show the tuning curve for the first dark baseline session, if one was recorded. For all cells, cluster stability was confirmed in a subsequent baseline session. Top right-hand text with corresponding color shows the Rayleigh vector length. Download Figure 1-4, TIF file.

In Experiment 3, rats (*n* = 6) first foraged for sugar pellets in the cylinder and in the light for 8 min. The rats were then restrained in a Plexiglass tube, head-fixed via an implanted bolt on top of their skull, and passively rotated continuously for 1 min via a small, motorized turntable positioned inside the cylinder. They were rotated either in the light or in darkness while blindfolded (Fig. 1*E*; Materials and Methods, Experiment 2). A summary breakdown of the sessions recorded for each rat and cell are shown in Extended Data Figure 1-1*C*. With this setup we were able to limit any sensory/motor input because of locomotion (except for air currents that arose from the motion), neck-on-body proprioception, or voluntary head movements. Additionally, because the turntable was motorized, we were able to keep the rotation speed relatively constant at ∼186°/s (mean rotation speed ± SEM for visual rotations = 186.6 ± 1.1°/s, mean rotation speed ± SEM for dark rotations = 186.4 ± 1.4°/s); thus, the rats did not experience any angular acceleration except for the rotation start and end (Extended Data Fig. 1-2*C*). In this experiment, we recorded 23 HD cells from the ADN (Fig. 1*F*; all baseline tuning curves are shown in Extended Data Fig. 1-5; representative histology is shown in Extended Data Fig. 1-6*C*).

**Figure 5.**
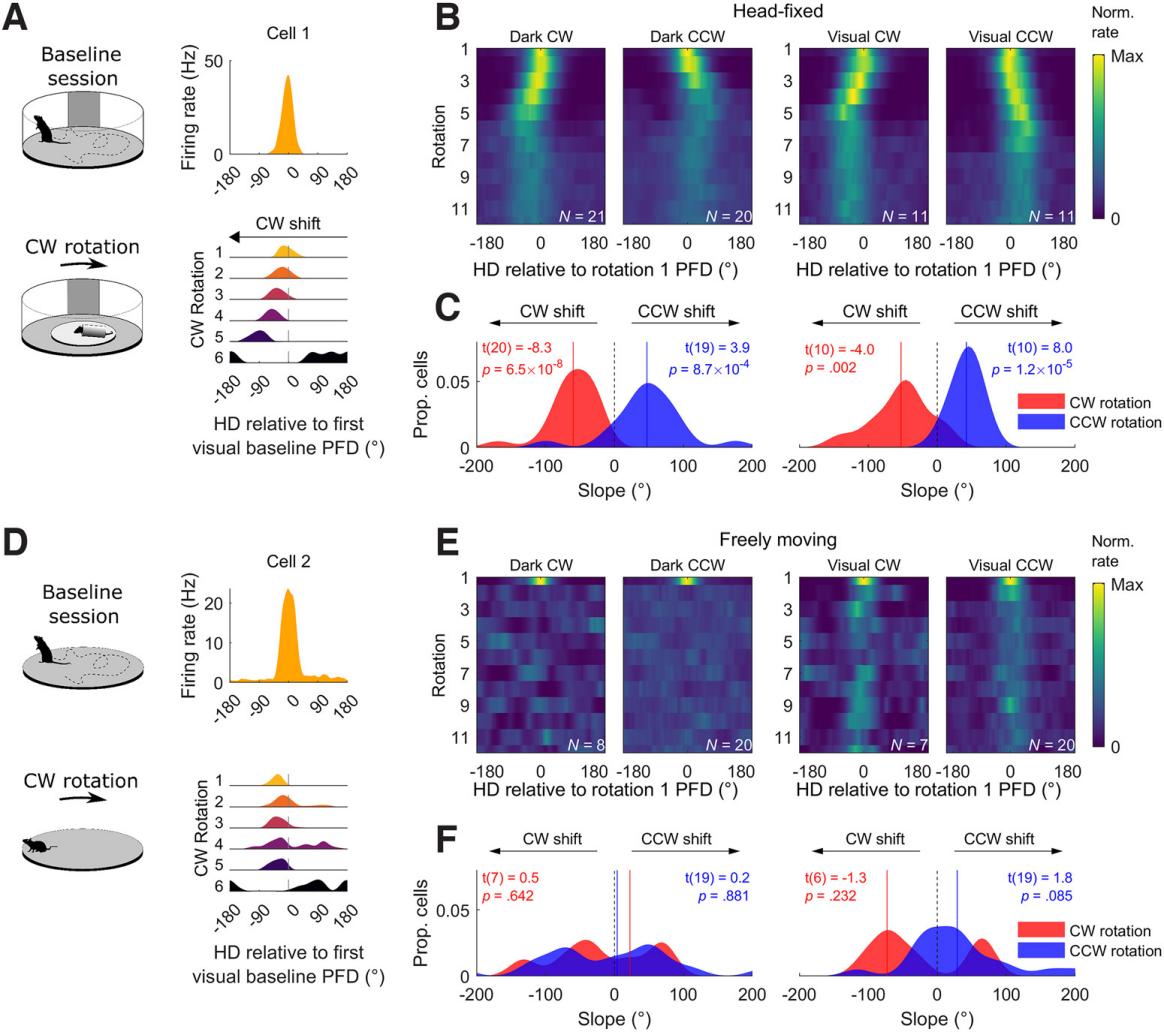
HD cells underestimated angular head velocity in head-fixed sessions. ***A***, Top, Example tuning curve for the first visual baseline. Bottom, Tuning curves for the same cell during the first six rotations in a dark CW rotation session. Angles are normalized such that 0° is the PFD in the first baseline session. PFDs shift in the direction of rotation (leftward for CW). ***B***, Sum normalized tuning curves for the first 12 rotations for all cells (averaged across sessions then cells). Angles are normalized such that 0° is the PFD in the first rotation. ***C***, Circular-linear regression slopes fitted to the PFDs in the first six rotations for all cells. Left plot shows results for dark sessions, right plot shows results for visual sessions. Slopes were negative for CW rotations and positive for CCW rotations indicating that PFDs shifted in the direction of rotation. Vertical colored lines denote distribution means, statistical values show the result of a one-sample *t* test comparing the group mean to zero. ***D–F***, Same as ***A–C*** but for sessions where animals were actively locomoting. Note that the tuning curve shift is absent for these sessions.

10.1523/ENEURO.0174-22.2022.f1-5 Extended Data Figure 1-5 Tuning curves for all HD cells recorded in Experiment 3. Yellow areas show the tuning curve for the first visual baseline session. Top right-hand text shows the Rayleigh vector length. One cell is missing, but this cell was verified as a HD cell by the experimenter before recording. Download Figure 1-5, TIF file.

**Figure 6.**
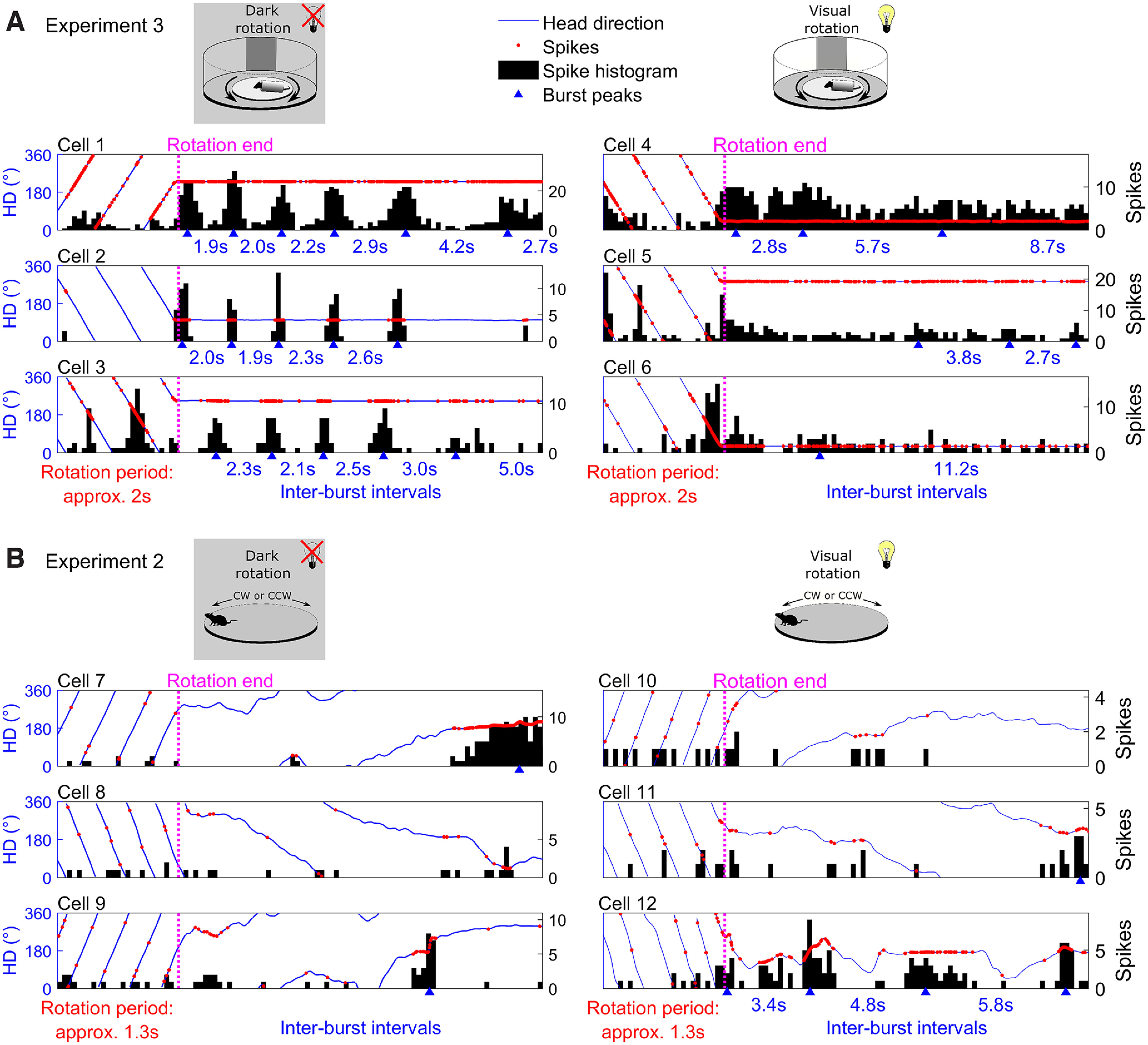
Head direction (HD) cells exhibited postrotational bursting in head-fixed sessions. ***A***, Six example cells, three recorded during head-fixed rotations in the dark (left column), and three recorded in the light (right column). Plots are clipped to the end of the rotation phase (from 5 s before to 15 s after rotations ended), black lines denote the animal’s HD, red markers represent action potentials, black shaded areas show spike count histograms (200-ms bins). Blue triangles denote detected spike bursts (Materials and Methods, Spike bursts), blue text between two triangles gives the duration between these bursts. In the dark, cells fired bursts of spikes after the rotations ended, initial bursts occurred at a frequency close to the rotation frequency, but the time between consecutive bursts increased steadily. In the light this postrotational bursting was absent. See Extended Data Figure 6-1 for further example cells. ***B***, Same as ***A*** but for Experiment 2, where rats could actively locomote during rotations. Postrotational bursting was not observed in the light or dark sessions. See Extended Data Figure 6-2 for further example cells. An additional analysis using the Fast Fourier transform method can also be seen in Extended Data Figure 6-3.

10.1523/ENEURO.0174-22.2022.f1-6 Extended Data Figure 1-6 Representative histology for Experiments 1–3. ***A***, Representative Nissl-stained histology slide for an animal implanted in Experiment 1 with electrode track labelled (black arrows). To the right is a schematic diagram showing delineated brain structures (Paxinos and Watson, 2006) with the anterodorsal thalamic nucleus (ADN) shaded in blue and the reconstructed electrode track shown as a black arrow. ***B***, ***C***, Same as ***A*** but for Experiments 2 and 3. Download Figure 1-6, TIF file.

### Increasing rotation speed decreased peak firing rate and spike bursting

A number of previous studies comparing active and passive movement have reported significant suppression of the HD signal during passive restraint (Knierim et al., 1995; Taube, 1995). However, in each of these studies there was considerable variability across cells and the animals were not head-fixed. In contrast, HD cells responded normally when head-fixed rats were passively rotated back-and-forth for short intervals (Shinder and Taube, 2011). Consistent with these results, we found that mean firing rates (total spikes/total time) did not differ between any of the conditions across our three experiments (Experiments 1–3: *F*_(5,82)_ = 1.4, *p *=* *0.24, ${\text{η}}^{2}$ = 0.08; *F*_(4,100)_ = 1.1, *p *=* *0.38, ${\text{η}}^{2}$ = 0.04; *F*_(2,58)_ = 1.4, *p *=* *0.25, ${\text{η}}^{2}$ = 0.046; one-way ANOVAs), suggesting that activity is maintained in the HD system even during disorientation.

Next, we compared spiking activity measures that were related to directionality, such as peak firing rate, burst index, Rayleigh vector length, and tuning stability. To compare these features across the three experiments, we normalized all values by z-scoring them against values from the first visual baseline sessions (Materials and Methods, Parameter normalization). We first analyzed peak firing rates and burst index scores (the extent to which a cell fires in a burst mode; see Materials and Methods, Burst index); these normalized values are shown in Figure 2*A*,*D*; raw values and comparisons within experiments are shown in Extended Data Figure 2-1*A–D*. Comparing each experimental group to the first visual baseline session using Holm–Bonferroni corrected *t* tests, we found that peak firing rates and burst index scores did not differ between the baseline sessions, regardless of illumination or if they were conducted after the disorienting sessions (Fig. 2*A*,*D*). This result confirms that visual cues are not necessary to drive HD cell activity and that HD cells were recorded stably throughout the sessions.

10.1523/ENEURO.0174-22.2022.f2-2 Extended Data Figure 2-2 ***A***, Head direction (HD) tuning curve and angular head velocity (AHV) tuning curve for an example HD cell in visual baseline session 1. ***B***, Left, the HD × AHV spike maps for the same cell but for dark baseline session 1. Right, The HD × AHV spike tuning histogram predicted for this this cell in dark baseline session 1 based on its activity in visual baseline session 1 (shown in ***A***) and the animal’s AHV × HD sampling in dark baseline session 1. Bins that are empty in either map are shown as empty in both maps. ***C***, Left, The HD tuning curve observed in dark baseline session 1. Right, The HD tuning curve predicted for dark baseline session 1. ***D***, Spike rate index: (a − b)/(a + b), where a is the sum of the actual spike map and b is the sum of the predicted spike map, for every experimental condition. Low values indicate that fewer spikes were recorded than predicted while high values indicate the reverse. Values of zero indicate that a cell fired at exactly the rate predicted, which was the case in almost every condition. Text gives the result of Holm–Bonferroni corrected *t* tests comparing each group to the first visual baseline session (n.s. = *p *> 0.05, *p < 0.05, **p < 0.01, ***p < 0.001). ***E***, Peak rate index: (a − b)/(a + b), where a is the peak firing rate in the actual HD tuning curve and b is the peak firing rate in the predicted HD tuning curve, for every experimental condition. Peak rate indices were generally less than zero, meaning that peak firing rates were lower than would be expected even when AHV tuning was taken into account. Text gives the result of Holm–Bonferroni corrected *t* tests as in ***D***. Download Figure 2-2, TIF file.

Peak firing rates, however, were reduced significantly during dark rotation and fast visual rotation sessions (Fig. 2*A*). By fitting a GLM [Materials and Methods, Generalized linear model (GLM)] across all of the experimental data (*F*_(5,295)_ = 13.5, *p *=* *7.5 × 10^−12^, *R*^2^ = 0.19), we found that faster rotation speed (*β* = −0.35, *t* = −5.15, *p *=* *4.6 × 10^−7^) and, to a lesser extent, darkness (*β* = −0.25, *t* = −4.3, *p *=* *2.3 × 10^−5^) were associated with lower peak firing rates. Although ADN HD cells are known to be modulated by angular head velocity (AHV; Taube, 1995; Taube and Muller, 1998; Shinder and Taube, 2011), this effect could not be explained by AHV modulation (Extended Data Fig. 2-2). Passive restraint (*β* = −0.04, *t* = −0.62, *p *=* *0.53) and unidirectional rotation (*β* = −0.11, *t* = −1.86, *p *=* *0.0636) did not have a significant impact on peak firing rates. Further, rotation speed and darkness did not interact to effect peak firing rates (*β* = −0.05, *t* = −0.82, *p *=* *0.41; all standardized *β* coefficients). This relationship can be visualized in Figure 2*B* and example cells are shown in Figure 2*C*.

Burst index scores decreased significantly in all rotation sessions, indicating that cells fired less often in a bursting mode during rotations compared with active baseline sessions. This decrease was larger in sessions with faster rotation speeds (Fig. 2*D*). A GLM fit to all of the experimental data (*F*_(5,336)_ = 96.1, *p *=* *1.22 × 10^−62^, *R*^2^ = 0.59) revealed that, in order of decreasing magnitude, faster rotation speed (*β* = −1.27, *t* = −16.3, *p *=* *2.1 × 10^−44^), passive rotation (*β* = −0.39, *t* = −5.2, *p *=* *4.5 × 10^−7^) and unidirectional rotation (*β* = −0.28, *t* = −3.7, *p *=* *2.2 × 10^−4^) were all associated with decreased burst index scores. Darkness did not have a significant impact on burst index scores alone (*β* = −0.02, *t* = −0.34, *p *=* *0.73) or in interaction with rotation speed (*β* = −0.07, *t* = −1.1, *p *=* *0.28; all standardized *β* coefficients). This relationship can be visualized in Figure 2*E* and example cells are shown in Figure 2*F*.

### Visual cues greatly increase stability but only mildly increase directionality during rotation

To compare the strength of the directional signal (as measured by the Rayleigh mean vector length and hereafter referred to as directionality) across the three experiments, we normalized mean vector length values using the same approach as described above (Fig. 3*A*; Materials and Methods, Parameter normalization). We also calculated the stability of HD cells by measuring the absolute angle between each session’s preferred firing direction (PFD; HD of peak firing) and the cell’s PFD in the first visual baseline session (Fig. 3*D*). PFD stability across rotations and comparisons within experiments are shown in Extended Data Figure 3-1; fine temporal scale examples are shown in Extended Data Figure 3-2. Directionality and stability were high in all baseline sessions, including those in the dark (Fig. 3*A*,*D*). This result confirms that visual cues are not necessary for maintaining HD cell directionality or stability and that HD cell firing remained stable throughout the recording sessions.

10.1523/ENEURO.0174-22.2022.f3-1 Extended Data Figure 3-1 PFD drift throughout baseline and rotation sessions. In all plots, filled markers represent HD cells. For Experiments 2 and 3, when a cell was recorded in one session type more than once, their responses were averaged to give a single value for each session type. Asterisks above each plot denote a significant mean direction around 0° (PFDs were consistent with visual baseline 1; tested using Holm–Bonferroni corrected v-tests), color corresponds to group color (see legends). The *x*-axes for Experiment 3 are truncated shortly after the end of the rotation period as the animal could no longer sample directional angles. Download Figure 3-1, TIF file.

10.1523/ENEURO.0174-22.2022.f3-3 Extended Data Figure 3-3 ***A***, A pair of co-recorded HD cells in the first visual baseline session and subsequent blindfolded baseline session. ***B***, In both sessions, the tuning curve of cell 2 is rotated to find the angular shift at which it most highly correlates with cell 1. These angles are similar and deviate by only 7°, confirming that the cells remain coherent between sessions. ***C***, Results when the same procedure is conducted on all six pairs of co-recorded HD cells in Experiment 2. Filled markers represent cell pairs, lines and circular markers denote circular mean and SD. The angle between tuning curves in each session is compared to the angle observed in the first visual baseline. The four blindfold rotation sessions and two visual rotation sessions are averaged to give a single value for each session type. The text above each group gives the result of a v-test of nonuniformity around 0°; significance here denotes a bias for values around 0°. Cell pairs were significantly coherent in all sessions, even during rotation, except when the rat was blindfolded and rotated. However, this group also exhibits a mean direction of 0° and is nearing significant directionality for this angle. Download Figure 3-3, TIF file.

10.1523/ENEURO.0174-22.2022.f3-2 Extended Data Figure 3-2 Example HD cells showing the impact of disorientation on HD cell tuning. ***A***, Left, Schematics representing the session types in Experiment 2, when rats were rotated unrestrained. Sessions are arranged temporally from top to bottom. Right, Two example HD cells, one per column. Tuning curves are given for baseline sessions (first and last rows). Windowed tuning curves (see Materials and Methods, Windowed tuning curves and parameters) are shown for rotation sessions (middle rows). Black horizontal arrows denote the end of the rotation period and the start of the recovery period. During dark rotations HD cells were disrupted, they lost directionality and their tuning curves were unstable. However, in the recovery period cells quickly regained these properties, even in the absence of visual cues, although their PFDs were offset randomly from baseline. During rotations in the light, cells remained largely unaffected throughout the rotation. ***B***, Same as ***A*** but for Experiment 3 when rats were rotated while restrained. Here, directionality decreased quickly during rotation sessions, but cells maintained roughly consistent PFDs. Tuning curves also drifted in the direction of rotation at the start of rotation sessions. Download Figure 3-2, TIF file.

Directionality was significantly decreased in all rotation sessions however, except for the slowest visual rotation sessions (Fig. 3*A*). Fitting a GLM to all of the experimental data (*F*_(5,295)_ = 226.01, *p *=* *1.24 × 10^−98^, *R*^2^ = 0.79) revealed that, in decreasing order of magnitude, faster rotation speeds (*β* = −2.32, *t* = −26.6, *p *=* *2.94 × 10^−80^), unidirectional rotation (*β* = −0.81, *t* = −10.1, *p *=* *1.2 × 10^−20^) and darkness (*β* = −0.45, *t* = −6.2, *p *=* *2.4 × 10^−9^) all significantly reduced directionality. There was also a significant interaction between rotation speed and darkness (*β* = −0.30, *t* = −4.1, *p *=* *4.6 × 10^−5^) such that directionality was disrupted more by increasing rotation speed in darkness than in the light. This relationship is depicted in Figure 3*B*; example cells are shown in Figure 3*C*. Passive restraint also reduced mean vector length, but this reduction did not reach significance (*β* = −0.15, *t* = −1.85, *p *=* *0.0658; all standardized *β* coefficients).

PFDs were stable relative to the first visual baseline in all sessions except nonhead-fixed dark rotation sessions and this instability appeared to increase with rotation speed (Fig. 3*D*; Extended Data Fig. 3-1). In agreement with this observation, fitting a GLM to all of the experimental data (*F*_(5,283)_ = 10.2, *p *=* *5.2 × 10^−9^, *R*^2^ = 0.15) revealed a significant interaction between darkness and rotation speed; increasing rotation speed in the dark was associated with significantly greater drift in the cell’s PFD (*β* = 9.6, *t *=* *4.0, *p* = 7.8 × 10^−5^). Darkness alone was also associated with increased PFD drift (*β* = 10.4, *t *=* *4.3, *p* = 2.3 × 10^−5^). No other factors significantly affected drift (rotation speed: *β* = 4.4, *t *=* *1.5, *p *=* *0.13; unidirectional rotation: *β* = −5.1, *t* = −1.9, *p *=* *0.0580; passive restraint: *β* = −2.64, *t* = −0.96, *p *=* *0.34). This relationship is shown in Figure 3*E*, example cells are shown in Figure 3*F*. Consistent with attractor network dynamics, co-recorded HD cell pairs remained coherent in terms of their PFDs throughout Experiment 2, except in dark rotation sessions, where they were marginally incoherent (*p *=* *0.0575; Extended Data Fig. 3-3). This decrease in coherence, however, could be explained by PFD drift, which was observed during rotations.

### Visual cues do not accelerate recovery from disorientation

As peak firing rates (Fig. 2*B*) and directionality (Fig. 3*B*) both decreased significantly during rotations we next sought to determine, on a finer timescale, how this decrease initially took place and how cells recovered when rotations ended. Regardless of whether rats were able to actively locomote or were passively restrained, peak firing rates decreased from baseline levels very quickly after rotation onset (Fig. 4*A*,*B*). In Experiment 2, after rotations were ended abruptly rats were free to self-locomote during a recovery period. During this time, firing rates remained suppressed for the remainder of the session (2 min), despite the presence of visual landmark cues and the ability to move around freely (Fig. 4*A*; only the first 1 min of recovery is shown).

As with peak firing rates, directionality decreased very quickly from baseline levels after rotation onset regardless of self-locomotion (Fig. 4*C*,*D*). In the postrotation recovery period of Experiment 2, directionality increased gradually to near baseline levels but, surprisingly, the speed of this recovery was the same in the light and in dark sessions (Fig. 4*C*; visual and dark mean slopes in first half of recovery period: 0.51 and 0.57, *t*_(19)_ = 0.33, *p *=* *0.745, Cohen’s *d *=* *0.09), although mean vector lengths were consistently lower in the dark sessions (visual and dark mean y-intercept in first half of rotation: 0.46 and 0.34, *t*_(19)_ = −2.10, *p *=* *0.049, Cohen’s *d *=* *0.25). Together these results suggest that while visual cues enable HD cells to sustain greater directionality (signal strength) during disorientation, they do not facilitate a faster recovery from disorientation. Example tuning curves at different periods of recovery from disorientation are shown in Figure 4*E* and Extended Data Figure 3-2.

### The HD system underestimated angular head velocity

When rats were restrained, HD cells maintained their PFDs during rotations even while their overall directionality (signal strength) decreased, suggesting that they may have used AHV to maintain a consistent PFD [Fig. 3*D*, 195°/s (passive) groups]. However, PFDs were not completely stable and tended to shift later in the rotation phase, effectively firing at a later HD than expected (Fig. 5*A*, diagonal drift of tuning curves).

To investigate this result further we generated tuning curves for each rotation and calculated the PFD for each rotation separately (Fig. 5*A*,*B*). Because directional signal strength and firing rates decreased significantly after 6–12 rotations (Fig. 4*A–D*), we focused on only the first six rotations, after which the cell’s directional signal strength was diminished to the point that the cell’s mean vector length had decreased to <0.5. We then used circular-linear regression to determine the rate at which the PFDs shifted (i.e., slope of the cell’s PFD over time). If PFDs shifted consistently in the direction of rotation, we would expect positive slopes for CCW shifts during CCW rotations and negative slopes for CW shifts during CW rotations. Our analysis was consistent with these predictions in sessions with passive restraint (Fig. 5*C*). Based on regression slopes, during the first six dark rotations, cells underestimated CW rotation speed by 4.99°/s and CCW rotation speed by 3.92°/s, while during the first six light rotations, cells similarly underestimated CW rotation speed by 4.38°/s and CCW rotation speed by 3.52°/s. The amount of underestimation did not differ between visual and dark sessions (*t*_(19)_ = 0.32, *p *=* *0.7553, Cohen’s *d *=* *0.04; two-sample *t*-test comparing absolute visual/dark slopes). Importantly, however, these effects were not observed in sessions where animals could actively locomote (Fig. 5*D–F*).

### Postrotational bursting was observed only in head-fixed sessions

When rats were restrained and head-fixed during rotations in Experiment 3, peak firing rates were reduced compared with baseline sessions (Fig. 4*B*). However, in dark sessions, HD cells fired three to five bursts of spikes for a few seconds immediately after the rotations were terminated (Fig. 6*A*, left; Extended Data Fig. 6-1, left). These bursts initially occurred at a frequency close to the rotation frequency (0.5 Hz or at a 2-s interval), but the time between consecutive bursts increased steadily postrotation. This relationship was not observed in visual sessions (Fig. 6*A*, right; Extended Data Fig. 6-1, right) or in Experiment 2, where rats could actively locomote during the rotations (Fig. 6*B*; Extended Data Fig. 6-2). By analyzing the rate with which these interburst intervals increased (Materials and Methods, Time constant) we estimated the time constant for the dissipation of vestibular inputs to be 3.81 s (SEM = 0.79 s) in dark sessions where animals were head-fixed. In all other sessions, interburst intervals either decreased or immediately plateaued making a time constant impossible to estimate (values provided in Materials and Methods, Time constant). An alternative analysis, based on the fast Fourier transform of kernel smoothed spike counts, confirmed these results (Extended Data Fig. 6-3).

10.1523/ENEURO.0174-22.2022.f6-2 Extended Data Figure 6-2 Example cells showing an absence of postrotational bursting in Experiment 2, when rats were free to actively locomote during rotations. Example cells, one per row, left column shows activity in an example dark rotation session, right column shows activity for the same cell in an example rotation session in the light. Sessions are clipped to the end of the rotation phase (from 5 s before to 15 s after rotations ended). Blue lines denote the animal’s HD, red markers represent action potentials, black bars show a spike histogram (200-ms bins). Blue triangles denote detected spike bursts (Materials and Methods, Spike bursts), blue text between two triangles indicates the duration between these bursts. In both session types cells generally did not fire in bursts after the rotations ended. The bottom most cell (cell 7) is the only example of potential postrotational bursting that occurred in Experiment 2 during a dark rotation session. However, the number of spikes emitted was so low that these postrotational bursts did not meet our detection criteria. Download Figure 6-2, TIF file.

10.1523/ENEURO.0174-22.2022.f6-3 Extended Data Figure 6-3 Postrotational bursting analyzed by FFT (fast Fourier transform). ***A***, FFT analysis of the kernel smoothed spike counts shown in Figure 6, but for all cells recorded during passive restraint (median across sessions then cells), separated by sessions in the dark (top) and light (middle). These spike counts are normalized such that 0 Hz represents the rotation frequency and time is expressed as a proportion of the rotation duration. High-power regions can be seen at the start of the rotation (*t* = 0), which correspond to the activity shown in Fig. 5. A second high-power region can be seen after the rotations have ended (*t* = 1) corresponding to the postrotation bursts shown in Figure 6*A*, but only for the sessions in darkness. Bottom, For each time point, the frequency associated with the highest power; this frequency decreased immediately at the end of light sessions but remained closer to the rotation frequency in dark sessions. The text shows the between-group difference result of a two-way ANOVA for data between *t* = 1.0 and *t* = 1.2. There was also a significant effect of time (*F*_(18,836)_ = 2.12, *p *=* *4.21 × 10^−3^, ${\text{η}}^{2}$ = 0.04) and a significant interaction between time and group (*F*_(18,836)_ = 6.21, *p *=* *1.76 × 10^−14^, ${\text{η}}^{2}$ = 0.11). ***B***, Same as ***A*** but for cells recorded during rotations where animals were free to actively locomote. Postrotation bursting is absent. Bottom, There was no significant effect of time (*F*_(18,760)_ = 0.47, *p* = 0.9700, ${\text{η}}^{2}$ = 0.01) or interaction between time and group (*F*_(18,760)_ = 0.51, *p* = 0.9554, ${\text{η}}^{2}$ = 0.01). Download Figure 6-3, TIF file.

### Rotation and inversion disrupt HD cells similarly

Finally, we sought to compare the activity of HD cells under two different conditions of disorientation: constant rotation and inversion. The rotation data we used were from Experiment 3 where spike events were accurately timestamped to enable analysis of interspike intervals (ISIs). The inversion data were taken from Shinder and Taube (2019), where rats were head fixed and oriented facing the HD cell’s PFD (among other angles not used here) either upright or upside down. For comparison between these two experiments, we filtered the spikes from Experiment 3 to include only those spikes that occurred when the animal was facing within ±30° of the HD cell’s PFD.

**Figure 7.**
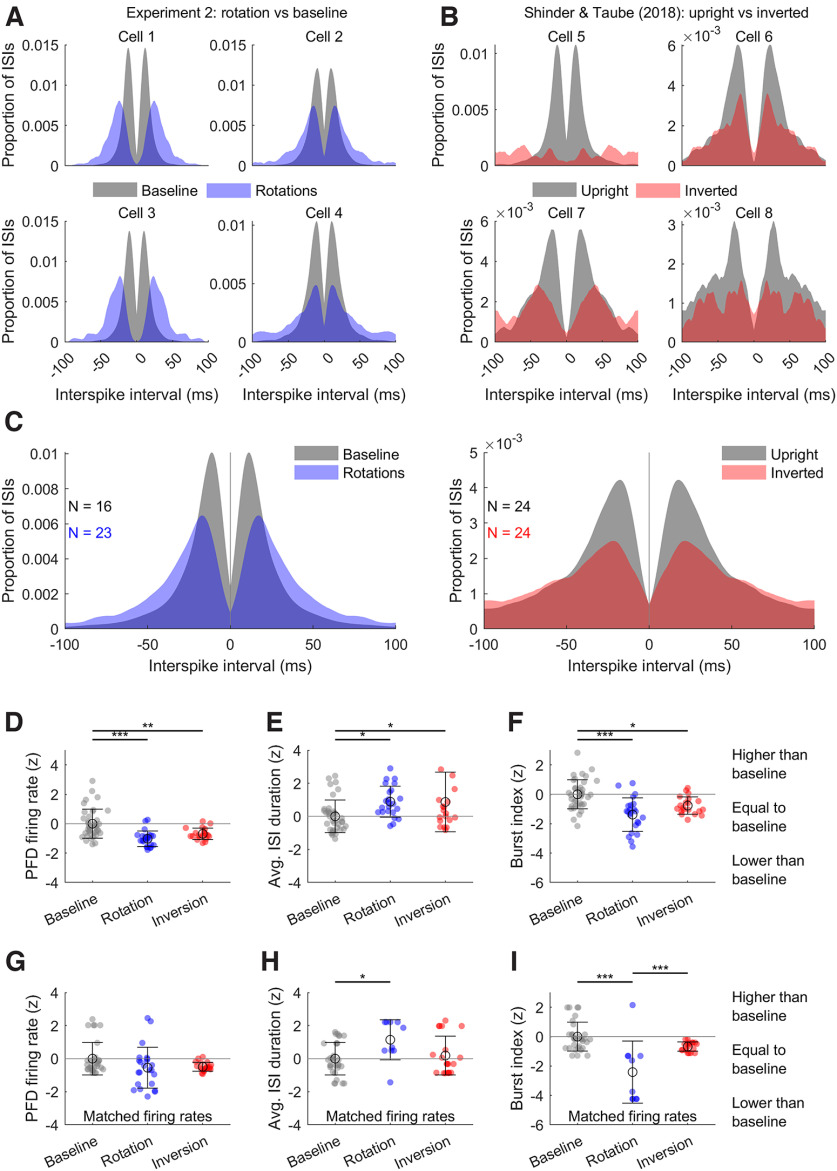
Rotation and inversion share similar effects on HD cells. See Materials and Methods, Rotation and inversion comparisons. ***A***, Histograms showing the distribution of interspike intervals (ISIs) during baseline and rotation sessions in Experiment 3, for four example cells. During baseline sessions rats actively locomoted in an open arena; during rotations rats were head-fixed and restrained. Only spikes emitted within ±30° of the cell’s PFD are included. ***B***, Same as ***A*** but for four example cells recorded while restrained, head-fixed rats faced a HD cell’s PFD for 30 s either upright or inverted (upside down). Data are from Shinder and Taube (Shinder and Taube, 2019). ***C***, Same as ***A***, ***B*** but showing the distribution of interspike intervals (ISIs) averaged across sessions and then cells. ***D–I***, In each case, data were z-scored relative to corresponding baseline values. Colored markers represent cells; when multiple sessions were recorded per cell, values were averaged across sessions. Empty circles denote group mean, error bars denote SEM. Tests are one-way ANOVAs; the statistical significance of *post hoc* tests is shown by horizontal brackets and asterisks above the plots (*p < 0.05, **p < 0.01, ***p < 0.001). ***D***, HD cell PFD firing rates when rats were rotated or inverted. Groups differed significantly (*n* = 39, 23, 20; *F*_(2,79)_ = 14.3, *p *=* *4.9 × 10^−6^, ${\text{η}}^{2}$ = 0.27). ***E***, Same as ***D*** but for average ISI duration. Groups differed significantly (*n* = 39, 23, 20; *F*_(2,79)_ = 5.3, *p *=* *7.0 × 10^−3^, ${\text{η}}^{2}$ = 0.12). ***F***, Same as ***D***, but for burst index; the proportion of ISIs <25 ms. Groups differed significantly (*n* = 39, 23, 20; *F*_(2,79)_ = 15.7, *p *=* *1.9 × 10^−6^, ${\text{η}}^{2}$ = 0.28). ***G–I***, Same as ***D–F***, but groups are matched to baseline sessions in terms of PFD firing rate before normalization. ***H***, Groups differed significantly (*n* = 32, 10, 20; *F*_(2,81)_ = 6.3, *p *=* *2.8 × 10^−3^, ${\text{η}}^{2}$ = 0.38). ***I***, Groups differed significantly (*n* = 32, 10, 20; *F*_(2,81)_ = 24.7, *p *=* *4.3 × 10^−9^, ${\text{η}}^{2}$ = 0.28).

If cells fired with a lower number of bursts during periods of disorientation, then we would expect a decrease in both burst index and the proportion of short ISIs. To explore this possibility, we first plotted the ISIs from Experiment 3 during active locomotion in visual baseline sessions (“baseline”) and during rotation (“rotations”; examples are shown in Fig. 7*A*, averages are shown in Fig. 7*C*, left). We also plotted the ISIs from Shinder and Taube (2019) when rats faced the cell’s PFD in an upright position (“upright”) and when upside down (“inverted”; examples shown in Fig. 7*B*, averages shown in Fig. 7*C*, right). In both experiments, ISI distributions were skewed toward longer durations and an increase in the mean ISI duration confirmed this effect (Fig. 7*E*). Consistent with this result, both constant rotation and inversion conditions were accompanied by a reduction in bursting (Fig. 7*F*).

In both experiments, however, PFD firing rates were also significantly reduced (Fig. 7*D*), which would affect other spiking features. To control for this effect, we matched group PFD firing rates to baseline sessions (Fig. 7*G*; Materials and Methods, Rotation and inversion comparisons) and found that the similarity between mean ISI durations increased (Fig. 7*H*), while the differences in burst index remained largely the same (Fig. 7*I*). These responses likely occurred because we calculated burst index as the proportion of ISIs <25 ms, which should independently control for firing rate differences. Together, these similar effects during rotation and inversion may occur because both inversion and disorienting rotations lead to similar disruptive effects on the underlying neural processes, particularly those receiving inputs from the vestibular system.

## Discussion

We monitored the activity of HD cells in the ADN while rats underwent unidirectional or bidirectional rotations with the purpose of observing their directional responses during and after disorientation. The rotations were at angular speeds equal to or lower than those experienced during natural exploration, for short periods of time, but high enough to be detected by the vestibular system. We performed these rotations in the light with visual cues available or blindfolded the rats and performed them in the dark. We also recorded activity while rats were passively restrained or freely moving. Although we did not confirm behaviorally if rats were disoriented, previous research shows that rotations at similar speeds and durations to those used here elicited strong disorientation in humans (Wang and Spelke, 2000; Sargent et al., 2008) and rats (Semenov and Bures, 1989; Dudchenko et al., 1997; Martin et al., 1997). Furthermore, after rotations had ended in Experiment 2, rats continued moving in the same direction, which is consistent with enduring vestibular disruption (the somatogyral or “turning” illusion; Peters, 1969). Below, we describe the implications of our results.

### Changes in peak firing rate and burst statistics during rotations were similar to those observed when rats are inverted

Initial reports suggested that motor and proprioceptive information both play a significant role in generating and updating the HD signal, which leads to decreased HD cell peak firing rates when animals are passively transported (Taube et al., 1990a; Chen et al., 1994), especially if they are restrained (Knierim et al., 1995; Taube, 1995; Golob et al., 1998). Decreased firing has also been reported in some dorsal tegmental nucleus AHV cells during passive, restrained rotation (Sharp et al., 2001). In contrast, Shinder and Taube (2011) found that HD cell firing rates were not affected by head-fixed or hand-held passive rotations and Keshavarzi et al. (2022) found no change in retrosplenial cortex AHV cells during passive, restrained rotation.

We found that overall mean firing rates did not change, regardless of locomotor, vestibular, or visual inputs, suggesting that HD cell excitation remains generally intact during disorientation. However, peak firing rates decreased as rotation speed or time from rotation onset increased, and this reduction was offset by access to visual cues. While peak firing rates were not significantly lower in dark versus light baseline sessions, fitting a generalized linear model to the data from all three experiments did find that darkness was associated with a mild decrease in peak firing rate, independent of rotation speed. These reduced firing rates could not be fully explained by differences in AHV sampling during the rotation sessions. HD cell activity during rotations shared a number of features with activity observed when rats are inverted, either with (Shinder and Taube, 2019) or without restraint (Calton and Taube, 2005; Gibson et al., 2013) or navigating under simulated 0-g conditions (Taube et al., 2004): (1) in both cases, HD cell peak firing rates and directionality were reduced while background firing rates increased; (2) interspike intervals increased; and (3) cells fired in bursts significantly less often. These results suggest that constant rotation and inversion are accompanied by similar disruption to the HD network, likely because both result from vestibular disruption. In the case of inversion, vestibular disruption comes about because of a drastic change in the otolith signal from normal upright activation, while in the case of constant rotation, vestibular disruption occurs because of the loss of a semi-circular canal signal that cannot keep up with the rotations.

Shinder and Taube (2011) reported that peak firing rates were not reduced during passive rotations, which is different from the current findings. It is possible that this difference occurred because the former study rotated rats in a bidirectional manner and at a variety of angular velocities, while we mainly tested responses during unidirectional rotations with a constant velocity (Experiments 2 and 3). Knierim et al. (1998) reported that in darkness HD cells maintain directional specificity and stability much longer if rotations are bidirectional, presumably because they provide constant vestibular input, which can aid in the spatial updating process. In agreement with this view, we found that directionality and stability (in darkness) were negatively affected by unidirectional rotation once other factors such as rotation speed were removed. However, we also found that directionality and stability were reduced during bidirectional rotations in the dark, suggesting that the additional vestibular cues were not sufficient to fully correct angular drift. Taken together, these results highlight the importance of vestibular input to the HD system (Wallace et al., 2002; Muir et al., 2009).

We described earlier the different types of disorientation that occur and raised the issue of how HD cells might respond during periods of disorientation. While HD cells will respond normally during Type I disorientation because this condition is more characterized as misorientation, normal HD cell firing patterns during Types II and III disorientation are disrupted. HD cells, however, do not become quiescent and they appear to burst less frequently and have higher ISIs than during nondisorientation periods, at least measured by their burst index and ISI histograms. In contrast, overall mean firing rates between baseline and disorientation sessions were similar. Taken together, these results suggest that network properties remain intact during disorientation, and the presumed attractor network is still operational, although the network is not being activated in a manner that it normally would. This result makes sense since our applied rotational manipulations did not interfere with the anatomic connections or cellular firing properties of the network, as a lesion or inactivation manipulation might do. In this way, disorientation brings about a state of neural firing that is different from normal ways of activation. In essence, these abnormal forms of firing represent the neural manifestation of disorientation, and likely lead to the perceived sense of disorientation. One might raise the “chicken and egg” question of what comes first: does a subject’s perceived disorientation come about because they “feel” disoriented and therefore HD cell firing is disrupted, or does the subject experience disorientation because the HD system is firing abnormally? In our view, the latter situation is more likely the case because the non-normal vestibular activation happens first, and this occurrence results in the abnormal HD cell firing. A similar process can be considered to occur during inversion and leads to a similar disruption in normal HD cell firing and the consequential perception of disorientation.

### Visual and vestibular inputs work cumulatively but visual cues alone cannot overcome active vestibular disruption

A GLM fitted to the data from all three experiments highlighted a significant negative effect of darkness on directionality, separate to any effect of rotation speed. However, this effect was mild and ranked as the weakest among the significant coefficients. Furthermore, when we directly compared baseline sessions in the light to baseline sessions in the dark, we did not find a significant difference in directionality. This pattern of results suggests a very mild effect of darkness alone and likely occurs because the dark baseline sessions were quite short (4 min), suggesting that there was little time for the HD network to drift or lose directionality. This result is also consistent with previous reports (Mizumori and Williams, 1993; Knierim et al., 1998), which suggest that HD cells drift by ∼1.5°/min in darkness (Peck and Taube, 2017).

Directionality also decreased as rotation speed increased and this occurrence was exacerbated when visual cues were absent. Similarly, HD cell PFDs were increasingly unstable as rotation speed increased in the dark. Interestingly though, stability was unaffected by the rotations when visual cues were available. Together, these results suggest that visual cues may be used to orient and stabilize the HD signal while vestibular inputs are more crucial for updating the directional tuning over short timescales of a few seconds. They also highlight the distinction between directionality (i.e., Rayleigh vector length) and stability (i.e., absolute PFD drift), low individual directionality scores do not necessarily mean that the HD system is nonfunctional.

Although the degree of directionality and stability remained higher when visual cues were available, they did not facilitate a faster recovery to baseline levels after the rotations ended. This finding suggests that vestibular postrotatory sensations must dissipate following disorientation before normal directionality can return and that visual inputs surprisingly do not speed up this process. At first glance, this finding appears to contradict the stability afforded by visual cues during rotations; however, the vestibular system is insensitive to constant unidirectional rotation (Mach, 1875; Steinhausen, 1933), and thus, visual inputs would not be competing with an active vestibular input during rotations. After a period of disorientation, it may be more beneficial for animals to experience normal vestibular stimulation than stable visual cues. Indeed, Shinder and Taube (2019) reported that after a period of inversion, normal HD cell function only returned after head-restrained rats were passively rotated back-and-forth a few times through the cell’s PFD.

### Limiting self-locomotion increases stability but also reveals a consistent underestimation of rotation angle

Examining HD cell activity more closely during the initial 6–12 rotations, we found strong differences between head-fixed and freely moving sessions. If the rats could actively locomote, HD cells lost their directionality almost immediately during the dark rotations, but remained relatively stable if visual cues were available, consistent with their general activity during rotations as described above. However, during head-fixed sessions cells showed a consistent and characteristic response: with each revolution their tuning curves increasingly shifted in the direction of rotation whether or not visual cues were available. This cumulative shift is consistent with the gradual loss of sensitivity we would expect to occur in the vestibular system under constant unidirectional rotation (Mach, 1875; Steinhausen, 1933). It would also presumably lead to an underestimation of the angle traveled. Indeed, human subjects often underestimate rotation angles after as little as one rotation (Guedry et al., 1971; Loomis et al., 1993; Israël et al., 1996; Marlinsky, 1999; Day and Fitzpatrick, 2005), and the underestimation is often proportional to the magnitude of the rotation, which is consistent with the cumulative shift we observed in HD cell tuning curves.

Why this cumulative drift was only observed during head-fixed rotations and not during head-free rotations is unclear. It is also unclear why cells were generally more stable during head-fixed rotations, even maintaining stable PFDs in the dark, compared with head-free rotations. One possibility is that head movements from active locomotion introduced an additional source of directional noise and complexity to the rotations, which more quickly led to disorientation in freely moving sessions. Another possibility is that self-generated movements inhibited (or suppressed) vestibular signals (Wiener, 1993; Sharp et al., 2001; Cullen, 2014; Cullen and Taube, 2017), which in turn disrupted the shift of the cells’ PFDs and disoriented the animal over time. A third possibility is that head-free rats were rotated “off-center” to the vertical axis of their head and this off-axis rotation introduced additional linear acceleration effects and made deciphering angular estimation more difficult. Taken together, these results highlight the impact of self-motion signals on orientation and suggest that further investigation into the effects of disorientation on the HD network are likely best served by head-fixed experiments.

### Postrotational bursting in HD cells resembles postrotational nystagmus (PRN)

In dark sessions where rats were head-fixed and passively rotated, HD cells fired bursts of spikes immediately after rotations ended. These bursts initially occurred at a frequency matching the rotations, but decreased steadily postrotation (i.e., the time between successive bursts increased). These effects were not observed in light sessions or if rats were free to actively locomote during the postrotation period. This postrotational spike bursting was also noted in an earlier brief report when unrestrained rats were rotated continuously on a turntable for ∼1 min and then stopped abruptly (Yoder and Taube, 2014, see their Fig. 2). By analyzing the rate with which interburst intervals increased in dark, head-fixed rotation sessions, we estimated the time constant for the dissipation of vestibular inputs to be 3.8 s, which is similar to that calculated based on physiological examination of the rat vestibular system (3.7 s; Fischer et al., 1979), but shorter than the duration of nystagmus (described below) commonly reported for rats [180°/s = 6.5 s (Fischer et al., 1979); 240°/s = 5.7 s (Griffith, 1920)].

This phenomenon is reminiscent of postrotation vestibulo-ocular activity, or “postrotational nystagmus”: during rotation the eyes make compensatory smooth eye movements followed by a saccade-like reset phase (when the eye movement reaches its maximum extent), to maintain a stable gaze on attended visual stimuli (Tatler and Wade, 2003). These involuntary eye movements are largely driven by vestibular activity arising in the semi-circular canals and are mediated by a “velocity storage mechanism” located across the vestibular nuclei, nucleus prepositus, and portions of the cerebellar network (Raphan et al., 1979; Raphan and Cohen, 1985; Yakushin et al., 2017). This mechanism lengthens the decay period of vestibular inputs while integrating them with input from the visual system (Raphan et al., 1979). When rotations are ended suddenly, angular head velocity signals persist in the velocity storage system for some time, causing the eyes to continue moving in the direction opposite the rotation.

There are a few similarities between postrotational nystagmus and the activity we observed in HD cells. (1) Postrotation bursting in HD cells matched the frequency of rotations and this bursting decreased with time, as is observed in postrotational nystagmus. (2) Postrotational nystagmus is more apparent when visual cues are absent during the postrotation period (Mowrer, 1935, 1937; Ter Braak, 1936; Krieger and Bender, 1956), and is thought to occur because subjects fixate on stable visual cues, which inhibits postrotational nystagmus eye movements and leads to “dumping” or resetting of the velocity storage system (Cohen et al., 1977; Raphan et al., 1979); similarly, we did not observe postrotational bursting in sessions when visual cues were available. (3) Postrotational nystagmus is disrupted by voluntary head movements (Dizio and Lackner, 1988) or if the head is off-tilt relative to the vertical axis (Guedry, 1965; Benson and Bodin, 1966; Schrader et al., 1985) because the conflicting vestibular input also leads to dumping of the velocity storage system (Raphan and Cohen, 1985); similarly, we also did not observe postrotational bursting in sessions where animals moved their head during and after the rotations.

Why does postrotation HD cell activity share so many characteristics with postrotational nystagmus? One possibility may be related to the velocity storage system described above. In the monkey this system is thought to be found in the rostral portions of the vestibular nuclei, such as the caudal ventral superior vestibular nucleus, medial vestibular nucleus, and lateral vestibular nucleus (Yokota et al., 1992; Yakushin et al., 2017). In the rat, the medial vestibular nucleus provides important vestibular input to the HD network via the supragenual nucleus (SGN; Biazoli et al., 2006). Stimulation of the nearby prepositus hypoglossi nuclei, an area thought to serve as a neural integrator (Cannon and Robinson, 1987), also evokes nystagmus (Yokota et al., 1992), and this region projects to the dorsal tegmental nucleus, a key node in the HD system (Bassett et al., 2007). One possibility is that the HD system receives input from the same velocity storage integrator as the vestibulo-ocular system. In the case of HD cells this integrator could help stabilize the network by (1) mediating activity coming from the visual system, compensating for rotational vestibular responses if stable visual cues are available and (2) storing and lengthening the high frequency signals from the semi-circular canals to provide a more accurate representation of angular head velocity during slow head movements (Cohen et al., 1977; Raphan et al., 1979).

Future experiments could seek to test this hypothesis by lesioning the nodulus and uvula of the vestibulo-cerebellum, which are involved in “dumping” the contents of the velocity storage mechanism (Waespe et al., 1985). Another approach would be to rotate rats in darkness before tilting their heads during the postrotation period as this procedure discharges the velocity storage mechanism in monkeys (Guedry, 1965) and humans (Schrader et al., 1985) and may therefore also decrease postrotation bursting in HD cells. Finally, in every species tested, postrotational nystagmus decreases after repeated exposures to rotational stimulation (Griffith, 1920; King, 1926; Hood and Pfaltz, 1954; Collins and Updegraff, 1966). We could not look for this effect in our current experiments because of the interspersed nature of our rotation sessions, but a future experiment could determine whether postrotation bursting in HD cells “habituates” with multiple exposures to the same rotation paradigm.

## Conclusions

In conclusion, by monitoring responses of HD cells during spatial disorientation, we found that they never become quiescent, they continue to fire coherently even when the animal is substantially disoriented, and individual cells no longer display directional or stable firing. This finding is consistent with an attractor network model of the HD system, where activity in the network is never abolished, but only shifts to different points or becomes distributed around the attractor ring. These shifts and distributed firing lead to a decrease in peak firing rates, and if strong enough, essentially result in the loss of directional tuning altogether. When visual cues were available, they only helped to maintain directionality during disorientation by a small amount or for a short period of time in the case of fast continuous rotations. In addition to visual and vestibular inputs, we also found that the HD network exhibits postrotational activity, which shares several features with postrotational nystagmus in the vestibulo-ocular system. One interpretation of this finding is that the HD network receives input from a velocity storage process that has its origins in the vestibular system. This process is commonly associated with the vestibulo-ocular reflex and maintaining an attended visual image focused on the fovea in the face of head movements. In the HD circuit, this process could also prove advantageous following disorientation by providing an additional mechanism for stabilization based on visual cues. More generally, this velocity storage mechanism would also mediate vestibular inputs to the HD system, lengthening these high-frequency outputs, which may provide a more accurate estimation of slow head movements. Additional experiments will be needed to test this potential input directly. Thus, the HD system utilizes multisensory integration from a variety of inputs to prevent disorientation. Although strong vestibular disruption, such as that occurring during continuous rotation, appears particularly difficult for the system to overcome, it appears that vestibular input is prioritized over other sensory types.

## Synthesis

Reviewing Editor: Michaël Zugaro, CNRS, Collège de France, Inserm Decisions are customarily a result of the Reviewing Editor and the peer reviewers coming together and discussing their recommendations until a consensus is reached. When revisions are invited, a fact-based synthesis statement explaining their decision and outlining what is needed to prepare a revision will be listed below. The following reviewer(s) agreed to reveal their identity: Gil Vantomme.

The reviews are summarized below. As you will see, they are supportive and only raise a few points that should be addressed.

The properties of head-direction cells and their major role in spatial navigation is well-known. However, it has been challenging to link head-direction cell activity and corresponding behavior. This work advances the field by providing a characterisation of head direction neuronal responses to putative vestibular stimulation and disorientation. The rationale and aims of this manuscript are well articulated and could be summarized as: what are the changes in HD cell activity during disorientation? How do visual, self-motion and vestibular inputs influence the HD cell activity during/after disorienting rotation? The authors used rotational disorientation in different condition (light/dark, freely moving/restrained, uni/bidirectional). This original approach is appropriate to address the main questions of this manuscript and helps disentangling the role of vestibular and visual inputs in modulating the head-direction cell activity. The authors carefully and extensively reported their methods and analyses throughout the manuscript. In summary, the authors cleverly record thalamic head-direction cells in rats during spatial disorientation and this approach brings new insight on the neural mechanisms underlying reorientation and is of great interest for computational and, in general, the head-direction field. The authors achieve a good balance between interpretation of their data and useful speculation on the role of visual, vestibular and self-locomotion inputs during disorientation. In particular, a novel result reported in this paper is the regular, post-rotational bursting of HD neurons, which correspond to the rotation frequency.

Main comments:

1. The order in which initial results are presented is confusing at first reading. The authors carefully described their experimental design in Fig 1 to Fig 3, displaying 3 example HD cells for each but no results. As is, these methodological figures could be moved to supplementary materials. The analysis of the impact of disorientation on HD cell properties are presented in Fig 4 (peak firing rate and burst index) and in Fig 7 (Rayleigh vector length and preferred direction), displaying results from all 3 experimental designs. The authors could consider whether reorganizing these 5 figures would help the flow of reading of the paper and would be worth the extensive rewriting work it represents.

- Suggested Fig1: Impact of alternating rotation in the presence and absence of visual cues on HD cell properties. Current Fig1 + Fig 4A, Fig 4D, 7A, 7D (removing continuous rotation and passive rotation data).

- Suggested Fig2: Impact of continuous rotation in the presence and absence of visual cues on HD cell properties. Current Fig2 + Fig 4B, 7B

- Suggested Fig3: Impact of continuous rotation in the presence and absence of visual cues and self-motion on HD cell properties. Current Fig3 + Fig 4C, 7C

2. It is puzzling that the directionality of the cells did not decrease in darkness - the examples in the supplementary data show that the majority of cells did show a lower firing rate in the dark in Expt. 1, but maybe less so in Expt. 2.

Minor comments:

- The comparison with previous findings of inversion is not set up in the Introduction or the Results; a little more lead-in on this would be helpful to the reader.

- The statement on the velocity storage process (lines 653-656) seems overstated - this is not a direct finding, but a possible interpretation of the results, and should be qualified as such.

Corrections:

- Lines 53 and 54: head direction is abbreviated twice

- Anterodorsal thalamus is abbreviated for the first time on line 99 instead of 91

- Preferred firing direction is abbreviated for the first time line 372 instead of 221.

- Dorsal tegmental nucleus is used twice in the text and is abbreviated twice, lines 477 and 626.

- In Figure 2, the X axis on Cell 3 differs in the Visual rotation session.

- Legend of Figure 6, lines 305-306 “...the text provides the result of multiple comparisons...”

- Panel D of Figure 6 is not mentioned in the corresponding paragraph

- Line 821: correct “institution"

## Author Response

The properties of head-direction cells and their major role in spatial navigation is well-known. However, it has been challenging to link head-direction cell activity and corresponding behavior. This work advances the field by providing a characterization of head direction neuronal responses to putative vestibular stimulation and disorientation. The rationale and aims of this manuscript are well articulated and could be summarized as: what are the changes in HD cell activity during disorientation? How do visual, self-motion and vestibular inputs influence the HD cell activity during/after disorienting rotation? The authors used rotational disorientation in different condition (light/dark, freely moving/restrained, uni/bidirectional). This original approach is appropriate to address the main questions of this manuscript and helps disentangling the role of vestibular and visual inputs in modulating the head-direction cell activity. The authors carefully and extensively reported their methods and analyses throughout the manuscript. In summary, the authors cleverly record thalamic head-direction cells in rats during spatial disorientation and this approach brings new insight on the neural mechanisms underlying reorientation and is of great interest for computational and, in general, the head-direction field. The authors achieve a good balance between interpretation of their data and useful speculation on the role of visual, vestibular and self-locomotion inputs during disorientation. In particular, a novel result reported in this paper is the regular, post-rotational bursting of HD neurons, which correspond to the rotation frequency.

We thank the Reviewers for their kind remarks and detailed comments on the manuscript. We hope to have addressed each of the Reviewers’ concerns in our replies below. In addition, we have:

a) Fixed an error in one of the figures where the inversion data and their baseline group were reversed (Fig. 7 in current manuscript). This updated change does not affect the statistics, main text, or conclusions of the paper as these were correctly reported before in the text of the original manuscript.

b) Added a control analysis to the inversion results, which takes into account the differences in cell PFD firing rates (Fig. 7G-I in current manuscript). This analysis shows that differences in ISI duration, but not burst index, can be partially explained by PFD firing rates.

c) We have switched our multiple linear regression analysis, which was previously conducted in SPSS, to a generalized linear model, which is now conducted within Matlab. This switch was made to improve the reproducibility of our Matlab analysis code, which we aim to upload to an online repository, and reduce its dependence on 3rd party software, which readers may not have access to. Because both approaches are based on the same underlying principles, this analysis has had a minimal effect on our statistics and figures, and has not changed the main text or conclusions of the paper. A description of this approach has been added to the methods.

Main comments:

1. The order in which initial results are presented is confusing at first reading. The authors carefully described their experimental design in Fig 1 to Fig 3, displaying 3 example HD cells for each but no results. As is, these methodological figures could be moved to supplementary materials. The analysis of the impact of disorientation on HD cell properties are presented in Fig 4 (peak firing rate and burst index) and in Fig 7 (Rayleigh vector length and preferred direction), displaying results from all 3 experimental designs. The authors could consider whether reorganizing these 5 figures would help the flow of reading of the paper and would be worth the extensive rewriting work it represents.

- Suggested Fig1: Impact of alternating rotation in the presence and absence of visual cues on HD cell properties. Current Fig1 + Fig 4A, Fig 4D, 7A, 7D (removing continuous rotation and passive rotation data).

- Suggested Fig2: Impact of continuous rotation in the presence and absence of visual cues on HD cell properties. Current Fig 2 + Fig 4B, 7B.

- Suggested Fig3: Impact of continuous rotation in the presence and absence of visual cues and self-motion on HD cell properties. Current Fig 3 + Fig 4C, 7C.

We feel that having the protocols clearly schematized is important for the Readers’ understanding of the experiments that were performed. However, in line with the Reviewers’ comments about the flow of reading, we have condensed Figures 1-3 into one figure with simplified example cells (Fig. 1 in current manuscript). We have also followed the Reviewers’ advice and moved Figures 4 and 7 (the main data figures where all 3 experiments are consolidated) to the first figures after Figure 1. These 3 figures (Fig. 2-4 in current manuscript) roughly correspond to the layout suggested by the Reviewers, but with the addition of example cells. The main text has been rearranged to reflect this change and we agree that it improves the flow of reading. In addition, we moved Figure 5 (AHV control analysis) and Figure 8 (windowed tuning curves throughout rotation) to supplementary information. The figure comparing the effects of rotation and inversion has also been moved to the end of the results section (Fig. 7 in current manuscript) in order to report the main results in a consecutive manner.

2. It is puzzling that the directionality of the cells did not decrease in darkness - the examples in the supplementary data show that the majority of cells did show a lower firing rate in the dark in Expt. 1, but maybe less so in Expt. 2.

We have added the following text to the discussion which we hope answers the Reviewers’ question:

A GLM fitted to the data from all 3 experiments highlighted a significant negative effect of darkness on directionality, separate to any effect of rotation speed. However, this effect was mild and ranked as the weakest among the significant coefficients. Furthermore, when we directly compared baseline sessions in the light to baseline sessions in the dark, we did not find a significant difference in directionality. This pattern of results suggests a very mild effect of darkness alone and is likely because our dark baseline sessions were quite short (4 mins), suggesting that there was little time for the HD network to drift or lose directionality. This result is also consistent with previous reports (30, 50), which suggest that HD cells drift by about 1.5{degree sign}/min in darkness (52).

These results are in agreement with the cells shown in supplementary data. Although peak firing rates often decreased, directionality (black and yellow text, Extended Data Figures 1-3 and 1-4) was often unchanged in darkness.

Minor comments:

- The comparison with previous findings of inversion is not set up in the Introduction or the Results; a little more lead-in on this would be helpful to the reader.

We have added text to the end of the introduction for this purpose:

Additionally, because both inversion and rotation lead to disorientation in humans (38,39) and rats (35,40-42), we sought to determine if these conditions are also associated with comparable disruption to the HD system, which may point to a common underlying mechanism.

In addition, we have completely rewritten the results section regarding inversion in order to provide more context to these results.

- The statement on the velocity storage process (lines 653-656) seems overstated - this is not a direct finding, but a possible interpretation of the results, and should be qualified as such.

We have modified some of the text around these lines in order to clarify that this statement is one interpretation of the results which needs to be tested directly in the future. The text now reads:

In addition to visual and vestibular inputs, we also found that the HD network exhibits post-rotational activity, which shares several features with post-rotational nystagmus in the vestibulo-ocular system. One interpretation of this finding is that the HD network receives input from a velocity storage process that has its origins in the vestibular system. [...] Further experiments will be needed to test this potential input directly.

We have also modified the abstract to reflect this point as well:

This post-rotational bursting shared several striking similarities with post-rotational “nystagmus” in the vestibulo-ocular system, consistent with the interpretation that the HD system receives input from a vestibular velocity storage mechanism that works to reduce spatial disorientation following rotation.

Corrections:

- Lines 53 and 54: head direction is abbreviated twice

This twice abbreviation has been corrected.

- Anterodorsal thalamus is abbreviated for the first time on line 99 instead of 91

This abbreviation has been corrected.

- Preferred firing direction is abbreviated for the first time line 372 instead of 221.

This abbreviation has been corrected.

- Dorsal tegmental nucleus is used twice in the text and is abbreviated twice, lines 477 and 626.

Given the infrequency of use of this area in the text, we have removed both abbreviations.

- In Figure 2, the X axis on Cell 3 differs in the Visual rotation session.

This figure has now been replaced and this concern is no longer an issue.

- Legend of Figure 6, lines 305-306 “...the text provides the result of multiple comparisons...”

This figure is Figure 7 in the current manuscript. The text has been updated to:

Tests are one-way ANOVAs, the statistical significance of post-hoc tests is given by horizontal brackets and asterisks above the plots (* = p < .05, ** = p < .01, *** = p < .001).

- Panel D of Figure 6 is not mentioned in the corresponding paragraph.

This panel is now mentioned in the text.

- Line 821: correct “institution"

The spelling has been corrected.

## References

[B1] Bassett JP, Tullman ML, Taube JS (2007) Lesions of the tegmentomammillary circuit in the head direction system disrupt the head direction signal in the anterior thalamus. J Neurosci 27:7564–7577. 10.1523/JNEUROSCI.0268-07.2007 17626218 PMC6672597

[B2] Benson AJ, Bodin MA (1966) Interaction of linear and angular accelerations on vestibular receptors in man. Aerosp Med 37:144–154. 5295433

[B3] Berens P (2009) CircStat: a MATLAB toolbox for circular statistics. J Stat Soft 31. 10.18637/jss.v031.i10

[B4] Biazoli CE, Goto M, Campos AMP, Canteras NS (2006) The supragenual nucleus: a putative relay station for ascending vestibular signs to head direction cells. Brain Res 1094:138–148. 10.1016/j.brainres.2006.03.101 16684515

[B5] Calton JL, Taube JS (2005) Degradation of head direction cell activity during inverted locomotion. J Neurosci 25:2420–2428. 10.1523/JNEUROSCI.3511-04.2005 15745969 PMC6726092

[B6] Cannon SC, Robinson DA (1987) Loss of the neural integrator of the oculomotor system from brain stem lesions in monkey. J Neurophysiol 57:1383–1409. 10.1152/jn.1987.57.5.1383 3585473

[B7] Chen LL, Lin LH, Barnes CA, McNaughton BL (1994) Head-direction cells in the rat posterior cortex - II. Contributions of visual and ideothetic information to the directional firing. Exp Brain Res 101:24–34. 10.1007/BF00243213 7843299

[B8] Cheung B (2013) Spatial disorientation: more than just illusion. Aviat Space Environ Med 84:1211–1214. 10.3357/asem.3657.2013 24279238

[B9] Clark BJ, Taube JS (2012) Vestibular and attractor network basis of the head direction cell signal in subcortical circuits. Front Neural Circuits 6:7. 10.3389/fncir.2012.00007 22454618 PMC3308332

[B10] Cohen B, Matsuo V, Raphan T (1977) Quantitative analysis of the velocity characteristics of optokinetic nystagmus and optokinetic after-nystagmus. J Physiol 270:321–344. 10.1113/jphysiol.1977.sp011955 409838 PMC1353516

[B11] Collins WE, Updegraff BP (1966) A comparison of nystagmus habituation in the cat and the dog. Acta Otolaryngol 62:19–26. 10.3109/00016486609119546 5967873

[B12] Cullen KE (2014) The neural encoding of self-generated and externally applied movement: implications for the perception of self-motion and spatial memory. Front Integr Neurosci 7:108. 10.3389/fnint.2013.00108 24454282 PMC3888934

[B13] Cullen KE, Taube JS (2017) Our sense of direction: progress, controversies and challenges. Nat Neurosci 20:1465–1473. 10.1038/nn.4658 29073639 PMC10278035

[B14] Day BL, Fitzpatrick RC (2005) Virtual head rotation reveals a process of route reconstruction from human vestibular signals. J Physiol 567:591–597. 10.1113/jphysiol.2005.092544 16002439 PMC1474201

[B15] Dizio P, Lackner JR (1988) The effects of gravitoinertial force level and head movements on post-rotational nystagmus and illusory after-rotation. Exp Brain Res 70:485–495. 10.1007/BF00247597 3384050

[B16] Dudchenko PA, Goodridge JP, Seiterle DA, Taube JS (1997) Effects of repeated disorientation on the acquisition of spatial tasks in rats: dissociation between the appetitive radial arm maze and aversive water maze. J Exp Psychol Anim Behav Process 23:194–210. 10.1037/0097-7403.23.2.194 9095542

[B17] Fischer AJEM, Huygen PLM, Kuijpers W (1979) Electronystagmography in the laboratory rat. Acta Otolaryngol 88:412–419. 10.3109/00016487909137186 316963

[B18] Garcia D (2010) Robust smoothing of gridded data in one and higher dimensions with missing values. Comput Stat Data Anal 54:1167–1178. 10.1016/j.csda.2009.09.020 24795488 PMC4008475

[B19] Garcia D (2011) A fast all-in-one method for automated post-processing of PIV data. Exp Fluids 50:1247–1259. 10.1007/s00348-010-0985-y 24795497 PMC4006822

[B20] Gibb R, Ercoline B, Scharff L (2011) Spatial disorientation: decades of pilot fatalities. Aviat Space Environ Med 82:717–724. 10.3357/asem.3048.2011 21748911

[B21] Gibson B, Butler WN, Taube JS (2013) The head-direction signal is critical for navigation requiring a cognitive map but not for learning a spatial habit. Curr Biol 23:1536–1540. 10.1016/j.cub.2013.06.030 23891111 PMC4106916

[B22] Gillingham KK (1992) The spatial disorientation problem in the United States Air Force. J Vestib Res 2:297–306. 1342404

[B23] Gillingham KK, Previc FH (1996) Spatial orientation in flight. Baltimore: Williams and Wilkins.

[B24] Golob EJ, Wolk DA, Taube JS (1998) Recordings of postsubiculum head direction cells following lesions of the laterodorsal thalamic nucleus. Brain Res 780:9–19. 10.1016/s0006-8993(97)01076-7 9473564

[B25] Goodridge JP, Touretzky DS (2000) Modeling attractor deformation in the rodent head-direction system. J Neurophysiol 83:3402–3410. 10.1152/jn.2000.83.6.3402 10848558

[B26] Goodridge JP, Dudchenko PA, Worboys KA, Golob EJ, Taube JS (1998) Cue control and head direction cells. Behav Neurosci 112:749–761. 10.1037/0735-7044.112.4.749 9733184

[B27] Grieves RM, Jeffery KJ (2017) The representation of space in the brain. Behav Processes 135:113–131. 10.1016/j.beproc.2016.12.012 28034697

[B28] Grieves RM, Shinder ME, Rosow L, Kenna M, Taube JS (2022) The neural correlates of spatial disorientation in head direction cells: summary dataset and analysis package. Mendeley Data, V1, 10.17632/6v63gfk2y7.1. 10.1523/ENEURO.0174-22.2022 PMC9770022 36635237

[B29] Griffith CR (1920) The decrease of after-nystagmus during repeated rotation. Laryngoscope 30:129–137.

[B30] Guedry FE (1965) Orientation of the rotation-axis relative to gravity: its influence on nystagmus and the sensation of rotation. Acta Otolaryngol 60:30–48. 10.3109/00016486509126986 14337956

[B31] Guedry FE, Stockwell CW, Gilson RD (1971) Comparison of subjective responses to semicircular canal stimulation produced by rotation about different axes. Acta Otolaryngol 72:101–106. 10.3109/00016487109122461 5095486

[B32] Harland B, Grieves RM, Bett D, Stentiford R, Wood ER, Dudchenko PA (2017) Lesions of the head direction cell system increase hippocampal place field repetition. Curr Biol 27:2706–2712.e2. 10.1016/j.cub.2017.07.071 28867207 PMC5607353

[B33] Hood JD, Pfaltz CR (1954) Observations upon the effects of repeated stimulation upon rotational and caloric nystagmus. J Physiol 124:130–144. 10.1113/jphysiol.1954.sp005092 13163874 PMC1366247

[B34] Israël I, Bronstein AM, Kanayama R, Faldon M, Gresty MA (1996) Visual and vestibular factors influencing vestibular “navigation.” Exp Brain Res 112:411–419. 10.1007/BF00227947 9007543

[B35] Kempter R, Leibold C, Buzsáki G, Diba K, Schmidt R (2012) Quantifying circular–linear associations: hippocampal phase precession. J Neurosci Methods 207:113–124. 10.1016/j.jneumeth.2012.03.007 22487609

[B36] Keshavarzi S, Bracey EF, Faville RA, Campagner D, Tyson AL, Lenzi SC, Branco T, Margrie TW (2022) Multisensory coding of angular head velocity in the retrosplenial cortex. Neuron 110:532–543. 34788632 10.1016/j.neuron.2021.10.031 PMC8823706

[B37] King BG (1926) The influence of repeated rotations on decerebrate and on blinded squabs. J Comp Psychol 6:399–421. 10.1037/h0071155

[B38] Knierim JJ, Kudrimoti HS, McNaughton BL (1995) Place cells, head direction cells, and the learning of landmark stability. J Neurosci 15:1648–1659. 10.1523/JNEUROSCI.15-03-01648.1995 7891125 PMC6578145

[B39] Knierim JJ, Kudrimoti HS, McNaughton BL (1998) Interactions between idiothetic cues and external landmarks in the control of place cells and head direction cells. J Neurophysiol 80:425–446. 10.1152/jn.1998.80.1.425 9658061

[B40] Krieger HP, Bender MB (1956) Optokinetic afternystagmus in the monkey. Electroencephalogr Clin Neurophysiol 8:97–106. 10.1016/0013-4694(56)90036-0 13294067

[B41] Kubie JL (1984) A driveable bundle of microwires for collecting single-unit data from freely-moving rats. Physiol Behav 32:115–118. 10.1016/0031-9384(84)90080-5 6718521

[B42] Loomis JM, Klatzky RL, Golledge RG, Cicinelli JG, Pellegrino JW, Fry PA (1993) Nonvisual navigation by blind and sighted: assessment of path integration ability. J Exp Psychol Gen 122:73–91. 10.1037/0096-3445.122.1.73 8440978

[B43] Mach E (1875) Grundlinien der Lehre von den Bewegungsempfindungen. Leipzig: Engelmann.

[B44] Marlinsky VV (1999) Vestibular and vestibulo-proprioceptive perception of motion in the horizontal plane in blindfolded man - II. Estimations of rotations about the earth-vertical axis. Neuroscience 90:395–401. 10.1016/s0306-4522(98)00449-7 10215145

[B45] Martin GM, Harley CW, Smith AR, Hoyles ES, Hynes CA (1997) Spatial disorientation blocks reliable goal location on a plus maze but does not prevent goal location in the Morris maze. J Exp Psychol Anim Behav Process 23:183–193. 10.1037/0097-7403.23.2.183 9095541

[B46] Mizumori SJY, Williams JD (1993) Directionally selective mnemonic properties of neurons in the lateral dorsal nucleus of the thalamus of rats. J Neurosci 13:4015–4028. 10.1523/JNEUROSCI.13-09-04015.1993 8366357 PMC6576470

[B47] Moser EI, Kropff E, Moser M-B (2008) Place cells, grid cells, and the brain’s spatial representation system. Annu Rev Neurosci 31:69–89. 10.1146/annurev.neuro.31.061307.090723 18284371

[B48] Mowrer OH (1935) Some neglected factors which influence the duration of post-rotational nystagmus. Acta Otolaryngol 22:1–23. 10.3109/00016483509118081

[B49] Mowrer OH (1937) The influence of vision during bodily rotation upon the duration of post-rotational vestibular nystagmus. Acta Otolaryngol 25:351–364. 10.3109/00016483709127972

[B50] Muir GM, Brown JE, Carey JP, Hirvonen TP, Della Santina CC, Minor LB, Taube JS (2009) Disruption of the head direction cell signal after occlusion of the semicircular canals in the freely moving chinchilla. J Neurosci 29:14521–14533. 10.1523/JNEUROSCI.3450-09.2009 19923286 PMC2821030

[B51] Paxinos G, Watson C (2006) The rat brain in stereotaxic coordinates. San Diego: Elsevier. 10.1016/0165-0270(80)90021-7 6110810

[B52] Peck JR, Taube JS (2017) The postrhinal cortex is not necessary for landmark control in rat head direction cells. Hippocampus 27:156–168. 10.1002/hipo.22680 27860052 PMC5235971

[B53] Peters RA (1969) Dynamics of the vestibular system and their relation to motion perception, spatial disorientation, and illusions (No. TR-168-1). NASA Contract Rep NASA CR January:1–223. 5306945

[B54] Plotnik M, Freeman S, Sohmer H, Elidan J (1999) The effect of head orientation on the vestibular evoked potentials to linear acceleration impulses in rats. Am J Otol 20:735–740. 10565717

[B55] Previc FH, Ercoline WR (2004) Spatial disorientation in aviation, Progress in astronautics and aeronautics. Reston: American Institute of Aeronautics and Astronautics.

[B56] Raphan T, Cohen B (1985) Velocity storage and the ocular response to multidimensional vestibular stimuli. Rev Oculomot Res 1:123–143. 3940025

[B57] Raphan T, Matsuo V, Cohen B (1979) Velocity storage in the vestibulo-ocular reflex arc (VOR). Exp Brain Res 35:229–248. 10.1007/BF00236613 108122

[B58] Sargent J, Dopkins S, Philbeck J, Modarres R (2008) Spatial memory during progressive disorientation. J Exp Psychol Learn Mem Cogn 34:602–615. 10.1037/0278-7393.34.3.602 18444759 PMC2883724

[B59] Schrader V, Koenig E, Dichgans J (1985) The effect of lateral head tilt on horizontal postrotatory nystagmus I and II and the Purkinje effect. Acta Otolaryngol 100:98–105. 10.3109/00016488509108593 4024896

[B60] Semenov LV, Bures J (1989) Vestibular stimulation disrupts acquisition of place navigation in the Morris water tank task. Behav Neural Biol 51:346–363. 10.1016/s0163-1047(89)90987-4 2730498

[B61] Sharp PE, Tinkelman A, Cho J (2001) Angular velocity and head direction signals recorded from the dorsal tegmental nucleus of gudden in the rat: implications for path integration in the head direction cell circuit. Behav Neurosci 115:571–588. 11439447

[B62] Shinder ME, Taube JS (2011) Active and passive movement are encoded equally by head direction cells in the anterodorsal thalamus. J Neurophysiol 106:788–800. 10.1152/jn.01098.2010 21613594 PMC3154800

[B63] Shinder ME, Taube JS (2019) Three-dimensional tuning of head direction cells in rats. J Neurophysiol 121:4–37. 10.1152/jn.00880.2017 30379631 PMC6383655

[B64] Skaggs WE, Knierim JJ, Kudrimoti HS, McNaughton BL (1995) A model of the neural basis of the rat’s sense of direction. Adv Neural Inf Process Syst 7:173–180. 11539168

[B65] Stackman RW, Taube JS (1997) Firing properties of head direction cells in the rat anterior thalamic nucleus: dependence on vestibular input. J Neurosci 17:4349–4358. 10.1523/JNEUROSCI.17-11-04349.1997 9151751 PMC1489676

[B66] Stackman RW, Golob EJ, Bassett JP, Taube JS (2003) Passive transport disrupts directional path integration by rat head direction cells. J Neurophysiol 90:2862–2874. 10.1152/jn.00346.2003 12890795

[B67] Steinhausen W (1933) Über die Beobachtung der Cupula in den Bogengangsampullen des Labyrinths des lebenden Hechts. Pflugers Arch 232:500–512. 10.1007/BF01754806

[B68] Tatler BW, Wade NJ (2003) On nystagmus, saccades, and fixations. Perception 32:167–184. 10.1068/p3395 12696663

[B69] Taube JS (1995) Head direction cells recorded in the anterior thalamic nuclei of freely moving rats. J Neurosci 15:70–86. 10.1523/JNEUROSCI.15-01-00070.1995 7823153 PMC6578288

[B70] Taube JS (2007) The head direction signal: origins and sensory-motor integration. Annu Rev Neurosci 30:181–207. 10.1146/annurev.neuro.29.051605.112854 17341158

[B71] Taube JS, Muller RU (1998) Comparisons of head direction cell activity in the postsubiculum and anterior thalamus of freely moving rats. Hippocampus 8:87–108. 10.1002/(SICI)1098-1063(1998)8:2<87::AID-HIPO1>3.0.CO;2-4 9572715

[B72] Taube JS, Muller RU, Ranck JB (1990a) Head-direction cells recorded from the postsubiculum in freely moving rats. II. Effects of environmental manipulations. J Neurosci 10:436–447. 10.1523/JNEUROSCI.10-02-00436.1990 2303852 PMC6570161

[B73] Taube JS, Muller RU, Ranck JB (1990b) Head-direction cells recorded from the postsubiculum in freely moving rats. I. Description and quantitative analysis. J Neurosci 10:420–435. 10.1523/JNEUROSCI.10-02-00420.1990 2303851 PMC6570151

[B74] Taube JS, Stackman RW, Calton JL, Oman CM (2004) Rat head direction cell responses in zero-gravity parabolic flight. J Neurophysiol 92:2887–2997. 10.1152/jn.00887.2003 15212426

[B75] Ter Braak J (1936) Untersuchungen über optokinetischen Nystagmus. Arch Neerl Physiol 21:309–376.

[B76] Valerio S, Clark BJ, Chan JHM, Frost CP, Harris MJ, Taube JS (2010) Directional learning, but no spatial mapping by rats performing a navigational task in an inverted orientation. Neurobiol Learn Mem 93:495–505. 10.1016/j.nlm.2010.01.007 20109566 PMC2862784

[B77] Waespe W, Cohen B, Raphan T (1985) Dynamic modification of the vestibulo-ocular reflex by the nodulus and uvula. Science 228:199–202. 10.1126/science.3871968 3871968

[B78] Wallace DG, Hines DJ, Pellis SM, Whishaw IQ (2002) Vestibular information is required for dead reckoning in the rat. J Neurosci 22:10009–10017. 10.1523/JNEUROSCI.22-22-10009.2002 12427858 PMC6757817

[B79] Walsh EG (1960) Perception of linear motion following unilateral labyrinthectomy: variation of threshold according to the orientation of the head. J Physiol 153:350–357. 10.1113/jphysiol.1960.sp006538 13782901 PMC1359752

[B80] Wang RF, Spelke ES (2000) Updating egocentric representations in human navigation. Cognition 77:215–250. 10.1016/s0010-0277(00)00105-0 11018510

[B81] Whishaw IQ, Maaswinkel H (1998) Rats with fimbria–fornix lesions are impaired in path integration: a role for the hippocampus in “sense of direction.” J Neurosci 18:3050–3058. 10.1523/JNEUROSCI.18-08-03050.1998 PMC6792581 9526022

[B82] Wiener SI (1993) Spatial and behavioral correlates of striatal neurons in rats performing a self-initiated navigation task. J Neurosci 13:3802–3817. 10.1523/JNEUROSCI.13-09-03802.1993 8366346 PMC6576451

[B83] Winter SS, Clark BJ, Taube JS (2015) Disruption of the head direction cell network impairs the parahippocampal grid cell signal. Science 347:870–874. 10.1126/science.1259591 25700518 PMC4476794

[B84] Yakushin SB, Raphan T, Cohen B (2017) Coding of velocity storage in the vestibular nuclei. Front Neurol 8:386. 10.3389/fneur.2017.00386 28861030 PMC5561016

[B85] Yoder RM, Taube JS (2009) Head direction cell activity in mice: robust directional signal depends on intact otolith organs. J Neurosci 29:1061–1076. 10.1523/JNEUROSCI.1679-08.2009 19176815 PMC2768409

[B86] Yoder RM, Taube JS (2014) The vestibular contribution to the head direction signal and navigation. Front Integr Neurosci 8:32. 10.3389/fnint.2014.00032 24795578 PMC4001061

[B87] Yokota JI, Reisine H, Cohen B (1992) Nystagmus induced by electrical stimulation of the vestibular and prepositus hypoglossi nuclei in the monkey: evidence for site of induction of velocity storage. Exp Brain Res 92:123–138. 1486947 10.1007/BF00230389

[B88] Zhang K (1996) Representation of spatial orientation by the intrinsic dynamics of the head-direction cell ensemble: a theory. J Neurosci 16:2112–2126. 10.1523/JNEUROSCI.16-06-02112.1996 8604055 PMC6578512

[B89] Zugaro MB (2018) Freely moving animal (FMA) toolbox. GitHub repository. https://github.com/michael-zugaro/FMAToolbox.

