# Supplementary figures and images for: The Neural Correlates of Spatial Disorientation in Head Direction Cells
Source: eNeuro. 2022 Dec 16;9(6):ENEURO.0174-22.2022. doi: 10.1523/ENEURO.0174-22.2022 (PMC9770022; doi:10.1523/ENEURO.0174-22.2022)

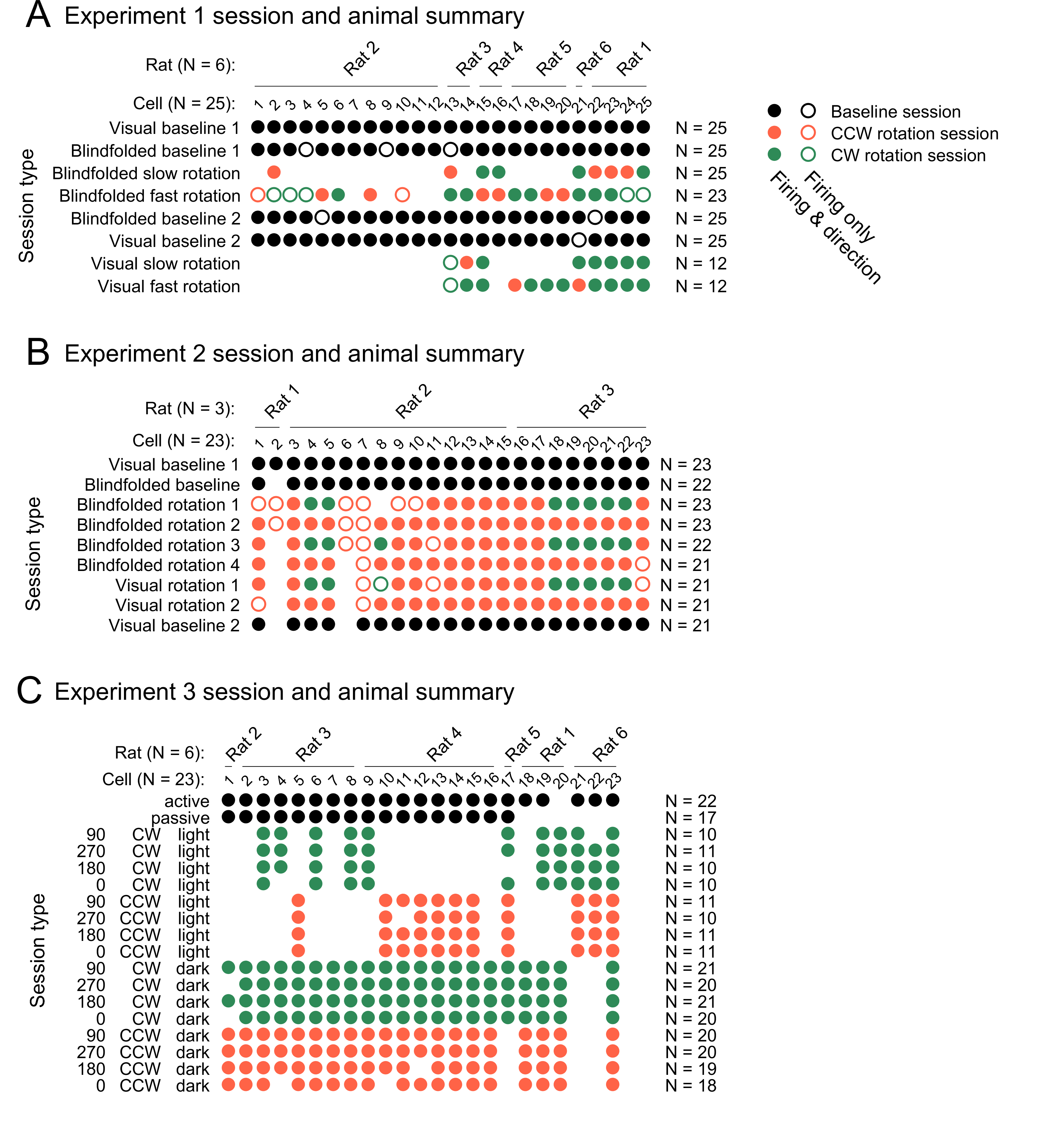

Supplement: Extended Data Figure 1-1 — Session and animal summary tables. A–C, Session and cell breakdown for Experiments 1–3. “Firing & direction” denotes sessions where cell spiking statistics (i.e., burst index and firing rate) and directional statistics (i.e., Rayleigh vector length and PFD drift) were analyzed, “firing only” denotes sessions where only cell spiking statistics were analyzed. In panel C, condition descriptors for session type denote (from left to right): the starting angle relative to the cell’s PFD (0, 90, 180, or 270), rotation direction (CW or CCW), and illumination condition (light or dark). Download Figure 1-1, TIF file. [file enu-eN-NWR-0174-22-s02.tif]

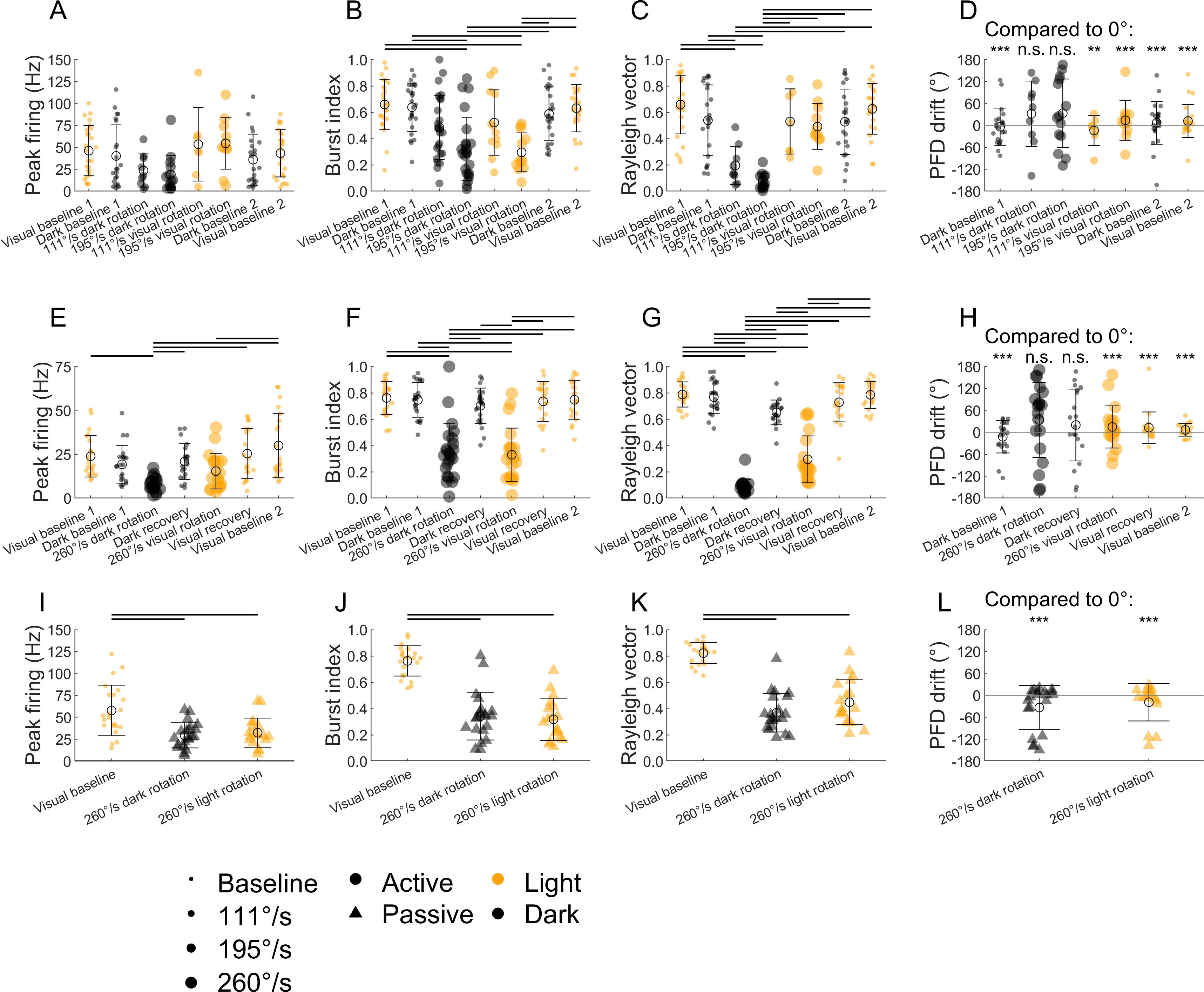

Supplement: Extended Data Figure 2-1 — Raw parameter values for all three experiments and statistical comparisons within experiments. Horizontal lines denote a significant post hoc comparison (p < 0.05; Dunn–Sidak corrected). For panels D, H, and L, drift values are relative to the first visual baseline session (i.e., 90° denotes a 90° CCW drift away from baseline) and the text gives the result of Holm–Bonferroni corrected v-tests for nonuniformity around a mean direction of 0°, a significant value here denotes clustering around 0° and thus a stable PFD (n.s. = p > 0.05, *p < 0.05, **p < 0.01, ***p < 0.001). A, Peak firing rates in Experiment 1 (F(7,127) = 2.3, p = 0.0317, η2 = 0.11). B, Burst index in Experiment 1 (F(5,116) = 10.3, p < 0.0001, η2 = 0.31). C, Directionality in Experiment 1 (F(5,82) = 17.6, p < 0.0001, η2 = 0.52). D, PFD stability in Experiment 1. E, Peak firing rates in Experiment 2 (F(6,138) = 7.2, p < 0.0001, η2 = 0.24). F, Burst index in Experiment 2 (F(4,104) = 5.3, p = 0.0006, η2 = 0.17). G, Directionality in Experiment 2 (F(6,138) = 112.2, p < 0.0001, η2 = 0.83). H, PFD stability in Experiment 2. I, Peak firing rates in Experiment 3 (F(2,58) = 11.4, p = 0.0001, η2 = 0.28). J, Burst index in Experiment 3 (F(2,58) = 55.3, p < 0.0001, η2 = 0.66). K, Directionality in Experiment 3 (F(2,58) = 68.8, p < 0.0001, η2 = 0.70). L, PFD stability in Experiment 3. Download Figure 2-1, TIF file. [file enu-eN-NWR-0174-22-s07.tif]

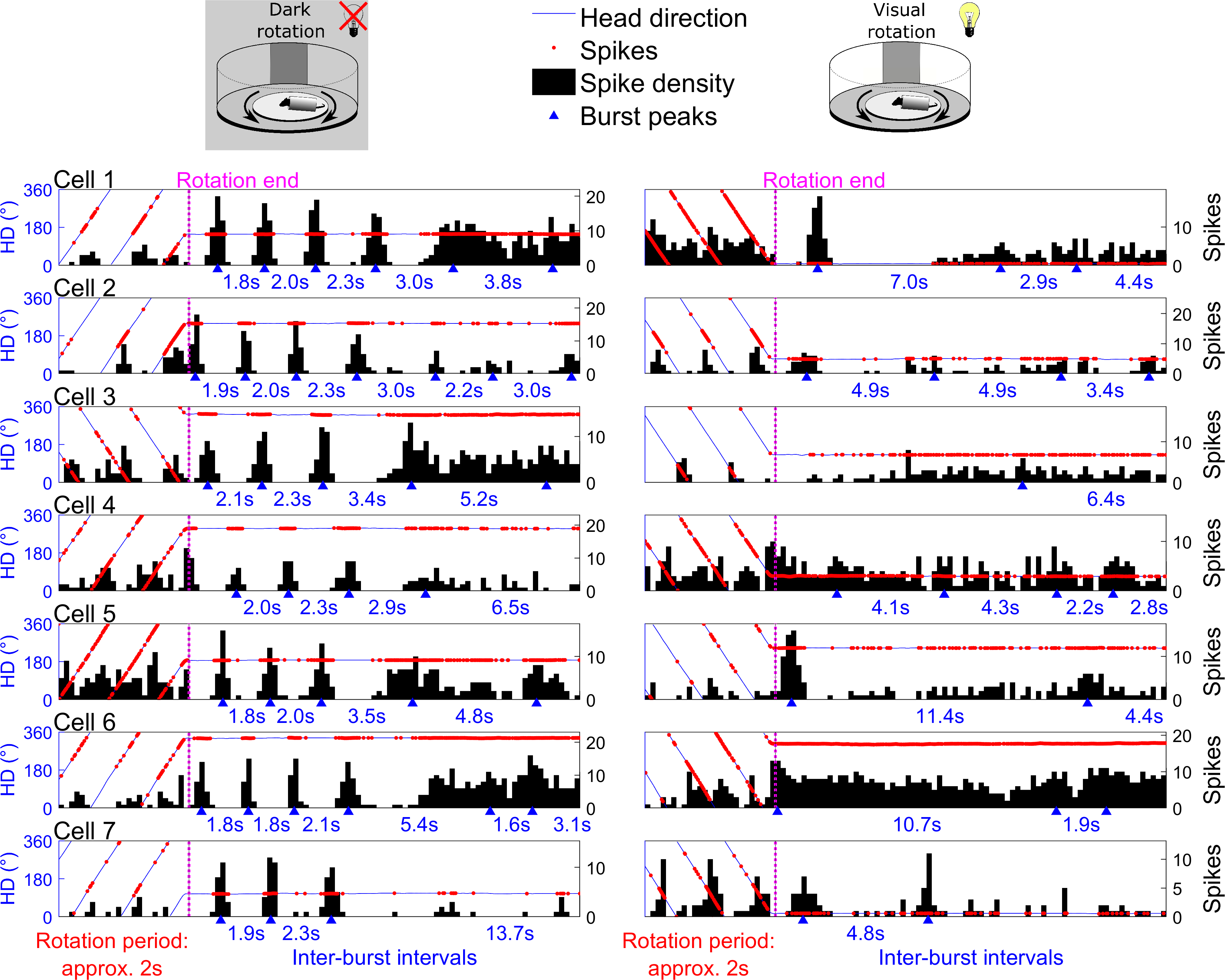

Supplement: Extended Data Figure 6-1 — Additional example cells showing postrotational bursting in Experiment 3, where rats were head-fixed and restrained during rotations. Example cells, one per row, left column shows activity in a dark rotation session, right column shows activity for the same cell in a rotation session in the light. Sessions are clipped to the end of the rotation phase (from 5 s before to 15 s after rotations ended). Blue lines denote the animal’s HD, red markers represent action potentials, black areas show a spike histogram (200-ms bins). Blue triangles denote detected spike bursts (Materials and Methods, Spike bursts), blue text between two triangles gives the duration between these bursts. In the dark, cells fired bursts of spikes after the rotations ended. Initial bursts occurred at a frequency close to the rotation frequency, but the time between consecutive bursts increased steadily. In the light, postrotational bursting was absent. Download Figure 6-1, TIF file. [file enu-eN-NWR-0174-22-s13.tif]

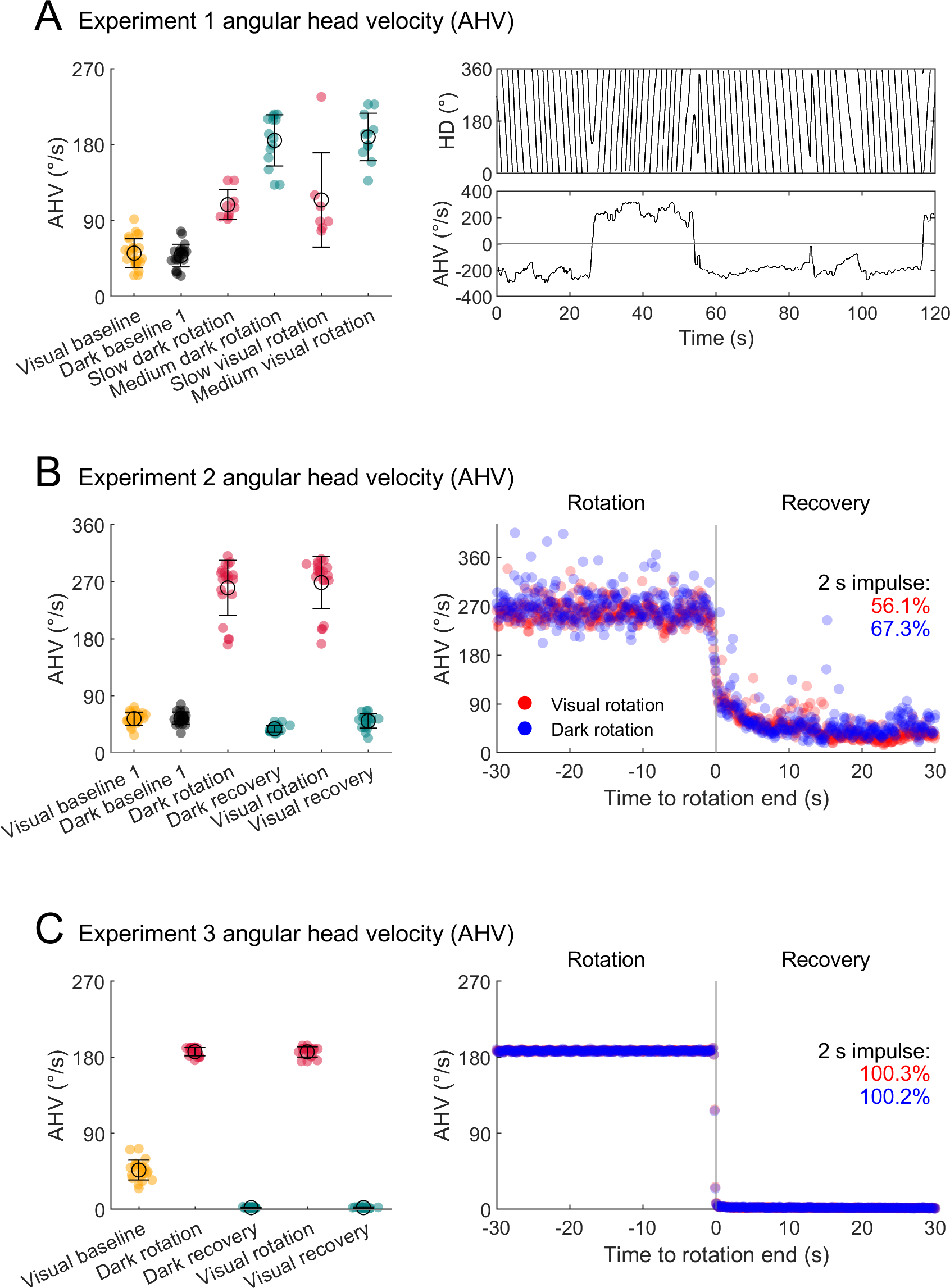

Supplement: Extended Data Figure 1-2 — In all plots, the data for dark rotation sessions and visual rotation sessions were averaged to give a single value for each session type. A, Left, The AHV of the animal in different sessions and experiment phases. Filled markers represent sessions, lines and circular markers denote mean and SD. AHV was consistent in the dark/light slow/medium rotation sessions. Right, Head direction and AHV throughout an example rotation session. B, Left, Same as A. AHV was consistent in the dark/light rotation sessions. Right, Mean AHV for each session around the end of rotations. Text gives the 2-s impulse: the change in AHV between t = −1 and t = +1 divided by the change in AHV between t = −20 and t = +20. The higher this value, the faster AHV decreased after rotations ended. C, Same as B except for Experiment 3. AHV was very consistent in the dark/light rotation sessions. The 2-s impulse indicates that AHV decreased immediately at the end of rotations. Download Figure 1-2, TIF file. [file enu-eN-NWR-0174-22-s03.tif]

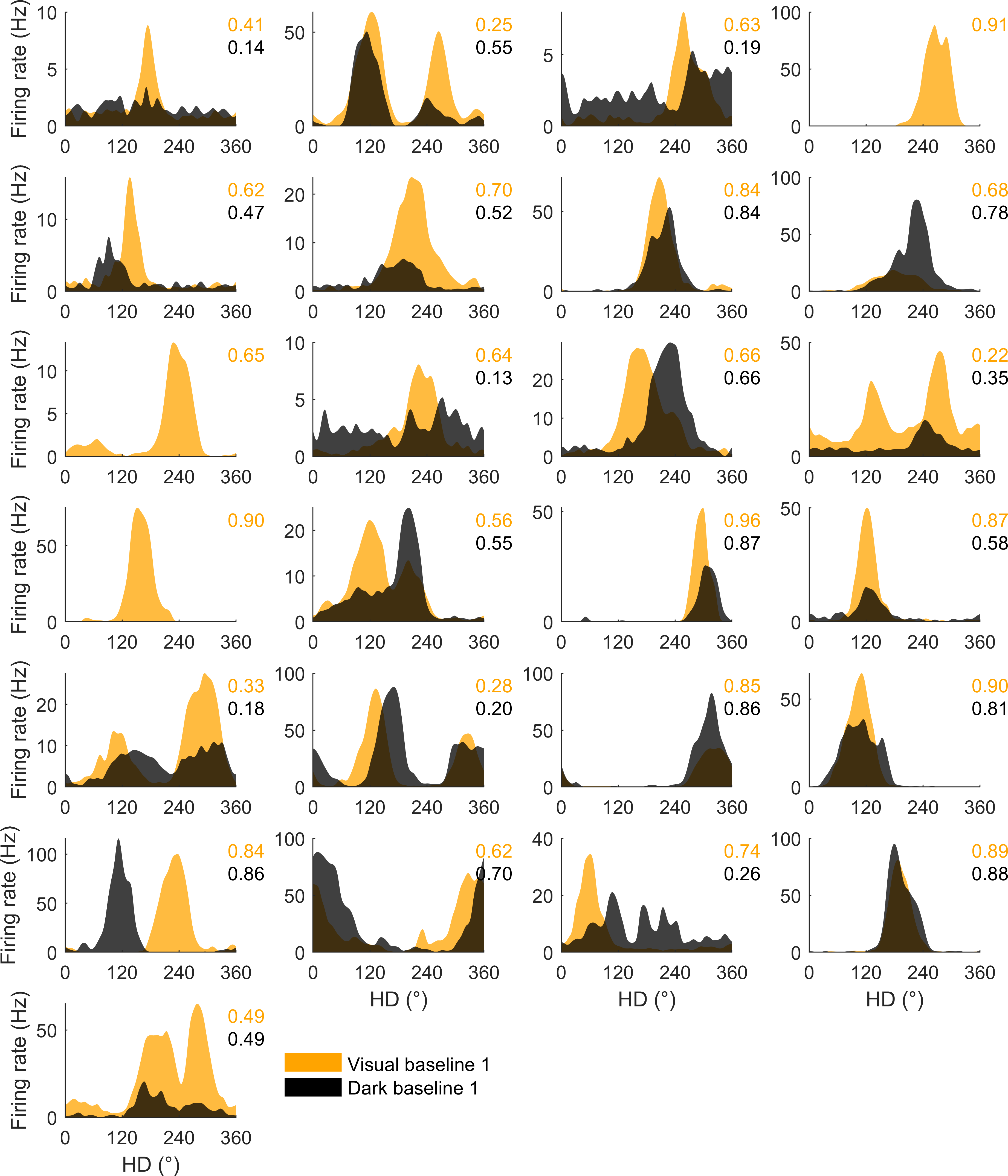

Supplement: Extended Data Figure 1-3 — Tuning curves for all HD cells recorded in Experiment 1. Yellow areas show the tuning curve for the first visual baseline session, black areas show the tuning curve for the first dark baseline session, if one was recorded. For all cells, cluster stability was confirmed in a subsequent baseline session. Top right-hand text with corresponding color shows the Rayleigh vector length. Download Figure 1-3, TIF file. [file enu-eN-NWR-0174-22-s04.tif]

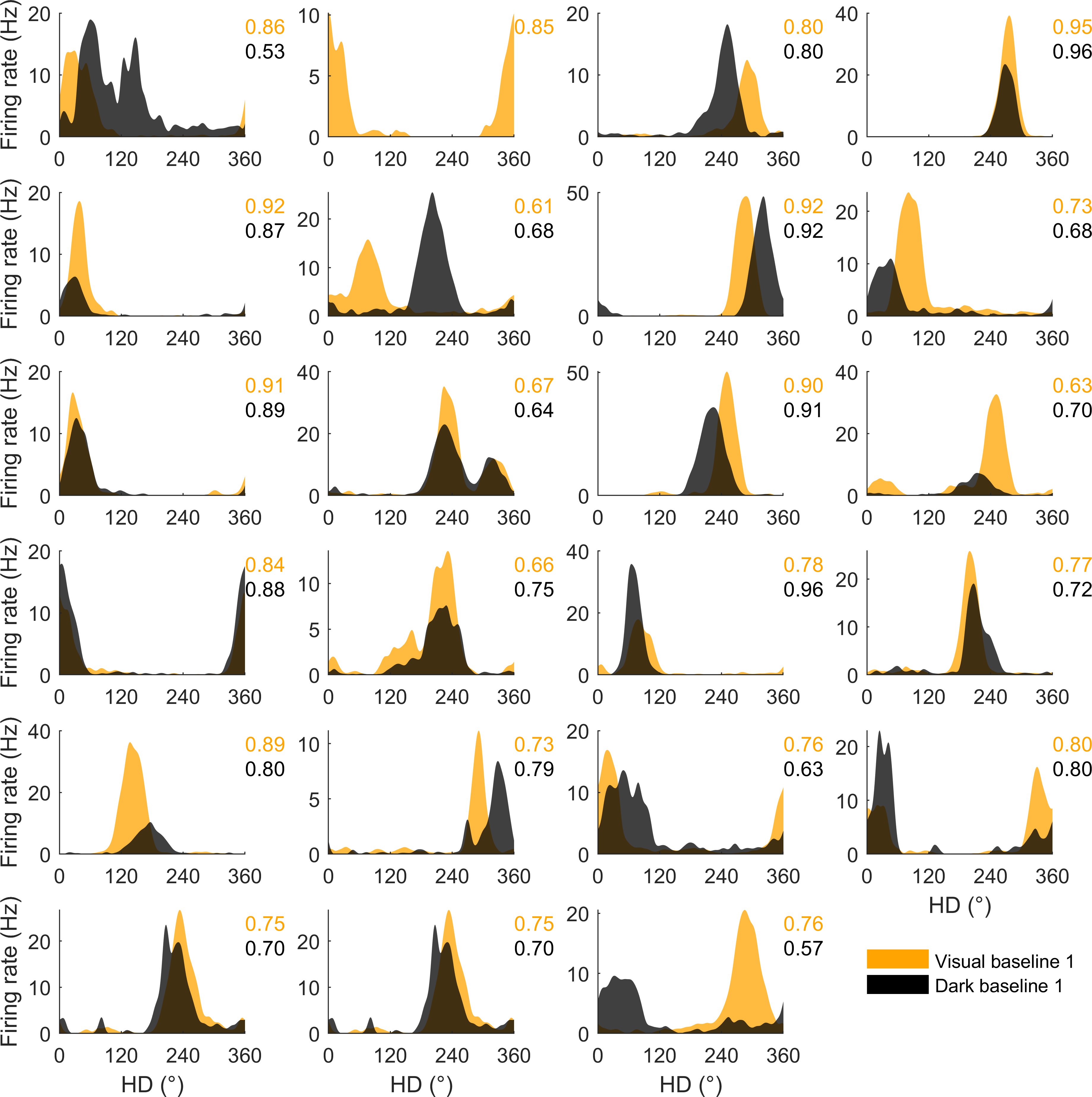

Supplement: Extended Data Figure 1-4 — Tuning curves for all HD cells recorded in Experiment 2. Yellow areas show the tuning curve for the first visual baseline session, black areas show the tuning curve for the first dark baseline session, if one was recorded. For all cells, cluster stability was confirmed in a subsequent baseline session. Top right-hand text with corresponding color shows the Rayleigh vector length. Download Figure 1-4, TIF file. [file enu-eN-NWR-0174-22-s05.tif]

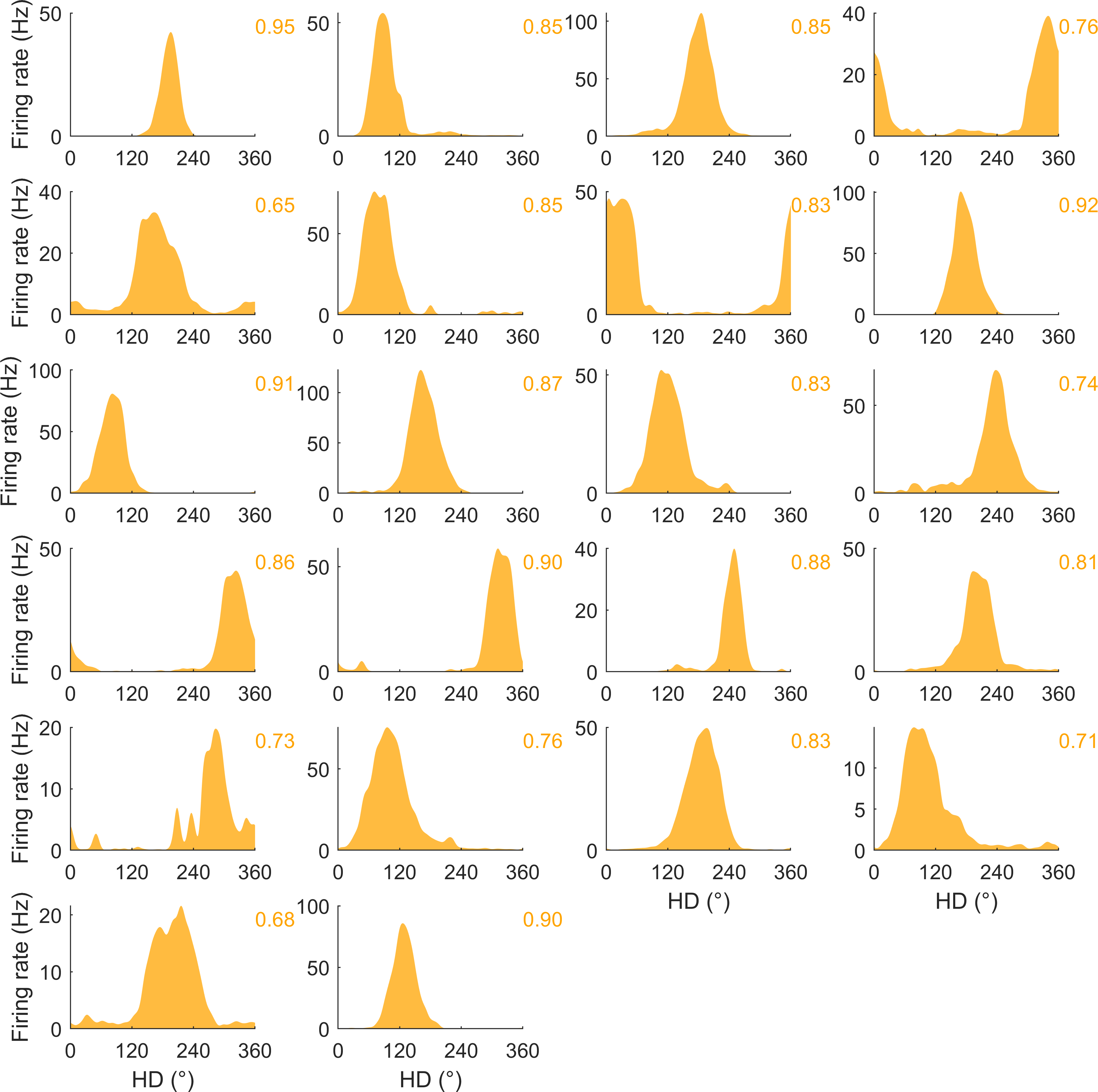

Supplement: Extended Data Figure 1-5 — Tuning curves for all HD cells recorded in Experiment 3. Yellow areas show the tuning curve for the first visual baseline session. Top right-hand text shows the Rayleigh vector length. One cell is missing, but this cell was verified as a HD cell by the experimenter before recording. Download Figure 1-5, TIF file. [file enu-eN-NWR-0174-22-s06.tif]

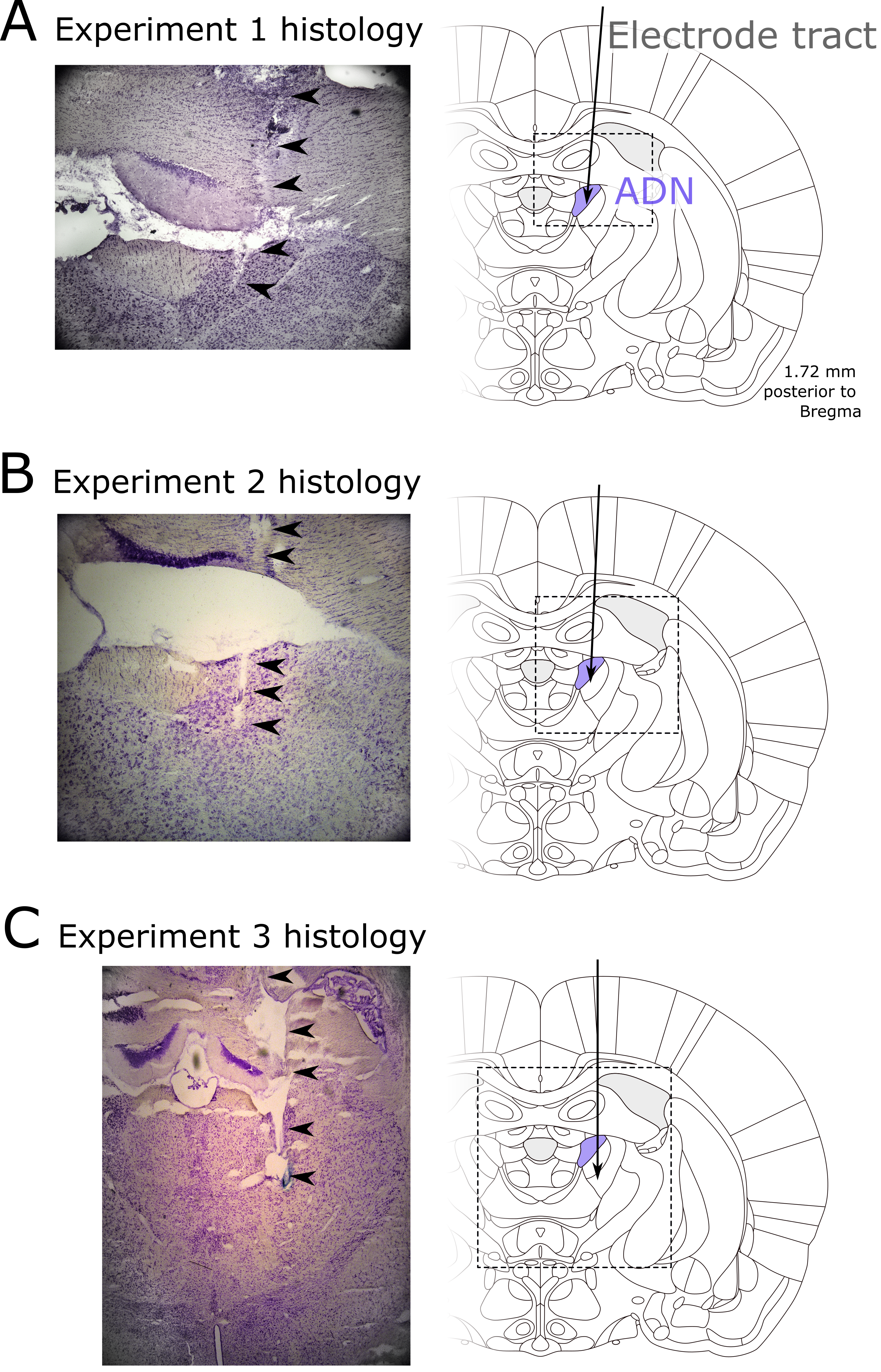

Supplement: Extended Data Figure 1-6 — Representative histology for Experiments 1–3. A, Representative Nissl-stained histology slide for an animal implanted in Experiment 1 with electrode track labelled (black arrows). To the right is a schematic diagram showing delineated brain structures (Paxinos and Watson, 2006) with the anterodorsal thalamic nucleus (ADN) shaded in blue and the reconstructed electrode track shown as a black arrow. B, C, Same as A but for Experiments 2 and 3. Download Figure 1-6, TIF file. [file enu-eN-NWR-0174-22-s12.tif]

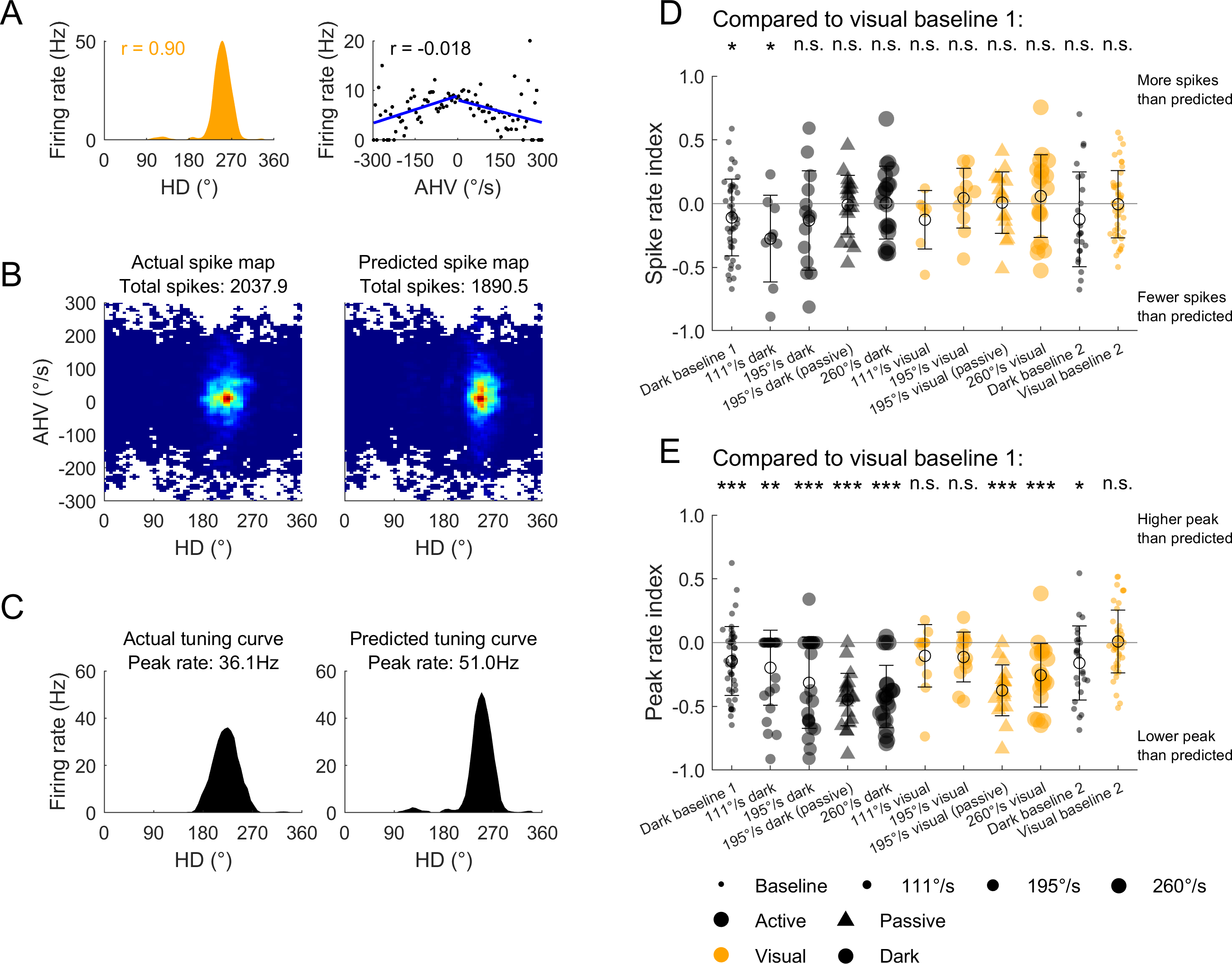

Supplement: Extended Data Figure 2-2 — A, Head direction (HD) tuning curve and angular head velocity (AHV) tuning curve for an example HD cell in visual baseline session 1. B, Left, the HD × AHV spike maps for the same cell but for dark baseline session 1. Right, The HD × AHV spike tuning histogram predicted for this this cell in dark baseline session 1 based on its activity in visual baseline session 1 (shown in A) and the animal’s AHV × HD sampling in dark baseline session 1. Bins that are empty in either map are shown as empty in both maps. C, Left, The HD tuning curve observed in dark baseline session 1. Right, The HD tuning curve predicted for dark baseline session 1. D, Spike rate index: (a − b)/(a + b), where a is the sum of the actual spike map and b is the sum of the predicted spike map, for every experimental condition. Low values indicate that fewer spikes were recorded than predicted while high values indicate the reverse. Values of zero indicate that a cell fired at exactly the rate predicted, which was the case in almost every condition. Text gives the result of Holm–Bonferroni corrected t tests comparing each group to the first visual baseline session (n.s. = p > 0.05, *p < 0.05, **p < 0.01, ***p < 0.001). E, Peak rate index: (a − b)/(a + b), where a is the peak firing rate in the actual HD tuning curve and b is the peak firing rate in the predicted HD tuning curve, for every experimental condition. Peak rate indices were generally less than zero, meaning that peak firing rates were lower than would be expected even when AHV tuning was taken into account. Text gives the result of Holm–Bonferroni corrected t tests as in D. Download Figure 2-2, TIF file. [file enu-eN-NWR-0174-22-s08.tif]

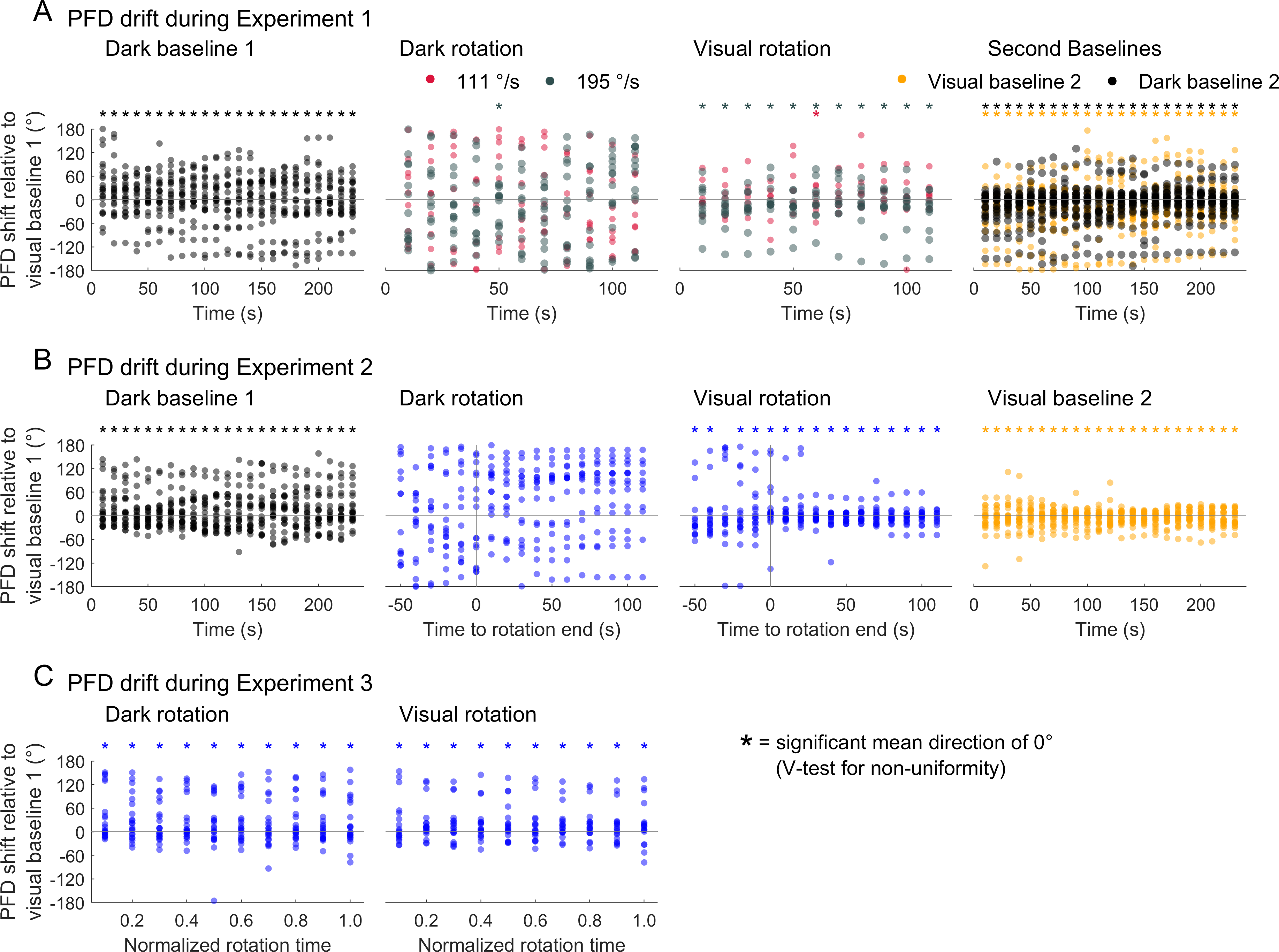

Supplement: Extended Data Figure 3-1 — PFD drift throughout baseline and rotation sessions. In all plots, filled markers represent HD cells. For Experiments 2 and 3, when a cell was recorded in one session type more than once, their responses were averaged to give a single value for each session type. Asterisks above each plot denote a significant mean direction around 0° (PFDs were consistent with visual baseline 1; tested using Holm–Bonferroni corrected v-tests), color corresponds to group color (see legends). The x-axes for Experiment 3 are truncated shortly after the end of the rotation period as the animal could no longer sample directional angles. Download Figure 3-1, TIF file. [file enu-eN-NWR-0174-22-s09.tif]

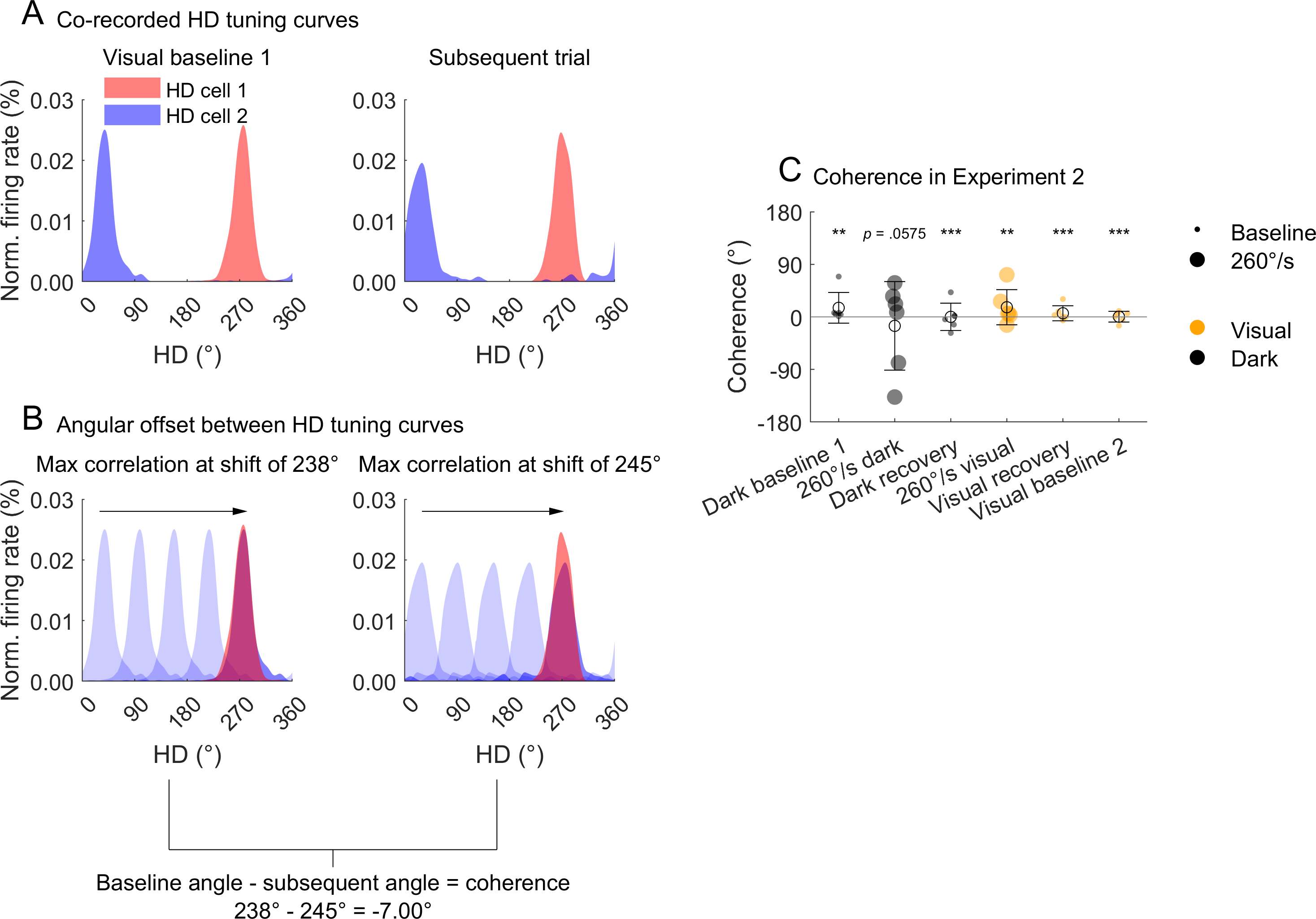

Supplement: Extended Data Figure 3-3 — A, A pair of co-recorded HD cells in the first visual baseline session and subsequent blindfolded baseline session. B, In both sessions, the tuning curve of cell 2 is rotated to find the angular shift at which it most highly correlates with cell 1. These angles are similar and deviate by only 7°, confirming that the cells remain coherent between sessions. C, Results when the same procedure is conducted on all six pairs of co-recorded HD cells in Experiment 2. Filled markers represent cell pairs, lines and circular markers denote circular mean and SD. The angle between tuning curves in each session is compared to the angle observed in the first visual baseline. The four blindfold rotation sessions and two visual rotation sessions are averaged to give a single value for each session type. The text above each group gives the result of a v-test of nonuniformity around 0°; significance here denotes a bias for values around 0°. Cell pairs were significantly coherent in all sessions, even during rotation, except when the rat was blindfolded and rotated. However, this group also exhibits a mean direction of 0° and is nearing significant directionality for this angle. Download Figure 3-3, TIF file. [file enu-eN-NWR-0174-22-s11.tif]

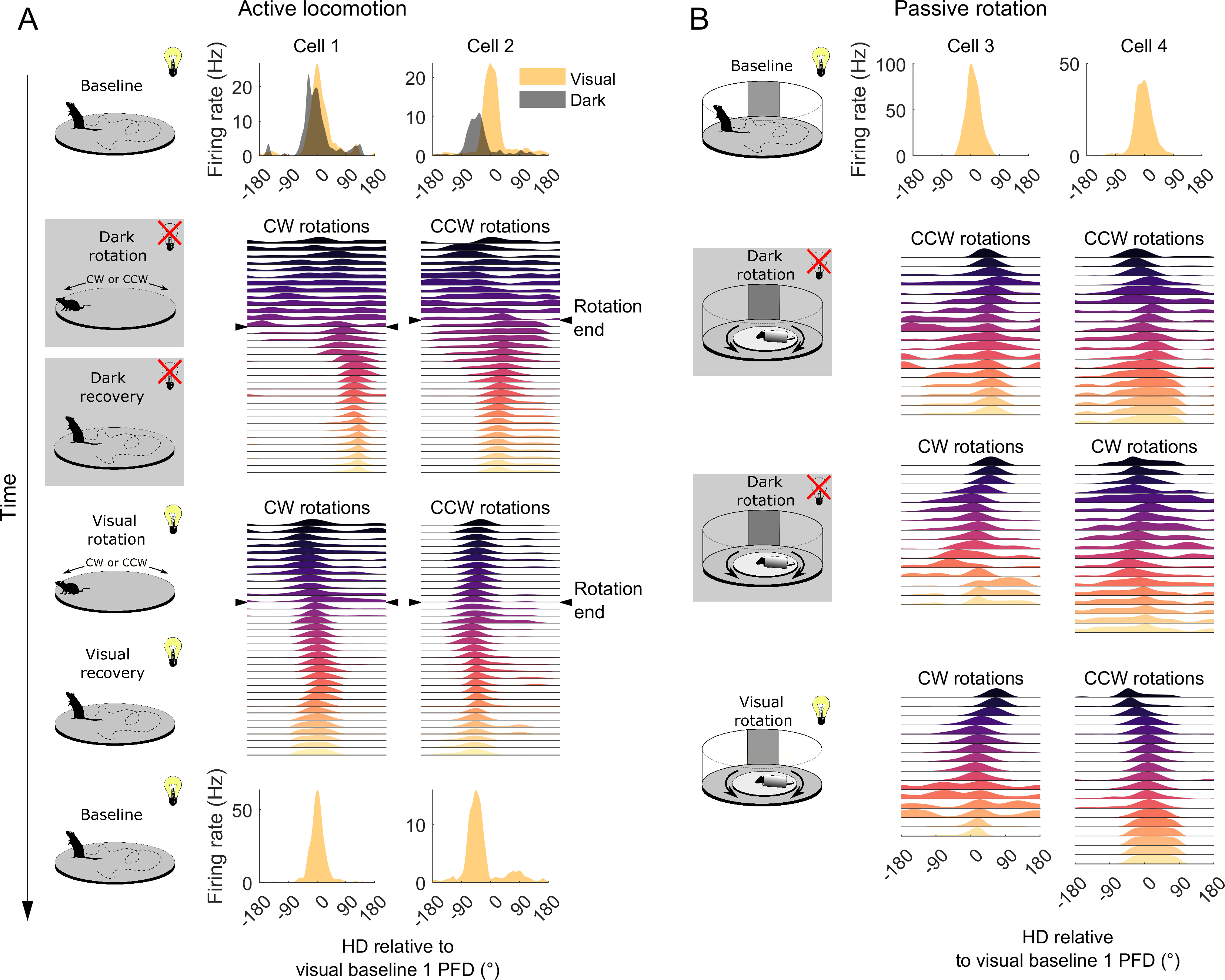

Supplement: Extended Data Figure 3-2 — Example HD cells showing the impact of disorientation on HD cell tuning. A, Left, Schematics representing the session types in Experiment 2, when rats were rotated unrestrained. Sessions are arranged temporally from top to bottom. Right, Two example HD cells, one per column. Tuning curves are given for baseline sessions (first and last rows). Windowed tuning curves (see Materials and Methods, Windowed tuning curves and parameters) are shown for rotation sessions (middle rows). Black horizontal arrows denote the end of the rotation period and the start of the recovery period. During dark rotations HD cells were disrupted, they lost directionality and their tuning curves were unstable. However, in the recovery period cells quickly regained these properties, even in the absence of visual cues, although their PFDs were offset randomly from baseline. During rotations in the light, cells remained largely unaffected throughout the rotation. B, Same as A but for Experiment 3 when rats were rotated while restrained. Here, directionality decreased quickly during rotation sessions, but cells maintained roughly consistent PFDs. Tuning curves also drifted in the direction of rotation at the start of rotation sessions. Download Figure 3-2, TIF file. [file enu-eN-NWR-0174-22-s10.tif]

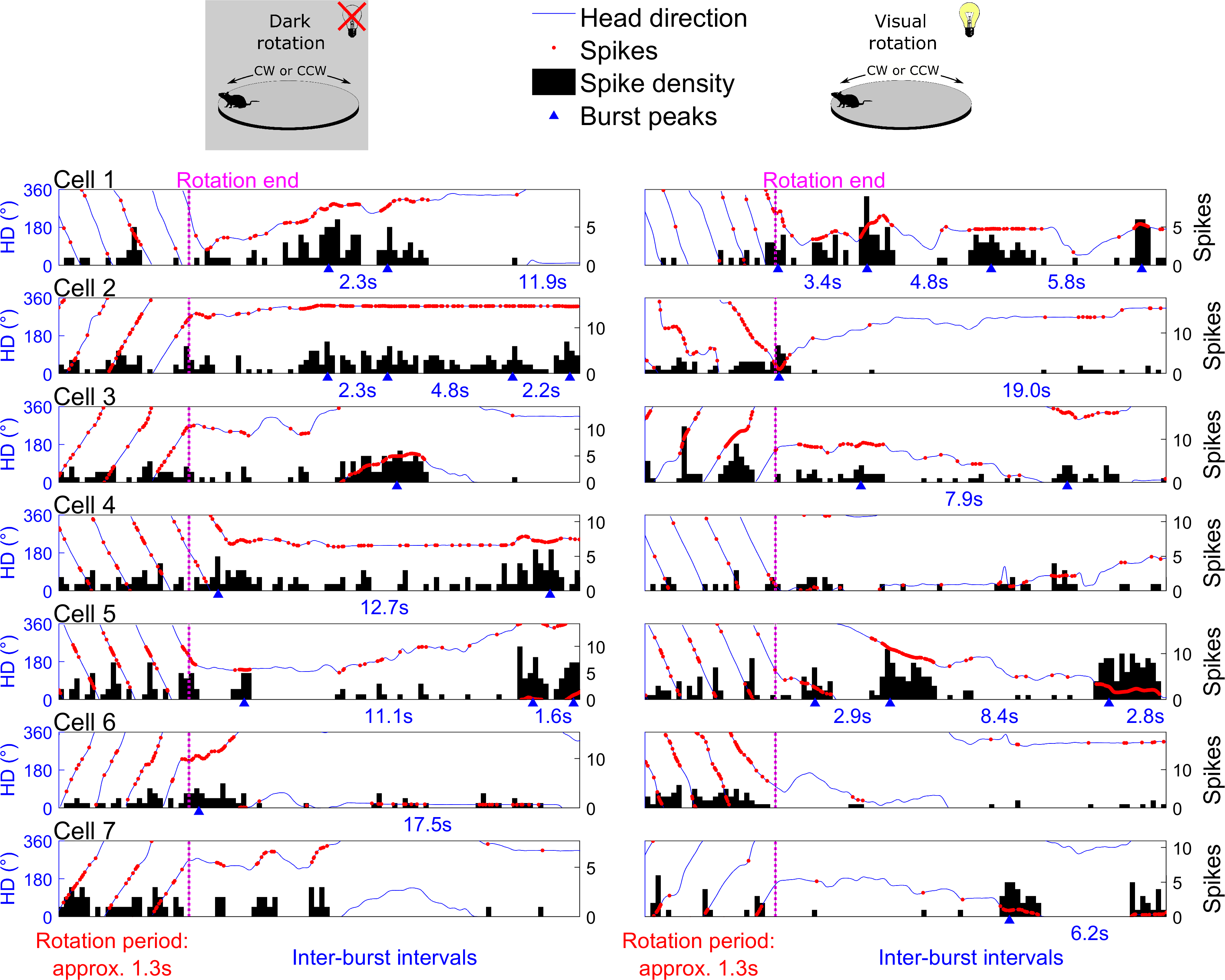

Supplement: Extended Data Figure 6-2 — Example cells showing an absence of postrotational bursting in Experiment 2, when rats were free to actively locomote during rotations. Example cells, one per row, left column shows activity in an example dark rotation session, right column shows activity for the same cell in an example rotation session in the light. Sessions are clipped to the end of the rotation phase (from 5 s before to 15 s after rotations ended). Blue lines denote the animal’s HD, red markers represent action potentials, black bars show a spike histogram (200-ms bins). Blue triangles denote detected spike bursts (Materials and Methods, Spike bursts), blue text between two triangles indicates the duration between these bursts. In both session types cells generally did not fire in bursts after the rotations ended. The bottom most cell (cell 7) is the only example of potential postrotational bursting that occurred in Experiment 2 during a dark rotation session. However, the number of spikes emitted was so low that these postrotational bursts did not meet our detection criteria. Download Figure 6-2, TIF file. [file enu-eN-NWR-0174-22-s14.tif]

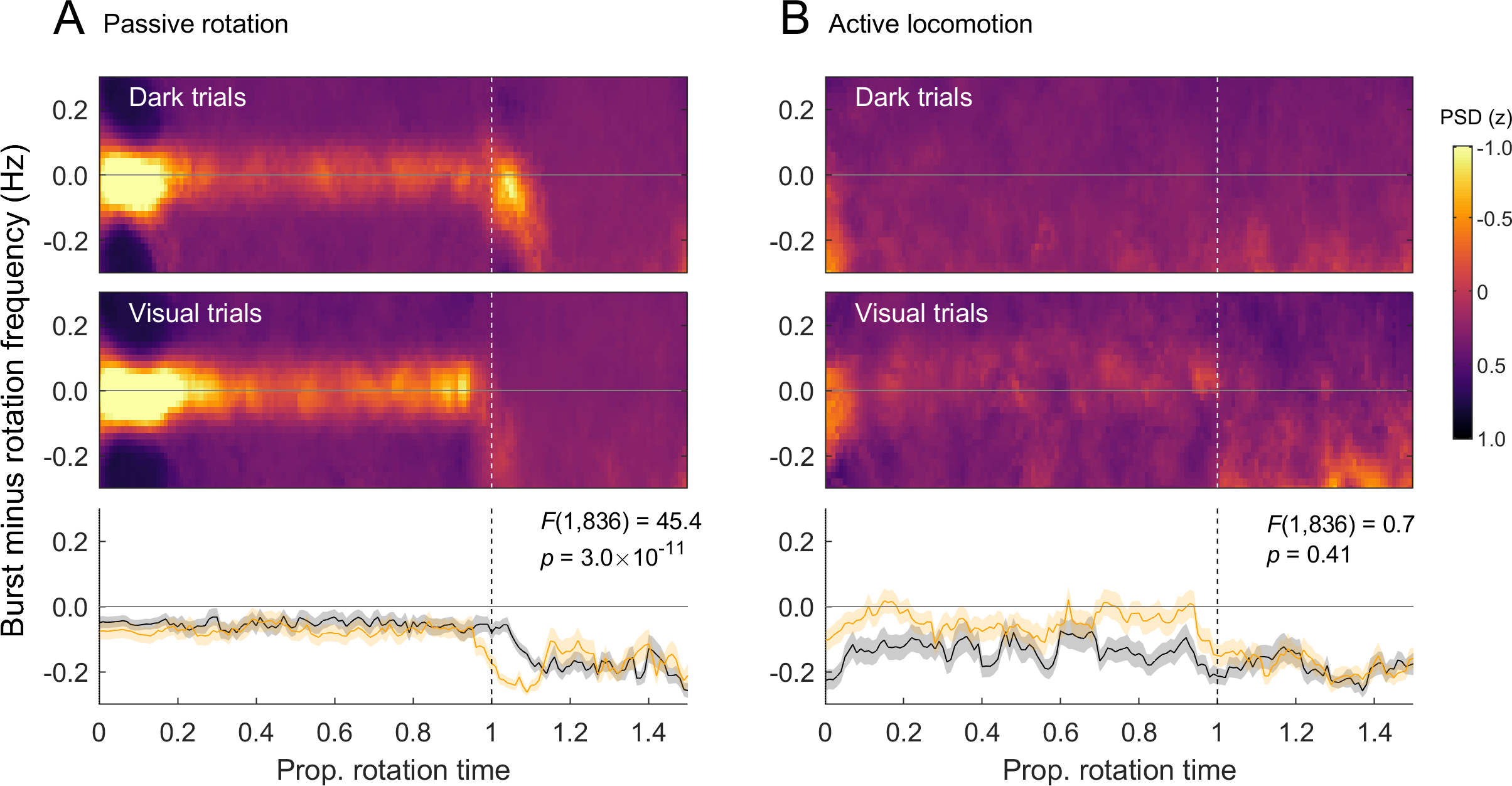

Supplement: Extended Data Figure 6-3 — Postrotational bursting analyzed by FFT (fast Fourier transform). A, FFT analysis of the kernel smoothed spike counts shown in Figure 6, but for all cells recorded during passive restraint (median across sessions then cells), separated by sessions in the dark (top) and light (middle). These spike counts are normalized such that 0 Hz represents the rotation frequency and time is expressed as a proportion of the rotation duration. High-power regions can be seen at the start of the rotation (t = 0), which correspond to the activity shown in Fig. 5. A second high-power region can be seen after the rotations have ended (t = 1) corresponding to the postrotation bursts shown in Figure 6A, but only for the sessions in darkness. Bottom, For each time point, the frequency associated with the highest power; this frequency decreased immediately at the end of light sessions but remained closer to the rotation frequency in dark sessions. The text shows the between-group difference result of a two-way ANOVA for data between t = 1.0 and t = 1.2. There was also a significant effect of time (F(18,836) = 2.12, p = 4.21 × 10^−3^, η2 = 0.04) and a significant interaction between time and group (F(18,836) = 6.21, p = 1.76 × 10^−14^, η2 = 0.11). B, Same as A but for cells recorded during rotations where animals were free to actively locomote. Postrotation bursting is absent. Bottom, There was no significant effect of time (F(18,760) = 0.47, p = 0.9700, η2 = 0.01) or interaction between time and group (F(18,760) = 0.51, p = 0.9554, η2 = 0.01). Download Figure 6-3, TIF file. [file enu-eN-NWR-0174-22-s15.tif]
